# Real-time insight into the multistage mechanism of nanoparticle exsolution from a perovskite host surface

**DOI:** 10.1038/s41467-023-37212-6

**Published:** 2023-03-29

**Authors:** Eleonora Calì, Melonie P. Thomas, Rama Vasudevan, Ji Wu, Oriol Gavalda-Diaz, Katharina Marquardt, Eduardo Saiz, Dragos Neagu, Raymond R. Unocic, Stephen C. Parker, Beth S. Guiton, David J. Payne

**Affiliations:** 1 Department of Materials, Imperial College London, Exhibition Road, London, SW7 2AZ UK; 2 Department of Applied Science and Technology, Politecnico di Torino, Corso Duca degli Abruzzi, 24, Turin, 10129 Italy; 3 Department of Chemistry, University of Kentucky, 505 Rose Street, Lexington, KY 40506 USA; 4 Department of Chemistry, Faculty of Science, University of Peradeniya, Peradeniya, 20400 Sri Lanka; 5 Center for Nanophase Materials Sciences, Oak Ridge National Laboratory, Oak Ridge, TN 37831 USA; 6 Department of Chemistry, University of Bath, Claverton Down, Bath, BA2 7AY UK; 7 School of Physical and Chemical Sciences, Queen Mary University of London, 327 Mile End Road, London, E1 4NS UK; 8 Composites Research Group, Faculty of Engineering, The University of Nottingham, Nottingham, NG8 1BB UK; 9 Chemical & Process Engineering, University of Strathclyde, Glasgow, G1 1XL UK; 10 Research Complex at Harwell, Harwell Science and Innovation Campus, Didcot, Oxfordshire OX11 0FA UK

**Keywords:** Synthesis and processing, Electrocatalysis, Solid-state chemistry

## Abstract

In exsolution, nanoparticles form by emerging from oxide hosts by application of redox driving forces, leading to transformative advances in stability, activity, and efficiency over deposition techniques, and resulting in a wide range of new opportunities for catalytic, energy and net-zero-related technologies. However, the mechanism of exsolved nanoparticle nucleation and perovskite structural evolution, has, to date, remained unclear. Herein, we shed light on this elusive process by following in real time Ir nanoparticle emergence from a SrTiO_3_ host oxide lattice, using in situ high-resolution electron microscopy in combination with computational simulations and machine learning analytics. We show that nucleation occurs via atom clustering, in tandem with host evolution, revealing the participation of surface defects and host lattice restructuring in trapping Ir atoms to initiate nanoparticle formation and growth. These insights provide a theoretical platform and practical recommendations to further the development of highly functional and broadly applicable exsolvable materials.

## Abstract (web summary)

The separation of nanoparticles from oxide hosts by exsolution forms the basis for catalytic and energy-related applications. Here, the authors elucidate the multistep mechanism of exsolution at perovskite surfaces by combining real-time electron microscopy and computational methods.

## Introduction

Recent developments in the field of nanomaterials have seen the emergence of a new “bottom-up” synthesis approach termed “exsolution”. Instead of decorating a support material with catalytically active nanoparticles (NPs), as in conventional “top-down” processes, the catalytic metal is incorporated as an ion in an oxide support during synthesis, to then diffuse to the host surface when the material is heat-treated in reducing conditions^1^, plasma-treated^2^, or treated with an applied electrical potential^3^, resulting in functionally-unique surface NPs. In recent years, the method has attracted widespread attention as it allows for the “in situ” generation of metal or alloy NPs^4^ with uniform size and distribution, with increased stability during operational conditions when compared to more traditional deposition techniques. This is due to the unique characteristic of “socketing” into the host crystal lattice, whereby exsolved NPs partially submerge into the surface of the host oxide, circumventing the deleterious agglomeration and coking common in deposited systems which reduce catalytic activity^5,6^. However, many questions remain regarding the mechanism of the nucleation and growth of exsolved NPs, and the rearrangement of the host oxide crystal structure during the controlled reduction of such systems, which, if solved, would dramatically improve structural control of exsolved materials, enable concept rationalisation, and lead to enhanced functional properties.

Although recent in situ/operando studies^7–14^ have been performed to gain insights into the exsolution mechanism, capturing the first stages of NP exsolution, where a sub-nanometre metal cluster nucleates before subsequently growing into a socketed NP, is extremely challenging, as reported in recent isothermal literature^8,9^. Visualising exsolving NPs during the initial atomic nucleation stage using a slow heating rate, as opposed to isothermal experiments, would slow down the process and allow the real-time observation of the first stages of NP exsolution. However, previous attempts at such studies have, to date, been unsuccessful.

Here, we provide atomic-scale, real-time insight in the early stages of nucleation and exsolution of Ir NPs from a stoichiometric Ir-doped SrTiO_3_ (STO) model structure in ultra-high vacuum (UHV), overcoming previous challenges, by applying in situ high-resolution high-angle annular dark field scanning transmission electron microscopy (HAADF-STEM). We observe atomic diffusion, nucleation sites, the evolution of the host crystal structure, and evidence of defects and, using computational simulations, ascribe their role in the early stages of nucleation. To investigate the phenomena observed during the evolution of the crystal structure of the host perovskite at lower temperatures we employ a machine-learning (ML) approach, which reveals that the evolution of a reconstruction at the surface of the monitored region prior to nucleation contributes to initiate the mechanism. These observations are combined to reveal the fundamental whole process of NP exsolution.

## Results

### The early stages of exsolution: from atoms to clusters

We started by preparing a model system of relevance for energy conversion technologies^15^, Ir-doped SrTiO_3_ (details in ‘Methods’); SrTiO_3_ has frequently been utilized as a host system for exsolution as it adopts the ABO_3_ perovskite structure, which facilitates incorporation of dopants and defects^16–18^, enabling a vast space of doped systems with different degrees of exsolution and functional properties. We identified and monitored the position of Ir dopant atoms in the lattice before in situ exsolution by low-temperature imaging, which confirmed that Ir atoms substituted for Ti atoms in the SrTiO_3_ cubic lattice (Supplementary Fig. 1 and Supplementary Note 1). A pair-potential surface scan was then carried out to study the range of phenomena occurring during the early stages of Ir NP nucleation. While the Ir dopants were initially substituted as Ir^4+^ species (from IrO_2_), ex situ X-ray photoelectron spectroscopy (XPS) characterisation showed Ir present in the 3+ oxidation state in the as-synthesized sample^15^. Density Functional Theory (DFT)-based thermodynamic analysis confirmed that experimental synthesis conditions (0.2 bar *p*O_2_, ≈1400 °C) favour the Ir^3+^ ions pair at Ti sites, and hence predicted the conditions when Ir^3+^ is stabilised over Ir^4+^ (analysis in Supplementary Information and Supplementary Fig. 2).

**Fig. 1:**
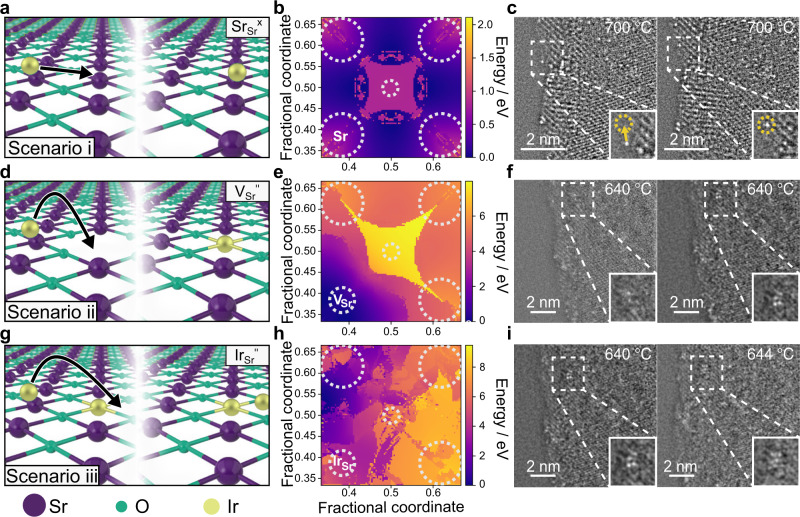
Evaluation of the Ir nanoparticle nucleation mechanism at the initial stages of exsolution in a stoichiometric Ir-doped SrTiO_3_ perovskite. Schematics of the three considered Ir migration scenarios of a slab model (001) SrTiO_3_ surface: **a** migration with no defects (scenario i, Sr_Sr_^x^), **d** migration with a surface Sr vacancy (scenario ii, V_Sr_”), **g** and migration where another Ir^+3^ ion has substituted a surface Sr ion (scenario iii, Ir_Sr_”). Ir, Sr, and O are represented by gold, purple, and green atoms, respectively. **b**, **e**, **h** Energy profiles of the Ir^3+^ ion over the three surface models represented in (**a**, **d**, **g**), respectively, with dotted circles to indicate the atomic positions (Sr: corner sites, O: centre, V_Sr”_ or Ir_Sr”_: bottom left in (**e**) and (**h**), respectively), as can be also visualised in the top-view atomic slab in Supplementary Fig. 2b. **c**, **f**, **i** Inverse fast Fourier transform HAADF-STEM images (after bandpass filtering) acquired during in situ monitoring of a [−3 1 2] zone axis grain at 700 °C (**c**), 640 °C (**f**), and 640, 644 °C (**i**) during in situ heating from 300 °C–700 °C at 2 °C min^−1^. The left and right images show experimental evidence for the modelled scenarios (i–iii) illustrated in (**a**, **d**, **g**), with boxed ROIs zoomed in the bottom right white boxes showing Ir single atom movement on the surface (highlighted by the yellow arrow and circles in (**c**)), Ir atom ‘trapped’ by a surface defect (**f**), and Ir cluster growth at the initial nucleation site over temperature (**i**), respectively. Source data are provided as a Source data file.

Figure 1 shows the three scenarios considered for the Ir^3+^ movement along a (001) SrO-terminated SrTiO_3_ surface: an ideal (non-defective) region (Sr_Sr_^x^; scenario i) in Fig. 1a; a region with a Sr vacancy (V_Sr_^”^; scenario ii) in Fig. 1d; and a region with an Ir ion already occupying a surface Sr site (Ir_Sr_^”^; scenario iii) in Fig. 1g. The generated surface energy profiles (Fig. 1b, e, h) resemble the geometry of the surfaces modelled, except for scenario iii. The surface energies are found higher at the corner sites (Sr sites), due to electrostatic repulsion, and at the centre (O site), because of the Ir^3+^ ion only interacting with one O^2−^ ion and the larger Ir-surface distances shown in the height profile (Supplementary Fig. 3a). On the ideal surface (scenario i), the positions at the Sr–Sr bridges have the lowest energies due to minimal Ir^3+^-Sr^2+^ repulsion and maximum Ir^3+^–O^2−^ interaction and are likely metastable resting positions for the Ir ions. The energy heatmap suggests that the Ir ion is mobile over ideal STO surfaces (with an energy barrier of moving between the metastable positions as low as ≈0.6 eV).

On a surface with a Sr vacancy (scenario ii) the high calculated energy barrier (≈5 eV) for the Ir ion to move away from the V_Sr_” suggests that the vacancy acts as a “trapping” site, as the required kinetic energy to overcome such energy barrier would be unlikely even at ≈1300 K. Once a mobile Ir ion has been trapped at a surface Sr vacancy, the surface becomes scenario iii. The complicated energy profile in Fig. 1h shows that the interaction between the two Ir^3+^ ions causes significant surface reconstruction. Additional structural images extracted from selected simulation positions (Supplementary Fig. 3b) show that when the approaching Ir is far from the lattice Ir, it ‘floats’ above the surface with a minimum height of 2.4 Å and high relative energy. Close to the lattice Ir position (dark-violet to orchid region in Supplementary Fig. 3b), the approaching Ir ion falls into the lattice, subsequently forming a stable Ir–O–Ir pair with a nearby lattice Ir and O. A calculated energy barrier of ≈3 eV indicates that the Ir ion is unlikely to move away from the Ir–O–Ir region once the pair is formed. This stable Ir–O–Ir pair can hence act as the foundation of further Ir cluster nucleation.

Overall, the surface energy scans suggest that a freely moving Ir^3+^ ion over ideal STO surfaces will be trapped if the ion meets a Sr vacancy. These trapped ions can then pair with other moving Ir ions, mediated by surrounding O, and are likely to become initial nucleation sites for further Ir cluster growth and reduction (schematic of the whole exsolution mechanism as outlined in this work in Supplementary Fig. 4). The atomic resolution in situ STEM images in Fig. 1c, f, i are experimental evidence of the modelled pair-potential scenarios (i–iii).

**Fig. 2:**
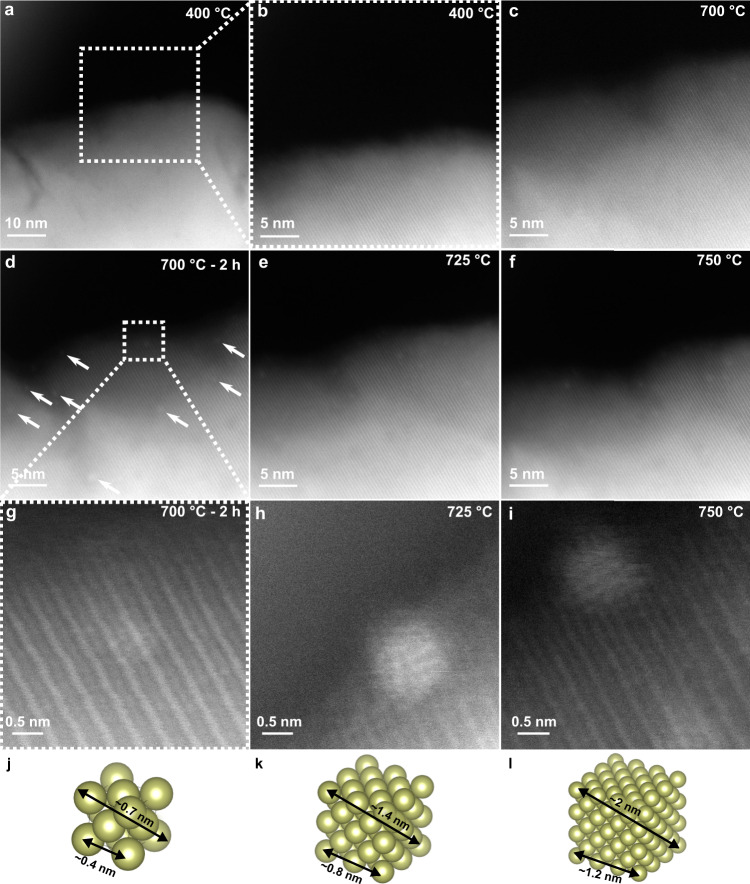
In situ monitoring of Ir nanoparticle nucleation during ultra-high-vacuum exsolution. **a**–**c** STEM micrographs of a SrIr_0.005_Ti_0.995_O_3_ grain monitored at 400 °C (**a**), magnified view of ROI at 400 °C (**b**), and 700 °C (**c**). **d**–**f** STEM micrographs after a 2 h dwell at 700 °C with small Ir clusters labelled with arrows (**d**), at 725 °C (**e**), and 750 °C (**f**). **g**–**i** High-resolution images of the Ir cluster boxed in (**d**) at 700 °C (**g**), 725 °C (**h**), and 750 °C (**i**). **j**–**l** Models of Ir unit cells measuring ≈0.7, ≈1.4, and ≈2 nm across in (**j**), (**k**), and (**l**), respectively. No socketing/epitaxy is possible in (**j**) with only 14 Ir atoms. Incipient epitaxy starts being observed for the array in (**k**), but the cluster is still small enough to migrate along the surface. Stronger epitaxy is observed (172 Ir atoms cluster) in (**l**) and socketing becomes more likely. Source data are provided as a Source data file.

**Fig. 3:**
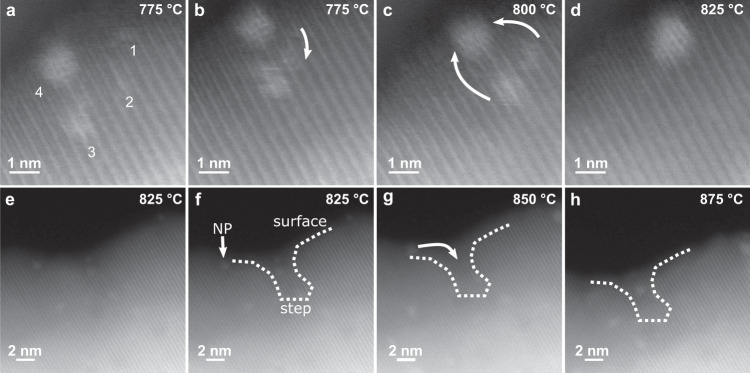
Movement of Ir atomic clusters on the surface of the host. **a**–**d** STEM micrographs of the surface of a SrIr_0.005_Ti_0.995_O_3_ grain monitored in situ at 775–825 °C. Ir atomic clusters (1–4) are labelled, with clusters 1 and 2 first coalescing, before further combining with clusters 3 and 4. **e**–**h** STEM micrographs of a stepped surface of a SrIr_0.005_Ti_0.995_O_3_ grain monitored in situ at 825–875 °C. The labelled nanoparticle (NP) travels along the surface and enters the surface ‘step’. The phenomenon presented in (**e**–**h**) and further movement of clusters similar to what presented in (**a**–**d**) are also visible in Supplementary Movie 1. Source data are provided as a Source data file.

To gain insights into the Ir clusters growth mechanism, further in situ experiments were performed by slowly increasing the heating rate to as closely mimic the synthesis of exsolved materials. Figure 2a–c displays no morphology or contrast changes when the sample is heated from 400 to 700 °C (or after a ≈2 h dwell at 400 °C, Supplementary Fig. 5), confirming that higher temperatures are required for exsolution of such doped perovskites^19–22^. After a 2 h dwell at 700 °C, brighter intensity ≈0.25–0.75 nm-sized clusters were noticeable (Fig. 2d, g), indicating Ir diffusion from the bulk and early nucleation. To study the Ir clusters evolution with temperature while retaining high resolution (HR)-imaging, the heating rate was reduced to 1 °C min^−1^. As suggested by the results of the pair-potential study, movement of the clusters was not observed in the monitored region of interest (ROI) within the measured temperature range. This confirmed that the growth of the nucleating clusters occurred by incorporation of further Ir atoms diffusing throughout the bulk and the surface of the material due to the increasing number of surface oxygen and A-site vacancies formed, also recently suggested on other systems^14,23^. The sub-nanometre clusters initially do not have a crystalline structure (Fig. 2g), as the clusters are smaller than one Ir unit cell (Fig. 2j), but NP-host grain lattice matching is observed as the size of the clusters increases at higher temperatures (Fig. 2h, i, k, l). While the above data demonstrate that NP growth occurs by diffusion of Ir atoms to the surface (at lower temperatures), Fig. 3a–d shows that, at higher temperatures (≥775 °C), growth can also occur via the coalescence of mobile Ir clusters already at the surface. As reported previously^8,12,23,24^, since the amount of exsolving metal in the lattice is one of the main factors limiting the degree of exsolution, if NP growth is observed once the metal supply has been exhausted, this can only be due to coalescence of growing nuclei. This had only been suggested based on ex situ indirect evidence of decreased populations and increased particle sizes at higher temperatures^12,25^, however, our work presents definitive evidence of the phenomenon.

Another type of mobility was also observed from 825 °C involving already formed surface NPs (Fig. 3e–h). During the timescale of this phenomenon the monitored NP migrates from its initial position on the surface until it reaches a more energetically favourable, stepped, defective region and “locks-in”. By increasing the temperature in UHV, diffusion of oxygen vacancies (V_O_) at the surface creates structural inhomogeneities, lowering the surface energy on such regions compared to flat surfaces, and resulting in preferential sites for nucleation^23^. The direct evidence confirms for the first time that NP migration can be expected during early stages of exsolution, and that socketing, the main characteristic of exsolved NPs, occurs at a later stage.

### Host structure evolution during in situ exsolution

Following the evolution of a ≈[301] Ir-doped STO surface from 400 to 700 °C, we observed the emergence of extra diffractogram spots in the fast Fourier transforms (FFTs) of the STEM images starting after a 2 h dwell at 700 °C (Supplementary Fig. 6d, h, l), in addition to the ones for the cubic STO sets of planes found for the sample at 400 °C (Supplementary Fig. 6a, e, i).

**Fig. 4:**
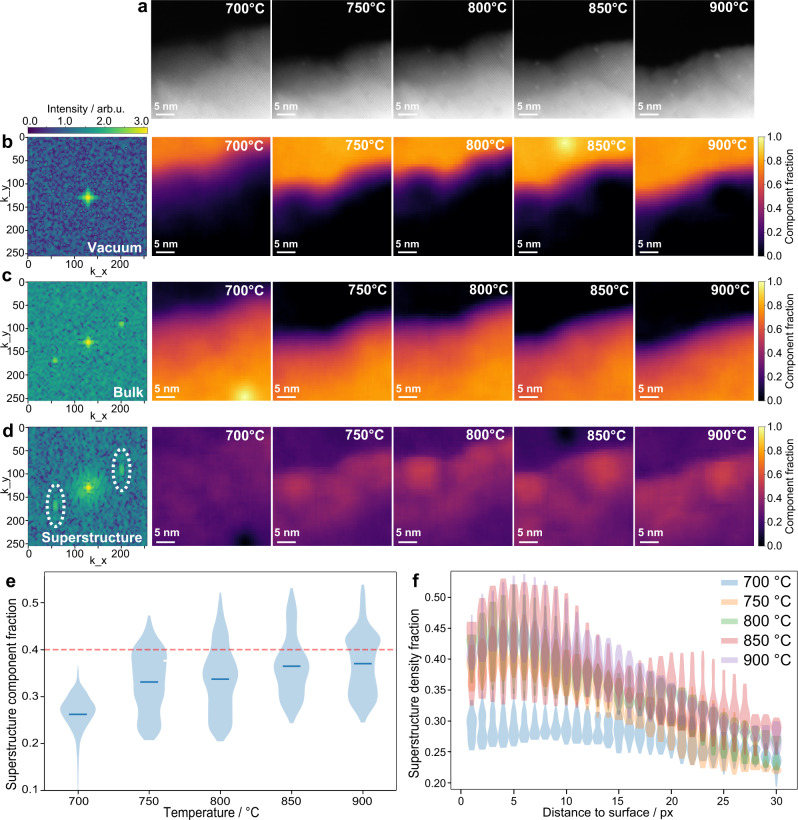
Analysis of in situ STEM images via the N-FINDR method. **a** STEM images of a 0.5% Ir-doped STO grain used for the N-FINDR analysis acquired in situ at 700–900 °C. **b**–**d** The three endmembers (vacuum (**b**), bulk (**c**), and superstructure (**d**)) extracted through the sliding FFT window approach, with corresponding resulting spatial abundances for each temperature targeted between 700 and 900 °C. The superstructure component is dashed circled for easier visualisation. **e** Plot of the density of the superstructure component as a function of target temperature. Red dashed line is at 40% fraction as a guide to the eye. **f** Violin plot of the density of the superstructure component (generated from the analysis of intensity histograms extracted from the raw STEM images) as a function of distance from the surface of the monitored grain for each targeted temperature between 700 and 900 °C, with different colours representing the different target temperatures from which the abundances were drawn. Original image is 1024 pixels across (i.e., 34.4 nm) and pixel size ≈0.72 nm. Individual plots for the data in (**f**) as well as a plot of the mean values for each temperature point are reported in Supplementary Fig. 12. Source data are provided as a Source data file.

When comparing the two HAADF-STEM images, the presence of the sub-nm Ir clusters found only after the dwell at 700 °C might be suspected to generate the extra spots, however, when investigating specific cluster areas, only the main perovskite signals were found (Supplementary Fig. 7), which excluded this possibility. It has been shown that structural changes such as vacancies clustering, ordering, or surface reconstructions, might generate superstructures^26–28^. In such cases, diffraction patterns (or, to a lower extent, FFTs) should show superlattice spots or elongated streaks depending on the degree of ordering^29^. To further investigate the structural changes generating the superlattice spots we employed a ML sliding FFT approach with matrix factorization^30,31^ (described in ‘Methods’), as superstructures are more easily visible within the Fourier domain than in real space, due to the intrinsic noise of STEM images. Figure 4a shows the raw micrographs acquired during in situ heating from 700 to 900 °C to which the sliding FFT algorithm was applied. We extracted the linear combination of ‘endmembers’ (‘pure’ spectra) that best represent the spectra contained in the raw micrographs. These were constrained to three, as the expected main phases: the vacuum, the bulk perovskite, and that corresponding to the superstructure. Indeed, Fig. 4b, c, d shows the endmembers and corresponding abundance maps related to these phases. Analysis of the progression of the ‘superstructure’ phase as a function of both temperature and spatial location revealed a gradual increase in its distribution with temperature (Fig. 4e), where its abundance first grows within the interior of the analysed grain and then increases in intensity towards the surface up to 900 °C (Fig. 4d; additional data in Supplementary Fig. 8). This is also visible in the plot of this endmember density as a function of distance from the surface per each temperature in Fig. 4f and Supplementary Fig. 12. Considering the edge of the monitored grain as a cross-sectional surface, our data suggest that a reconstruction or defect ordering is indeed happening at the sample surface starting from 700 °C in UHV, as Fig. 4f shows higher density near the surface and lower density elsewhere.

This evidence confirms the link between exsolution and surface reconstructions/ordering, allowing the identification of the first step involved in the exsolution mechanism for this Ir-STO system (Supplementary Note 2, Supplementary Fig. 4).

**Fig. 5:**
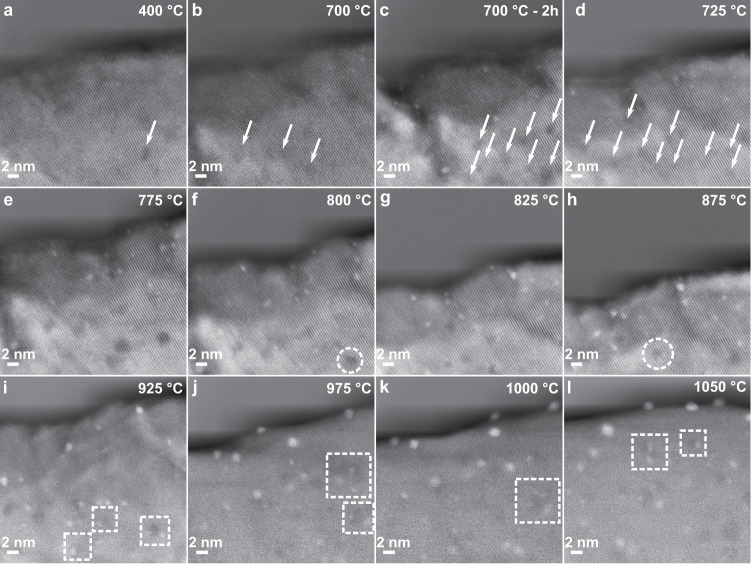
Clustering of defects observed during in situ exsolution. **a**–**l** STEM images acquired at different temperatures (from 400 to 1050 °C) during an in situ heating experiment of a 0.5% Ir-doped STO grain after applying a background subtraction algorithm. The arrows in (**a**–**d**) highlight the presence and increase in frequency with temperature of the defect clusters. The circles in (**f**) and (**h**) highlight some examples of faceting of the clusters, and the squares in (**i**–**l**) examples of the growth of exsolved NPs at defect cluster edges. Source data are provided as a Source data file.

Another structural feature, the clustering of defects, is evident in the host crystal in the in situ-monitored areas from 400–1100 °C (after applying a background subtracting algorithm; details in Methods and Supplementary Fig. 9). In Fig. 5, several dark-contrast features can be observed, starting at 700 °C (Fig. 5b–d), and evolving with an increase in size and density up to 900 °C, to then decrease in number from temperatures ≥900 °C. At intermediate temperatures (800–875 °C), faceting of the low-contrast features was also observed (Fig. 5f, h) followed by their morphology evolution to spherical shapes at temperatures ≥900 °C. In HAADF condition, lower contrast could be generated by: (i) a lack of heavier elements in the analysed region, and therefore a lack of Sr in the host STO, or (ii) a local decrease in thickness. For (i), annealing slightly-Sr-deficient SrTiO_3_ samples in vacuum has been reported to give rise to Sr vacancy clustering, with similar low-intensity regions for Sr-vacancy clusters reaching 5–7 nm in size^32–34^, similar to what observed in our system. For (ii), the distinct morphology and faceting, their number and density evolution over temperature, and the similar environment conditions to Sr-vacancy engineered systems^33,34^, make this unlikely. Furthermore, previous ex situ XPS characterization on this system showed presence of Sr surface deficiency^15^, further corroborating our interpretation of the results in Fig. 5. These clusters were found to be areas of dense NP nucleation, whereby Ir NPs were observed to grow at the edge of the dark-contrast defects (dashed squares in Fig. 5i–l). The data suggest that defects in the crystal structure, such as regions of vacancy clusters, are indeed originating a high local degree of exsolution. This demonstrates the importance of local defect concentration in tailoring exsolved systems, and, furthermore, that the degree of exsolution can be practically tuned by varying heating rates and intermediate dwell times.

### Socketing of exsolved NPs

**Fig. 6:**
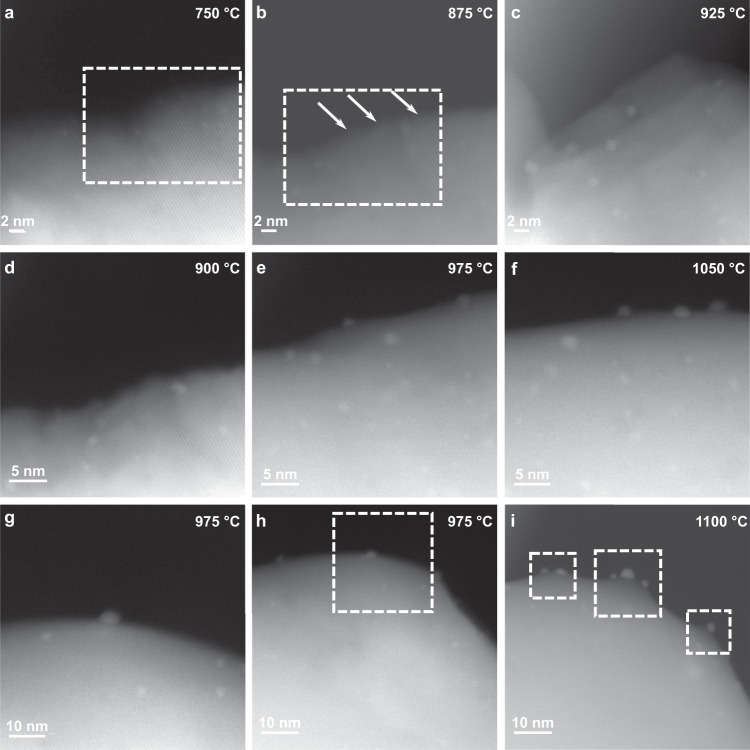
Morphology of the perovskite and socket evolution during in situ exsolution. **a**–**c** STEM micrographs showing crests formation during the in situ heating of a 0.5% Ir-doped STO grain. The rough surface visible at 750 °C in the squared region in (**a**) evolves into a more defined faceted morphology at 875 °C (**b**), as indicated by the arrows. Faceting of the sample surfaces becomes more prominent up to T = 925 °C (**c**). **d**–**f** The crests’ surface evolves into a sintered-like morphology at higher temperatures, with a rounder shape observed from 975 °C. **g**–**i** In situ observation of socket evolution of the same sample, from 975 °C (**g**, **h**), where no socket is visible, to 1100 °C, where pyramidal base structures have formed to embed the fully exsolved NPs (**i**), also presented in Supplementary Movie 2. Source data are provided as a Source data file.

Using in situ STEM, we monitored step-by-step the growth of the sockets around the exsolved particles. Once the particles nucleate within the host surface lattice^8^, the host grain undergoes a critical morphology change, resulting first in crest formation between 875 and 900 °C, as shown in Fig. 6a–d, and then in a smoother and rounder shape at temperature >950 °C (Fig. 6e, f). Under such experimental conditions we noticed ridges and pedestals forming and growing to support the faceted NPs (Fig. 6g–i). The observed variation in the grain surface morphology can be explained by the continuous depletion of oxygen from the system via increasing the temperature in low *p*O_2_ conditions, resulting in an increase in surface roughness, followed by a smoothening of the STO grains. The mass transport of Ir from the bulk to the surface and the strain imposed by the nucleating NPs on the system are, in fact, expected to cause a variation in shape and size of the host grain^7,35^, resulting in a seemingly sintered shape at temperatures >1000 °C. In our case, the ridges evolve to form a pyramidal pedestal, constituting the socket for our Ir NPs once they are already formed. Ex situ energy dispersive X-ray spectroscopy (EDS) analysis of the pedestals confirmed their compositional identity with the host perovskite (Supplementary Fig. 10), therefore excluding the possibility of a TiO_2_ or SrO decomposition product at the socket region. Accordingly, these results unequivocally demonstrate that socketing, the final stage in exsolution, only occurs once the particles are fully formed at the surface, and at relatively high temperatures for our stoichiometric system. The phenomenon is similar to the first stages of metal-droplet-catalysed growth of nanowires in the vapour-liquid-solid (VLS) mechanism^36–38^. Here, the matrix in the vapour phase condenses on the surface of a catalyst NP, to redeposit and epitaxially grow into a nanowire. As nanowires can only be grown where large droplets (or metal NPs, in our case) are present^39^, we propose that a similar mechanism is occurring at this stage of exsolution. Specifically, if the NP is smaller than the critical radius necessary to achieve steady-state growth of the nanowire, then further growth is not possible, leading to the formation of pyramidal base structures supporting the metal NPs on the host surface^38,39^. In our system, the highly dilute amount of Ir employed (0.5%) justifies the size of the exsolved NPs (≤7 nm), potentially explaining the pyramidal base growth observed. The lack of sufficient Ir supply does not allow to reach the catalyst supersaturation necessary for nanowire growth, which therefore cannot be induced^40^, resulting in the pyramidal bases, or exsolved particles sockets. As further supporting evidence, when exsolving from a higher-doped (5% Ir) STO sample, larger NPs were obtained (≤15 nm), with supporting pedestals of the biggest NPs resembling nanowires (Supplementary Fig. 10b–d).

## Discussion

Through a combination of in situ high-resolution STEM observations, computational simulations, and ML analyses, this work has revealed the evolution of nucleating Ir NPs from an STO host from the atomic level to their final nanoscale arrangement. We found that the initial nucleation is controlled by structural defects on the surface of the host material, determining the nucleation sites for further metal cluster growth. Under the common exsolution synthesis heating rate conditions the exsolving NPs were found to grow both by Ir diffusion from the bulk, and by coalescence of moving metal clusters. These clusters migrated toward energetically favourable surface defects and socketed only after being fully formed. We propose that the unique socketing characteristic of exsolved NPs is generated via a similar mechanism to VLS, where the metal NPs at the surface catalyse the growth of pedestals to lock themselves in place. The mechanistic insights described in this work prove the relationship between exsolution and crystal defects for Ir-doped STO, and we believe are generalizable to any similar dopant in STO-based exsolved systems. They highlight opportunities to effectively exploit structural and defect design strategies (including hitherto unexplored simple temperature control techniques) for the achievement of tailored exsolved NPs, with precise control over population, size, and morphology.

## Methods

### Sample preparation and in situ STEM experiments

SrIr_0.005_Ti_0.995_O_3_ perovskite was synthesized following a modified solid-state method^15^. High-purity SrCO_3_ (Sigma-Aldrich, ≥99.9%), TiO_2_ (Sigma-Aldrich, ≥99.98%), IrO_2_ (Sigma-Aldrich, ≥99.9%) reagents mixed in stoichiometric ratios and hand-ground in an agate mortar for 30 min were pressed into pellets and calcined at 1000 °C for 12 h in air (≈0.2 bar *p*O_2_), after which the pellets were ground into powder, which was mixed for 30 min, pelletised, and sintered at 1340 °C for 12 h in air. In situ STEM heating experiments were performed using an aberration-corrected Nion UltraSTEM100 STEM microscope combined with a Protochips Fusion MEMS-based heating stage. Specimen preparation was carried out by suspending the as-synthesised powder into high-purity 2-propanol followed by ultra-sonication (≈30 min). The samples were then drop-cast onto Protochips heating microchips then baked overnight at 80 °C in vacuum to eliminate any source of contamination prior to the in situ experiments. Imaging was performed at 100 kV voltage with a convergence semi-angle of 31 mrad, probe current of 0.5 nA and under ultra-high vacuum (10^−9 ^Torr). For all the experiments, the samples were first imaged at RT and at 400 °C to evaluate sample stability, which also ensured atomic position retainment and no exsolution occurring at low temperatures in the instrument environment. To separate the potential effect of continuous electron irradiation or beam damage coupling with the observed phenomena, and of the electron beam as an additional driving factor in exsolution, images were also acquired on at least three comparison areas for each sample, to evaluate whether beam-induced effect could be disregarded when imaging in HR-STEM mode^41^. STEM micrographs of several control areas captured at target temperatures ranging from 725–1100 °C are reported in Supplementary Fig. 11.

The exsolution of Ir nanoparticles was monitored in situ by imaging of the 0.5% Ir-doped SrTiO_3_ sample heated under 10^−9 ^Torr vacuum from room temperature to 1100 °C, temperature at which we observed full emergence of Ir nanoparticles when reducing ex situ^15^. The in situ experiments were performed with heating rates of 1 °C min^−1^, 2 °C min^−1^, or 5 °C min^−1^. Micrographs were analysed using a Gatan Digital Micrograph and a background subtraction algorithm filtering (details in Supplementary Fig. 9) was sometimes performed for better visualization of features of interest in the high-resolution images, such as to generate the images in Fig. 5.

### Computational details

#### DFT details

The host supercell used for simulation was a 3 × 3 × 3 SrTiO_3_ supercell, containing 135 atoms. The Ir ions were added as defect pairs and the doping concentrations were 7.4 atomic %. The Ir concentration used in experiment was ≈0.5 atomic %, which is much lower than the simulated concentration. The energetic errors associated with Ir concentration difference were confirmed to be small (analysis detailed in Supplementary Note 1) and do not qualitatively affect the results. The supercells were first relaxed at the PBESol+U^42^ (effective Hubbard *U*_eff_ = 4 eV) level of theory with the Vienna ab initio Simulation Package (VASP)^43–46^. The projector augmented wave (PAW) pseudopotential-based basis sets supplied in the VASP package were used with an energy cut-off value of 600 eV in these simulations. A 2 × 2 × 2 Monkhorst-Pack k-point mesh was used to sample the reciprocal space. The electronic cycle convergence criterion was set to be 10^−6^ eV, and the structural convergence criterion used was 0.01 eV Å^−1^. Spin polarization was also included. Antiferromagnetic (AFM) ordering was assumed when there were two Ir atoms present in the model, as testing suggested that AFM ordering gives lower energy.

These relaxed structures were then refined with the hybrid HSE06 functional using CRYSTAL17^47,48^ to achieve better energy description. Based on the linear combination of atomic orbitals (LCAO) method, double-zeta valence polarised (DZVP) atomic basis sets were used coupled with core pseudopotentials obtained on the CRYSTAL17 website. The Coulombic and exchange series were summed to the cut-off thresholds of 7, 7, 7, 9 and 30 as detailed in the CRYSTAL17 manual^48^. A slightly denser Monkhorst-Pack k-point mesh of 3 × 3 × 3 was used in the HSE06-based calculations to obtain better accuracy. The convergence criterion was set to be 10^−7^ Hartree. Spin polarisations were accounted for in the same manner as the VASP calculations.

#### Surface scan

Pair-potential-based surface scans were performed on slab models made of 3 × 3 × 4 SrTiO_3_ supercells with periodicity only in the x and y directions and top two unit-cell layers allowed to relax, while the other two unit-cell layers held fixed, representing the bulk. The surface calculations were carried out as implemented in GULP^49^. The cut-off range was 10 Å. The Ir^3+^–O^2−^ pair potential was custom-fitted against DFT relaxed surface slabs of Ir doped SrTiO_3_ (fitting details in the Supplementary Information), and the other pair potentials used were the Teter Buckingham pair potentials (detailed in Table 1)^50^. An Ir^3+^ ion was dragged through the region highlighted by the blue box in Supplementary Fig. 2b, with the ion’s x and y coordinates fixed but z coordinates relaxed. 10,000 xy positions were sampled in this region with a grid density of 100 × 100. The energy profiles and height profiles on these surfaces were then generated from the 10,000 calculations to illustrate possible migration paths, migration barriers and trapping possibilities of the Ir ion.

**Table 1:** Buckingham pair potentials used for pair potential-based simulations

Interaction	*A*_*ij*_/eV	*ρ*_*ij*_/Å	*C*_*ij*_/eV·Å^6^
Sr^1.2^–O^1.2−^	14566.637	0.245015	81.773
Ti^2.4^–O^1.2−^	23707.909	0.185580	14.513
O^1.2−^–O^1.2−^	1844.7458	0.343645	192.58
Ir^1.8^–O^1.2−^	24731.13737	0.182283	6.273148

#### N-FINDR analysis

For the N-FINDR analysis, the abundances were further constrained such that they sum to 1, i.e., $\sum {a}_{k}=1$, since these are physical spectra, and the proportion of each endmember that comprises each individual spectrum is sought. The spectral endmembers were then found by N-FINDR by first projecting the data onto a low-dimensional subspace (e.g., through principal component analysis) and then randomly selecting n spectra, which are used to construct a simplex. Through an iterative method the spectra that maximize the volume of the simplex are found, and these are the endmembers. A constrained least-squares fit is then utilized for calculation of the abundance maps.

### Reporting summary

Further information on research design is available in the Nature Portfolio Reporting Summary linked to this article.

## Supplementary information

Supplementary Information

Description of Additional Supplementary Information

Supplementary Movie 1

Supplementary Movie 2

Reporting Summary

## Data Availability

Source data have been deposited in the Zenodo database under accession code: 10.5281/zenodo.7474798. Source data are provided with this paper.

## Source data

Source Data
