# Supplementary material for: Real-time insight into the multistage mechanism of nanoparticle exsolution from a perovskite host surface
Source: Nat Commun. 2023 Mar 29;14:1754. doi: 10.1038/s41467-023-37212-6 (PMC10060596; doi:10.1038/s41467-023-37212-6)
Supplement: Supplementary file 1 — Supplementary Information [file 41467_2023_37212_MOESM1_ESM.pdf]

Supplementary Information

Real-time insight into the multistage mechanism of nanoparticle exsolution from a perovskite host surface

Eleonora Calì[1,2*†], Melonie P. Thomas[3,4†], Rama Vasudevan[5], Ji Wu[6,7], Oriol Gavalda-Diaz[1,8], Katharina Marquardt[1], Eduardo Saiz[1], Dragos Neagu[9], Raymond R. Unocic[5], Stephen C. Parker[6], Beth S. Guiton[3], David J. Payne[1,10*]

[1] *Department of Materials, Imperial College London, Exhibition Road, London SW7 2AZ, U.K.*

[2] *Department of Applied Science and Technology, Politecnico di Torino, Corso Duca degli Abruzzi, 24, Turin 10129, Italy.*

[3] *Department of Chemistry, University of Kentucky, 505 Rose Street, Lexington, Kentucky 40506, U.S.A.*

[4] *Department of Chemistry, Faculty of Science, University of Peradeniya, 20400, Sri Lanka.*

[5] *Center for Nanophase Materials Sciences, Oak Ridge National Laboratory, Oak Ridge, Tennessee 37831, U.S.A.*

[6] *Department of Chemistry, University of Bath, Claverton Down, Bath, BA2 7AY, U.K.*

[7] *School of Physical and Chemical Sciences, Queen Mary University of London, 327 Mile End Road, London, E1 4NS, U.K.*

[8] *Composites Research Group, Faculty of Engineering, The University of Nottingham, NG7 2QL, U.K.*

[9] *Chemical & Process Engineering, University of Strathclyde, Glasgow, G1 1XL, U.K.*

[10] *Research Complex at Harwell, Harwell Science and Innovation Campus, Didcot, Oxfordshire OX11 0FA, U.K.*

Email: e.cali14@imperial.ac.uk, d.payne@imperial.ac.uk

Supplementary Note 1. Computational details

Stability of Ir^{3+} and Ir^{4+} in $SrTiO_3$

The defect formation energies of Ir in $SrTiO_3$ were evaluated by DFT by considering Ir ions substituted using IrO_2 reactant (as done experimentally through solid-state synthesis). When adding Ir ions as defect pairs in a 3 × 3 × 3 host $SrTiO_3$ supercell, the DFT calculations showed that antiferromagnetic ordering results in the lowest formation energy. Supplementary Table 1 summarizes the possible reactions occurring when doping Ir into $SrTiO_3$ during a solid-state synthesis, and their respective defect formation enthalpies. Our results showed that, assuming 0 K and 1 bar oxygen partial pressure (pO_2), the Ir substituents are most likely to exist as Ir^{4+} ions sitting at Ti sites, due to having the lowest defect formation energy. *Ex situ* XPS characterization on this system, however, interestingly showed that Ir was present in the 3+ oxidation state in the as-synthesized sample. This peculiarity can be explained by the defect thermodynamic analysis based on DFT simulations, which confirmed that high temperatures can reduce Ir^{4+} ions to Ir^{3+} at Ti sites, following the reaction:

$$[\mathrm{Sr_{27}(Ir_2^{4+}Ti_{25})O_{81}}] \rightarrow [\mathrm{Sr_{27}(Ir_2^{3+}Ti_{25})O_{80}}] + 0.5\ \mathrm{O_2}\ (g)$$

Hence, whilst the Ir dopants were initially substituted as Ir^{4+} species, further DFT-based thermodynamic analysis confirmed these will be reduced in experimental synthesis conditions (0.2 bar pO2, ~1400 °C) and predicted the conditions when Ir^{3+} is stabilised over Ir^{4+}.

Supplementary Table 1. Defect thermodynamic analysis regarding the formation of Ir^{3+} and Ir^{4+} ions in bulk $SrTiO_3$ carried out based on DFT simulations. Four types of defect pairs were considered for the calculations: two Ir^{3+} ions occupying the Sr sites and compensated by a Sr vacancy ($2Ir_{Sr}^{\circ} + V_{Sr}^{\prime\prime}$); two Ir^{3+} ions occupying one Sr site and one Ti site respectively (Ir_{Sr}° + $Ir_{Ti}^{\prime}$); two Ir^{3+} ions occupying the Ti sites and compensated by one oxygen vacancy ($2Ir_{Ti}^{\prime} + V_{O}^{\circ\circ}$); and two Ir^{4+} ions occupying the Ti sites ($2Ir_{Ti}^{x}$). The corresponding reactions and defect formation energies are listed for each defect.

Defect	Reaction	ΔH / eV
$\mathbf{2Ir_{Sr}^{\circ} + V_{Sr}^{\prime\prime}}$	$2\ IrO_2 + 27\ SrTiO_{3(s)} \rightarrow [(Ir_2Sr_{24})Ti_{27}O_{81}] + 3\ SrO_{(s)} + O_{2(g)}$	13.391
$\mathbf{Ir_{Sr}^{\circ} + Ir_{Ti}^{\prime}}$	$2\ IrO_2 + 27\ SrTiO_{3(s)} \rightarrow [(IrSr_{26})(IrTi_{26})O_{81}] + SrTiO_{3(s)} + O_{2(g)}$	6.248
$\mathbf{2Ir_{Ti}^{\prime} + V_{O}^{\circ\circ}}$	$2\ IrO_2 + 27\ SrTiO_{3(s)} \rightarrow [Sr_{27}(Ir_2Ti_{25})O_{80}] + 2\ TiO_{2(s)} + O_{2(g)}$	2.251
$\mathbf{2Ir_{Ti}^{x}}$	$2\ IrO_2 + 27\ SrTiO_{3(s)} + 2\ SrO_{(s)} \rightarrow [Sr_{27}(Ir_2Ti_{25})O_{81}] + 2\ SrTiO_{3(s)}$	-0.961

Using the NIST handbook for gaseous oxygen thermodynamic data, calculations on the reduction energy were hence performed considering our synthesis conditions in air (0.2 bar pO_2), and the results are plotted in Supplementary Fig. 2a. The DFT-based analysis suggested that it takes 1.606 eV to reduce one Ir^{4+} ion to Ir^{3+} ion (or 3.212 eV per formula reaction) at 0 K and 1 bar pO_2. The plot further shows a temperature of ~700 K is sufficient for the Ir^{4+} ion pair reduction to an Ir^{3+} ion pair in the $SrTiO_3$ host, where the $2Ir_{Ti}^{x}$ defect state changes into the $2Ir_{Ti}^{\prime} + V_{O}^{\circ\circ}$ state, and the Ir^{4+} ions are reduced to Ir^{3+} while releasing an O_2 molecule during the process at 0.2 bar pO_2 pressure. The 700 K temperature is substantially lower than our synthesis temperature conditions, therefore explaining the experimentally detected Ir^{3+} in our samples, despite the starting +4 oxidation state of the Ir reactant employed and of Ir_2O_3 not being a stable compound. Once reduced, the Ir^{3+} ions will require oxygen atoms or electron acceptor species to be reoxidized to Ir^{4+}. Due to the required oxygen gas release and the associated reaction kinetics, the reduction is expected to gradually occur starting from Ir ions at or close to the surface.

Fitting of Ir – O Buckingham pair potential

The Ir – O potential is fitted to DFT relaxed Ir doped $SrTiO_3$ slab models with GULP.[8] The Ir doped $SrTiO_3$ slabs constructed for DFT relaxation are similar to those used in the pair potential based surface scanning simulation. The host slabs consisted of 3 × 3 × 4 $SrTiO_3$ supercells terminated with (100) surface, with the bottom two layer fixed to resemble the bulk $SrTiO_3$ interaction. Three defect scenarios were considered: perfect lattice, lattice with one surface Sr vacancy, and lattice with one Ir replacing a surface Sr vacancy. For each defect scenario, an additional Ir is initially placed above the surface corner Sr site/Sr vacancy site/Ir_{Sr} site, above the centre of the Sr-Sr bridge and above the centre of the surface. The initial height of the additional Ir is 2 Å from the surface. Depending on the defect scenario, extra surface cations (Sr or Ti) far away from the Ir ion are removed to maintain charge neutrality assuming all the Ir are in the +3 ionic state. In total, 9 slabs are relaxed using the PBESol+U functional implemented in VASP. All computational details are the same as described in Methods, except that a 2 × 2 × 1 Monkhorst-Pack k-point mesh was used to account for the slight anisotropy in the slab calculations.

Error associated with the Ir defect concentration

The Ir concentration used in experiment is ~0.5 atomic %, which is much lower than the concentration simulated. This is mainly due to the high computational cost required to reproduce the low dopant concentration. A large host super lattice containing at least 2000 atoms is required to achieve an Ir dopant concentration of 0.5 atomic % which is impractical for simulations given that computational cost increase exponentially with host size. To understand the scale of the errors associated with the dopant concentration, simulations were carried out to calculate the Ir^{4+} to Ir^{3+} reduction at the Ti sites in 4 × 4 × 4 and 5 × 5 × 5 host $SrTiO_3$ supercells, with Ir dopants at 3.1 atomic % and 1.6 atomic %

respectively. The supercells were first relaxed at the PBESol functional level, then refined with a hybrid HSE06 functional to achieve a better energy description, as detailed in the main text.
The results are presented in Supplementary Table 2:

Supplementary Table 2. Ir reduction energy from +4 to +3 oxidation state at Ti sites in 3 × 3× 3, 4 × 4 × 4, and 5 × 5 × 5 host $SrTiO_3$ supercells.

**Cell size**	**Ir atomic concentration / %**	**Reduction energy per Ir ion / eV**
3x3x3	7.4	1.606
4x4x4	3.1	1.591
5x5x5	1.6	1.565

The results suggest that the Ir reduction energy decreases slowly with lower Ir dopant concentration. With the concentration approximately halved, the reduction energy drops by 0.01-0.03 eV. This error range will not affect our conclusion assuming the trend follows until 0.5% Ir atomic concentration, and, accordingly, these simulation results will still provide at least a qualitative understanding of the experimental phenomena. On the other hand, the computational cost increases exponentially with larger host size. The 3 × 3 × 3 cell requires 288 CPU cores and approximately 72 hours to complete one defect simulation, while the 4 × 4 × 4 cell requires 576 CPU cores and the 5 × 5 × 5 cell takes 2304 CPU cores to simulate one defect case in 72 hours. It is unlikely that we can compute the 0.5 atomic % Ir cases given the computational resources available to us.

Supplementary Figures

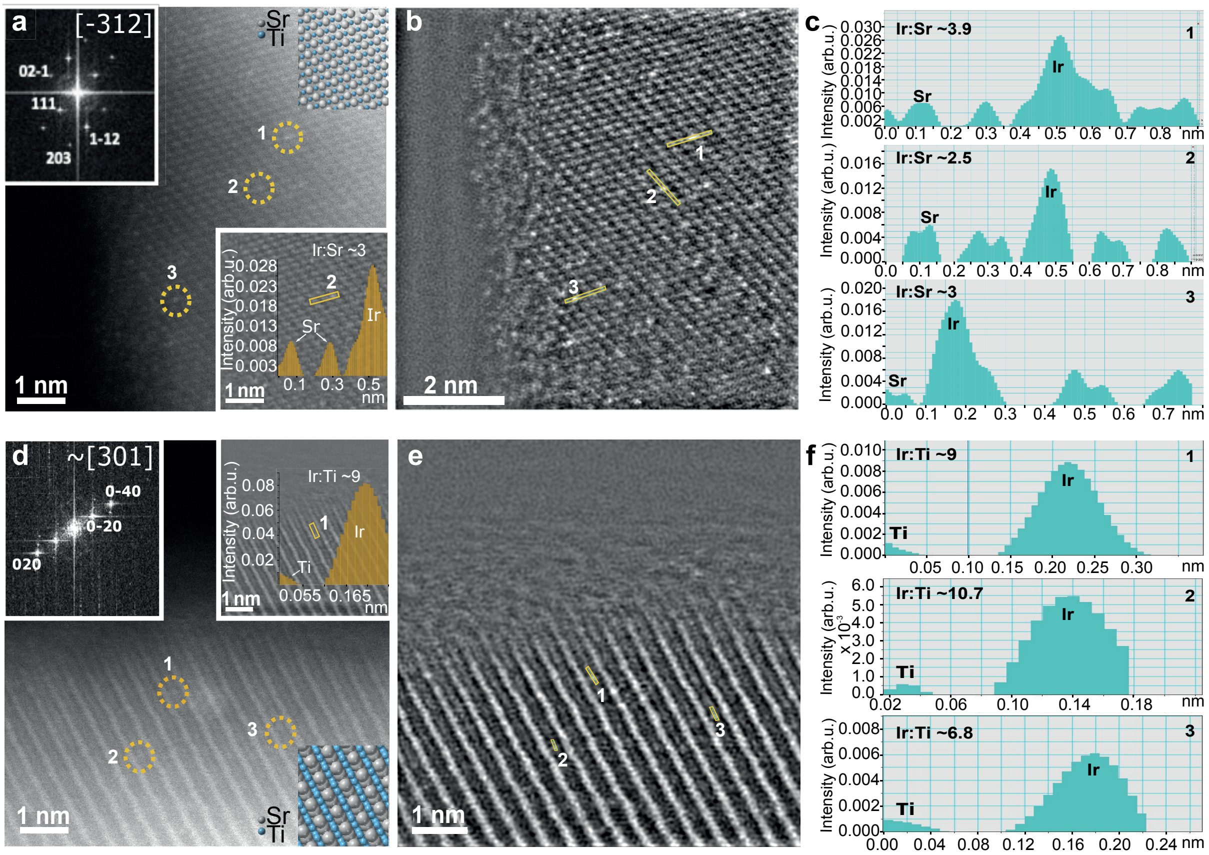

Supplementary Fig. 1. **The position of Ir dopant atoms in the lattice before in situ exsolution identified and monitored by imaging at low temperature (RT before in situ heating and 700 °C)**. The analysis of the brighter intensity atoms imaged on a [-312] zone axis and a slightly off-zone axis ~[301] Ir-doped $SrTiO_3$ grain during different in situ experiments is based on HAADF contrast, for which the intensity (I) is proportional to atomic number ($I \propto Z^{1.6-1.9}$),[9] and confirms the Ir nature of the brighter-contrast atoms visible in the HR-STEM images. These are found to substitute the Ti atoms in the cubic lattice. **a, b, d, e,** HAADF-STEM micrographs of (a,b) a [-312] and (d,e) ~ [301] oriented $SrIr_{0.005}Ti_{0.995}O_3$ grains acquired in UHV during the in situ heating experiments at RT and 700 °C, respectively. The analysis based on the intensity ratios between Ir:Sr atomic numbers and Ir:Ti atomic numbers considering $I_{HAADF} \propto Z^{1.6\text{-}1.9}$ in the insets of (a) and (d), respectively, allows to identify the Ir atoms in the lattice (brighter intensity atoms circled in gold), with an Ir:Sr ratio of 3 (a) and an Ir:Ti ratio of 9 (d); **c, f,** The detailed analysis based on Z contrast of the areas highlighted by the lines in (b) and (e) is reported in (c) and (f), respectively, where three intensity profiles per each of (b) and (e) are reported as well as the intensity ratios (Ir:Sr for (c) and Ir:Ti for (f)). Source data are provided as a Source Data file.

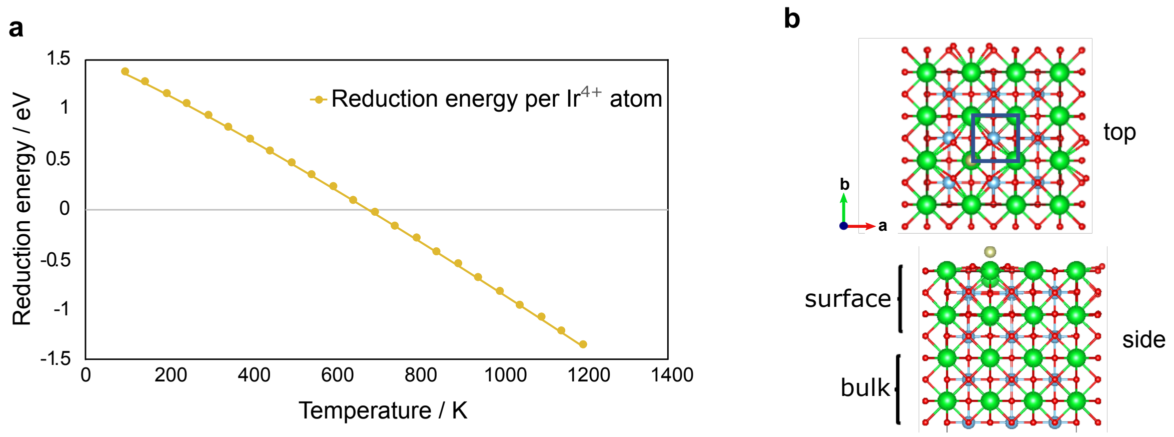

Supplementary Fig. 2. Ir^{3+} ion pair stabilisation during sample synthesis. (a) Plot of the results of DFT calculations on the reduction energy performed considering real synthesis conditions in air (0.2 bar pO_2). The results show that a temperature of ~700 K is sufficient for the Ir^{4+} ion pair reduction to an Ir^{3+} ion pair in the $SrTiO_3$ host. (b) Top and side view examples of the slab model created for the Ir ion migration scenarios at the (001) $SrTiO_3$ surface used for the MD calculations of the three considered migration paths described in Results section "The Early Stages of Exsolution: from Atoms to Clusters". Source data are provided as a Source Data file.

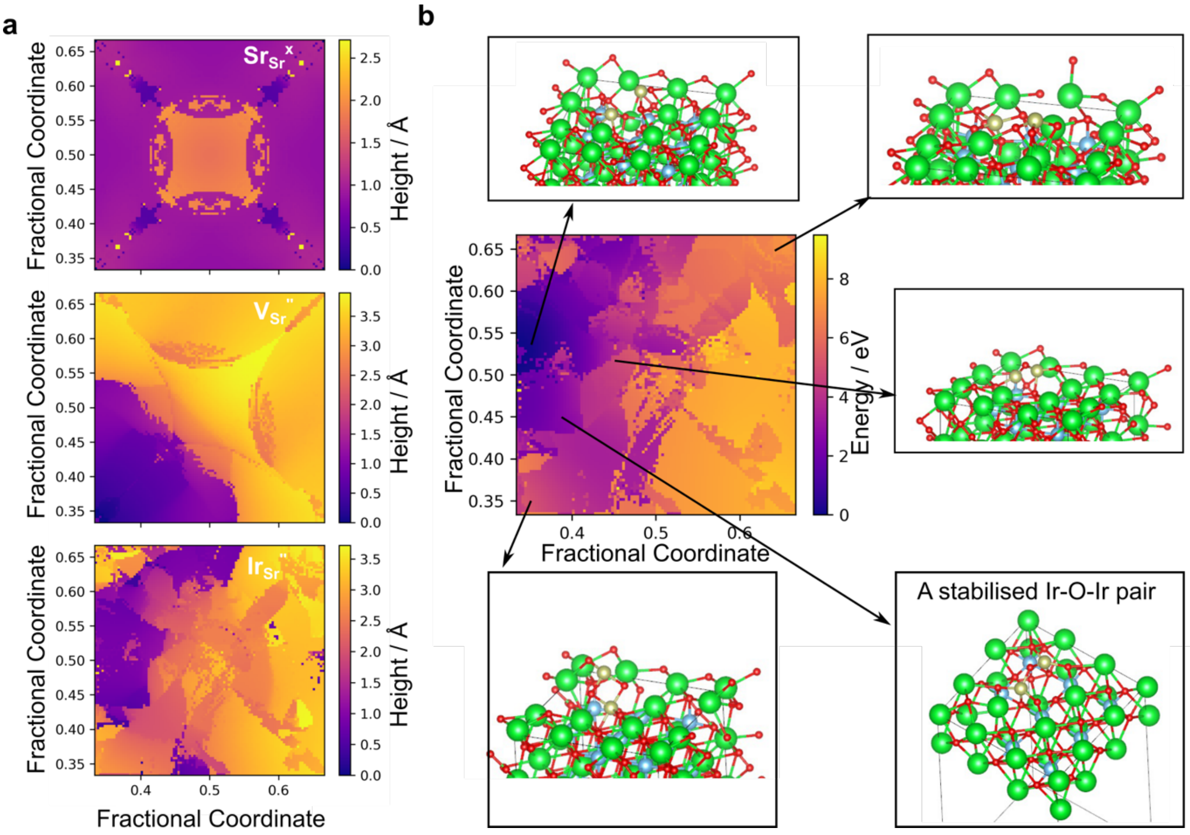

Supplementary Fig. 3. Height profiles and insight into the energy profile of scenario (iii). (a) Height profiles for the three scenarios described in Results section "The Early Stages of Exsolution: from Atoms to Clusters". (b) Energy profile for scenario (iii) discussed in the Results section "The Early Stages of Exsolution: from Atoms to Clusters" with selected structure images extracted for selected simulation positions. When the approaching Ir is far away from the lattice Ir, it 'floats' above the surface with a minimum height of 2.4 Å and high relative energy. Close to the lattice Ir position (dark-violet to orchid region in the energy profile map), the approaching Ir ion falls into the lattice and forms a relatively stable Ir-O-Ir pair with a nearby lattice Ir and O. It requires ~ 3 eV for the approaching Ir to move away from the region which allows the formation of Ir-O-Ir pairs, therefore this Ir ion is unlikely to move away once the pair is formed. This 'socketed' stable Ir-O-Ir pair can act as the foundation of further Ir cluster nucleation. It also hints at how the exsolved Ir metal particle is socketed at the perovskite surface once the oxygen in the pair is reduced and evaporated from the surface. Green atoms are strontium, blue atoms are titanium, red atoms are oxygen, and gold atoms are iridium. Source data are provided as a Source Data file.

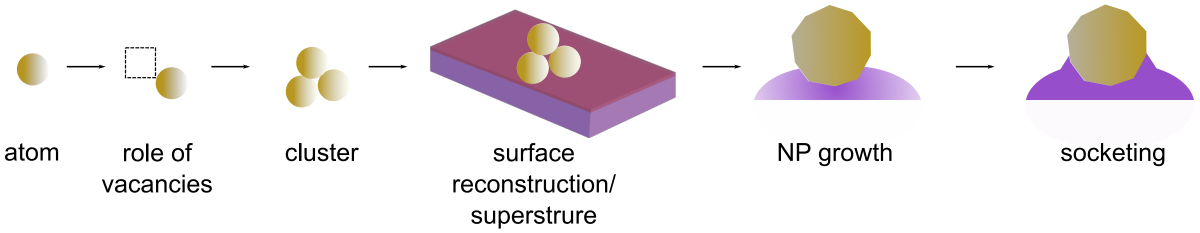

Supplementary Fig. 4. **Schematic representing the whole exsolution mechanism as outlined in this work**. Freely moving dopant atoms over ideal STO-based host surfaces will lock in place when meeting a surface vacancy. These trapped ions will then pair up to initiate cluster nucleation, while the continuous reduction of the host surface at temperature will generate a reconstruction or ordering of defects at the host surface, contributing to initiate the exsolution process. Further exsolved ions cluster growth will follow as the temperature is increased, until the socketing is achieved at higher exsolution temperatures.

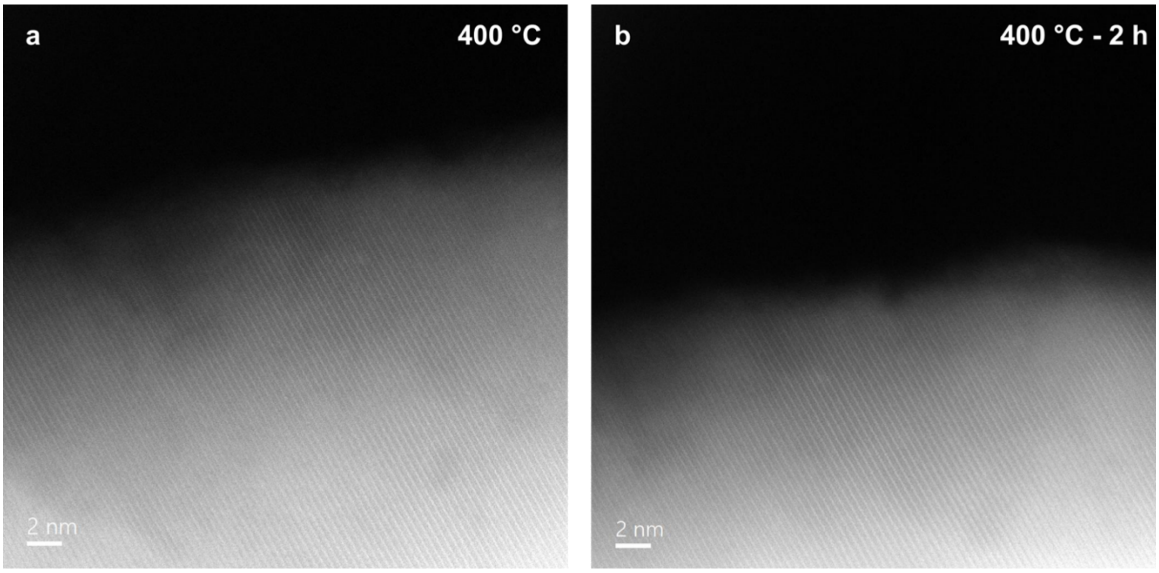

Supplementary Fig. 5. STEM images of the ROI acquired in situ at 400 °C (a) and after a dwell of ~2 hours at 400 °C (b) in UHV. Source data are provided as a Source Data file.

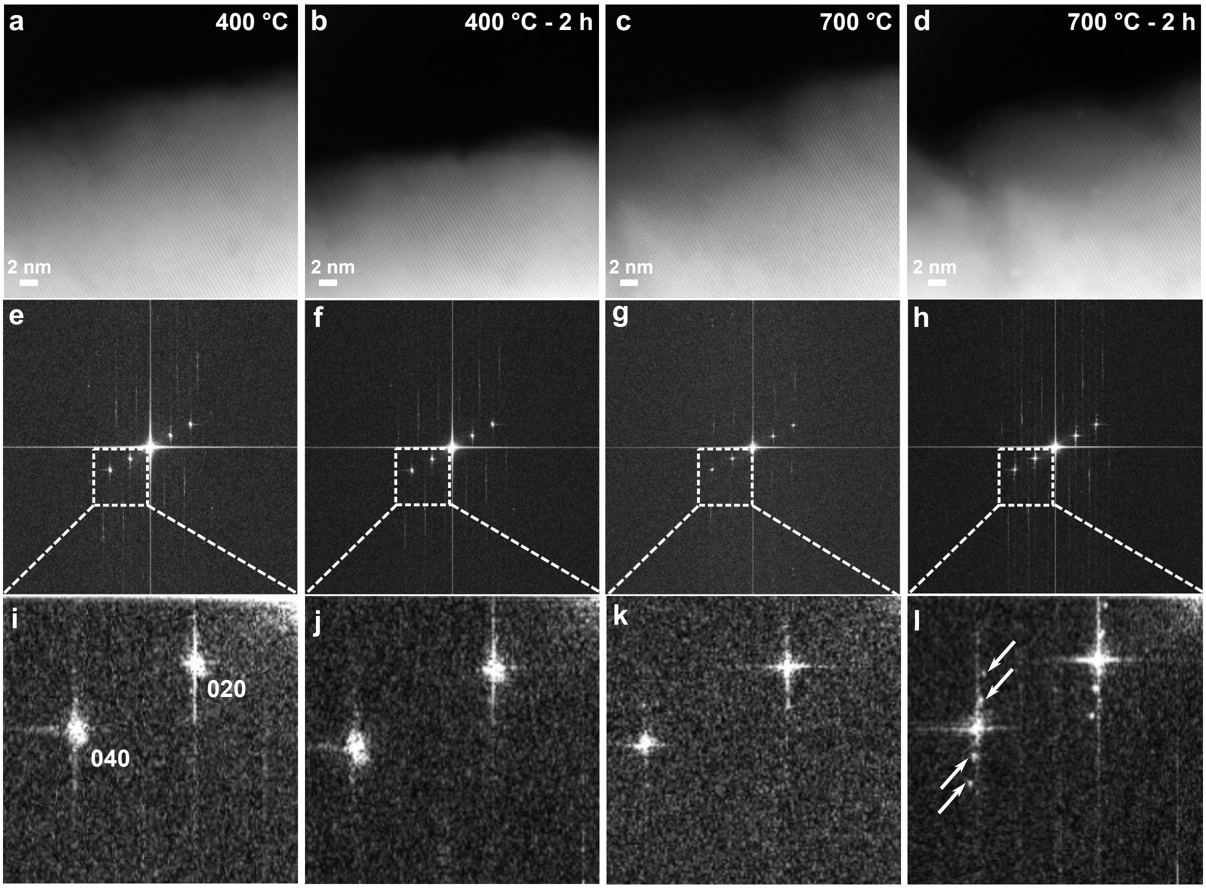

Supplementary Fig. 6. Crystal structure evolution during in situ exsolution studied via FFT analysis at (a,e,i) 400 °C, (b,f,j) after a dwell of 2 h at 400 °C, (c,g,k) 700 °C, and (d,h,l) after a dwell of 2 h at 700 °C. STEM micrographs (a,b,c,d), corresponding generated FFTs with outlined ROIs (e,f,g,h), and magnified ROIs (i,j,k,l) are presented in the top, middle, and bottom rows, respectively. The emergence of extra diffractogram spots in the Fourier transforms (FFTs) of the STEM images is visible starting after a dwell of 2 h at 700 °C (h,l), in addition to the (0 2 0), (0 -2 0), (0 4 0), and (0 -4 0) set of planes of the cubic STO found for the ~[301] Ir-doped STO sample surface at 400 °C (e,i). Source data are provided as a Source Data file.

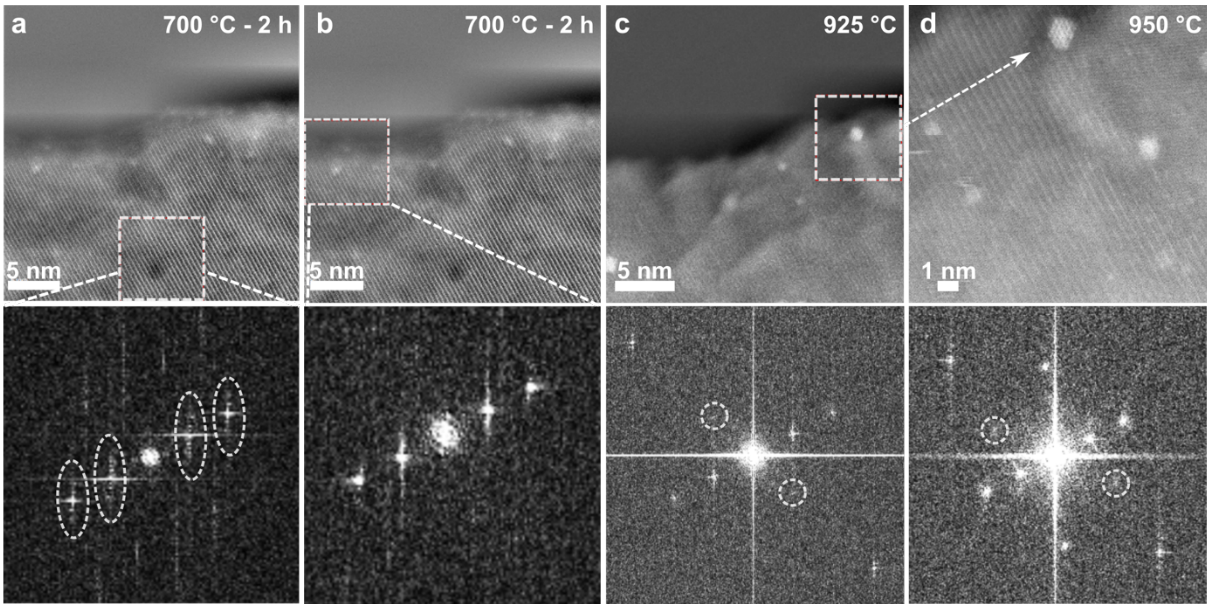

Supplementary Fig. 7. Superstructure analysis. (a) STEM micrograph acquired at 700 °C, with FFT originated from the region boxed in white, showing the superstructure spots discussed in Results section "Host Structure Evolution during In Situ Exsolution", highlighted by the dashed ovals in the bottom panel; (b) Same micrograph as (a) with FFT generated from the region boxed in white in (b), where a nucleating NP is present. (c) STEM micrograph acquired at 925 °C on the same ROI, with FFT showing new signals appearing, circled in white. (d) STEM micrograph acquired at 950 °C on the region boxed in (c), with the FFT generated from the whole image showing the new diffraction signals becoming clearer. The new signals circled in (c) and (d) are indeed generated from the faceted NP at the top of the image. All STEM micrographs have been background subtracted. Source data are provided as a Source Data file.

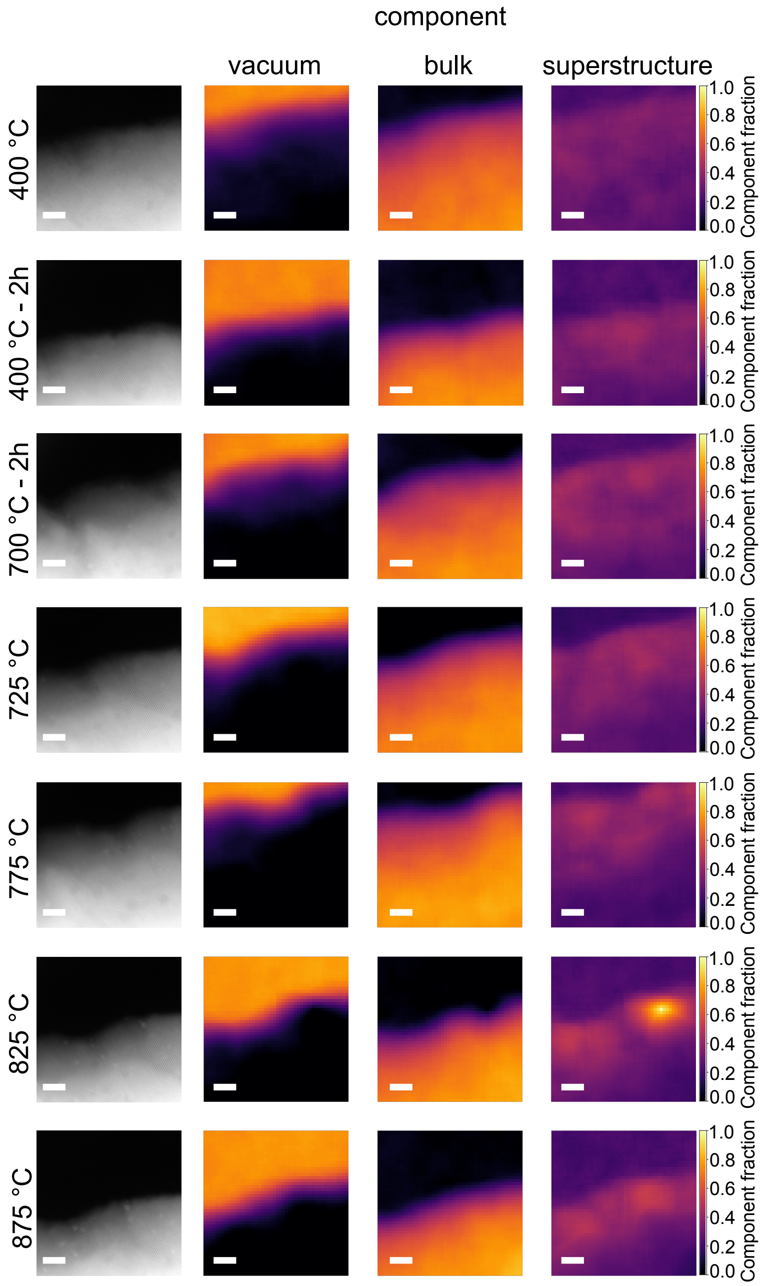

Supplementary Fig. 8. Map progression obtained via the N-FINDR analysis for the images acquired from 400-900 °C which are not included in Fig. 4, with the three components showing the abundance relative to the vacuum, the abundance relative to the bulk, and the abundance relative to the superstructure, from left to right, respectively. Scalebars in all images are 5 nm, whereas the colour scales for the vacuum, bulk, and superstructure components show each component fraction and refer to the intensity of the component at each pixel. Source data are provided as a Source Data file.

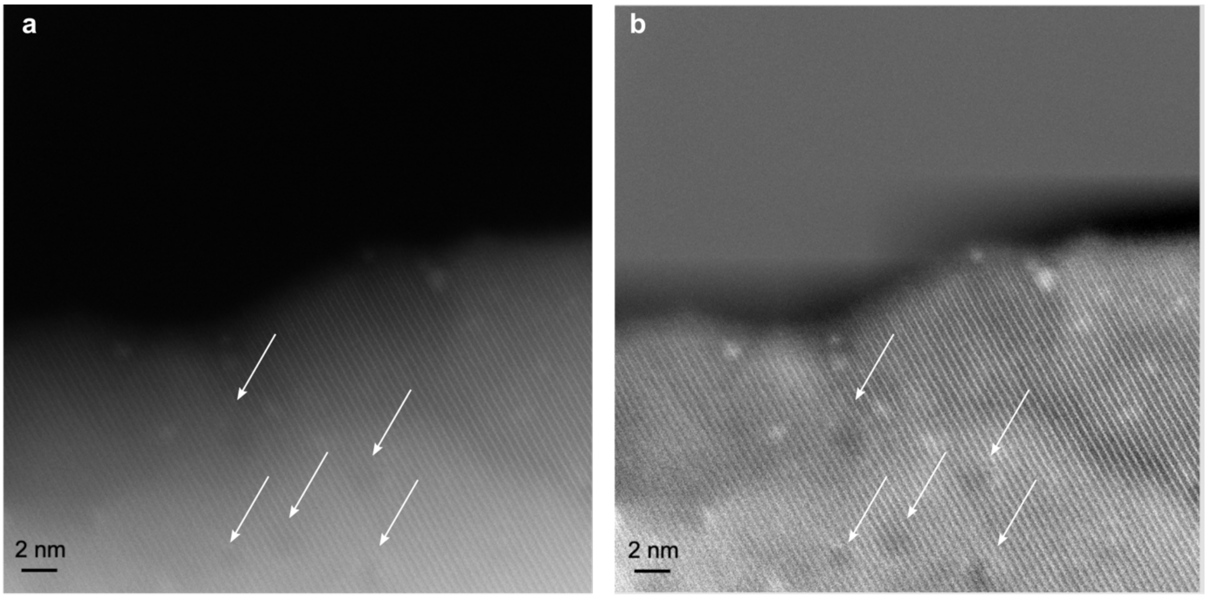

Supplementary Fig. 9. Background subtraction script applied to subtract the slowly varying background by successive reduction and linear interpolation scaling. (a) Raw STEM micrograph, before correction; (b) STEM micrograph after applying the background subtraction algorithm to the raw image in (a). The arrows in (a) and (b) highlight the easier visual observation of the dark-contrast features discussed in the Results section "Host Structure Evolution during In Situ Exsolution" before and after background subtraction. Source data are provided as a Source Data file.

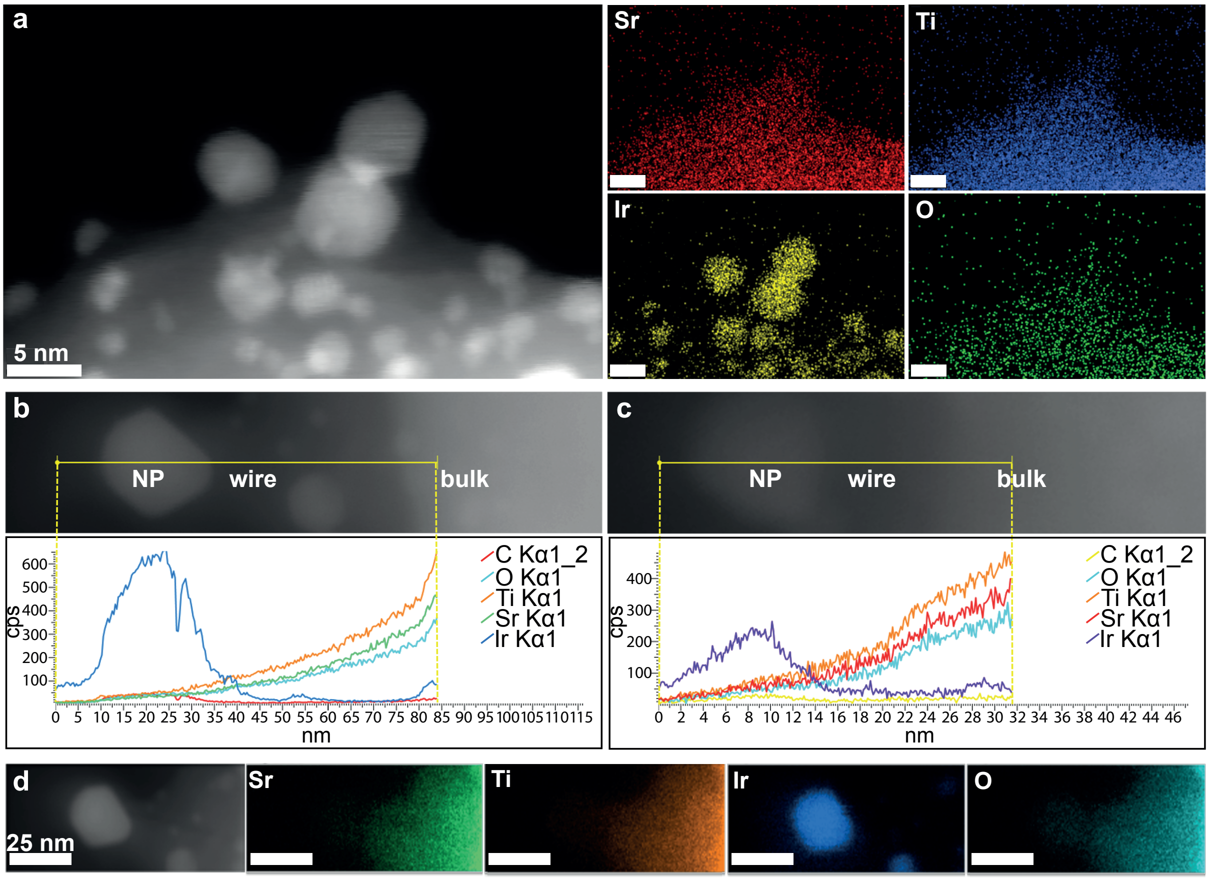

Supplementary Fig. 10. Elemental analysis of pyramidal sockets. (a) STEM-EDX analysis of the 0.5% Ir-STO sample reduced *in situ* during a heating experiment. The elemental maps of Sr, Ti, Ir, and O confirm the pyramidal socket supporting the three exsolved Ir NPs presents the STO composition. (b, c) Linescan EDX analyses of two sockets grown from an exsolved

5% Ir-doped STO sample, showing resemblance of the socket with nanowires. (d) Elemental maps of the same region reported in (b) showing compositional identity between the nanowire and the bulk, with Sr, Ti, and O found for both regions. Scalebars are 5 nm for all panels in (a) and 25 nm for all panels in (d). Source data are provided as a Source Data file.

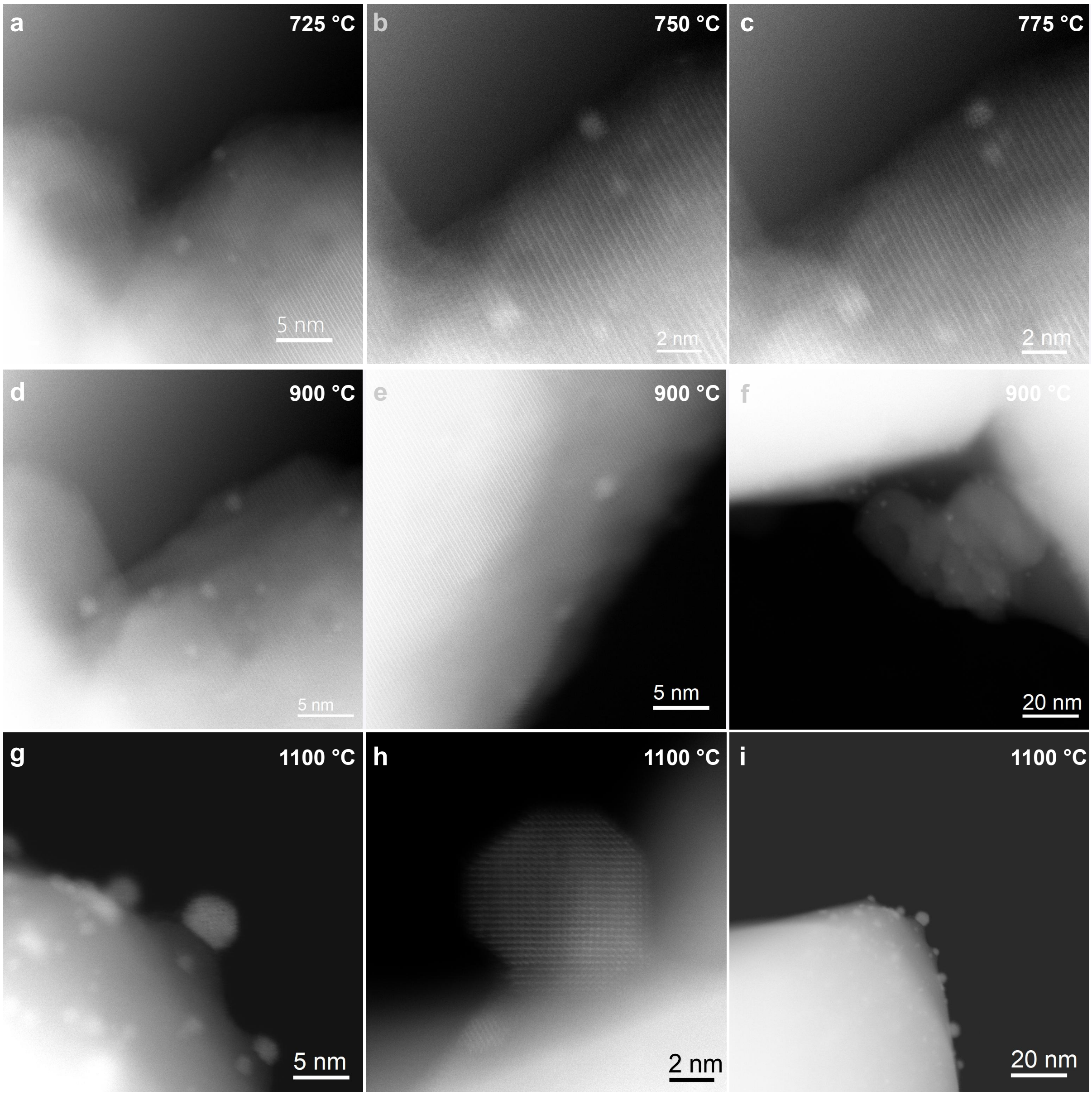

Supplementary Fig. 11. **STEM images acquired at target temperatures on control areas not irradiated by the electron beam** during the in situ experiments at (a) 725 °C, (b) 750 °C, (c) 775 °C, (d) as reached 900 °C, (e) after ~10 minutes at 900 °C, (f) after ~30 minutes at 900 °C, and (g-i) at room temperature after having quenched the sample from 1100 °C *in situ*. The different areas captured in the images show comparable behaviour of cluster diffusion and movement, as visible throughout the evolution observed from (a-c), NP exsolution with comparable sizes obtained at the same temperature over different areas (d-f), as well as socketing evolution and structure (g-i). NOTE: the image in (b) was acquired before the images reported in Fig. 3 (a-b). Source data are provided as a Source Data file.

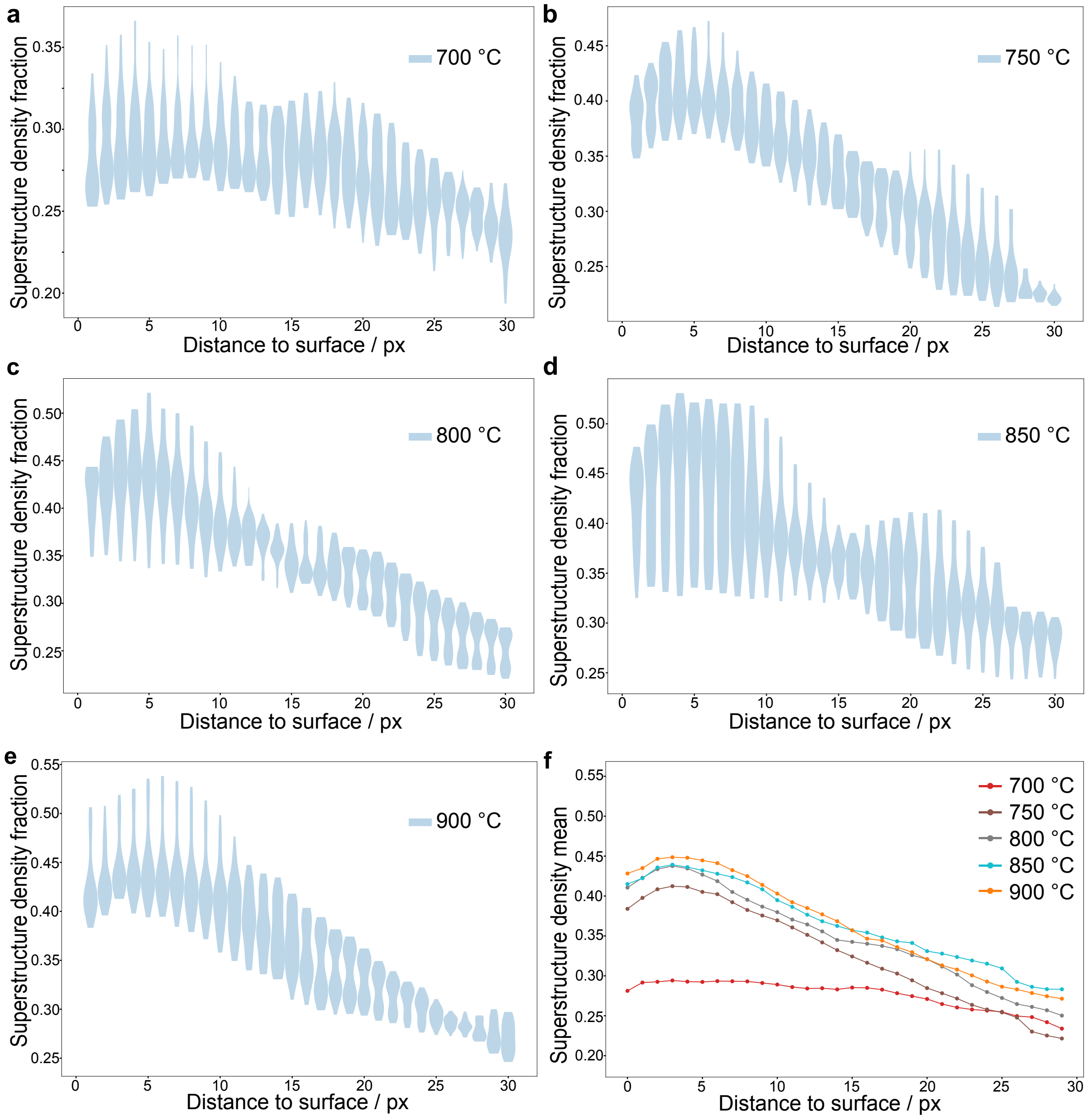

Supplementary Fig. 12. **Individual violin plots of the superstructure component density as a function of distance from the surface of the monitored grain**, plotted in a single graph in Figure 4f, for the targeted temperature (a) 700, (b) 750, (c) 800, (d) 850, and (e) 900 °C. Original image is 1024 pixels across (i.e., 34.4 nm) and pixel size ~ 0.72 nm; (f) plot of the mean values for each temperature point plotted in (a-e). Source data are provided as a Source Data file.

Supplementary Note 2. Additional discussion on the link between exsolution and surface reconstruction

Several models have been proposed for surface reconstructions on various orientations of STO, with structural distortions and re-arrangements extending from the surface to the sub-surface in UHV-high-temperature conditions.[10–12] These are assumed to play a role in facilitating B-site dopant diffusion under exsolution conditions, due to the formation energy of V_O being lower at the surface,[13] but this had not yet been demonstrated *in situ*. The machine-learning analysis presented in Figure 4 of the main text suggests a gradient of defects (*e.g.*, V_O) and/or of cations (*e.g.*, Ir) enriching the surface as the temperature is increased during exsolution, which results in a degree of local ordering. Modelling on the role of V_O in the exsolution process suggests that metal segregation is stabilized by the introduction of oxygen vacancies, facilitating NP nucleation at the host surface.[14,15] As the sample is heated *in situ*, the increasing reducing conditions with temperature and time cause further oxygen vacancies to be

generated in the system (V_O generation typically reported above 600 °C for undoped $SrTiO_3$[16–18]). At higher concentration, and decreasing the pO_2 and increasing the temperature, point defects are known to accumulate in clusters and sometimes order.[19] Indeed, the ordering of oxygen vacancies has been shown to give rise to superstructures, and several surface reconstructions have previously been observed for $SrTiO_3$-based materials at T≥600 °C in UHV.[20,21] By directly probing the evolution of such structures, our evidence confirms the link between exsolution and surface reconstructions/ordering, allowing the identification of the first step involved in the exsolution mechanism for this Ir-STO system.

Supplementary References

1. Kresse, G. & Furthmüller, J. Efficiency of ab-initio total energy calculations for metals and semiconductors using a plane-wave basis set. *Comput. Mater. Sci.* **6**, 15–50 (1996).
2. Kresse, G. & Furthmüller, J. Efficient iterative schemes for ab initio total-energy calculations using a plane-wave basis set. *Phys. Rev. B* **54**, 11169–11186 (1996).
3. Kresse, G. & Hafner, J. Ab initio molecular dynamics for liquid metals. *Phys. Rev. B* **47**, 558–561 (1993).
4. Kresse, G. & Hafner, J. Ab initio molecular-dynamics simulation of the liquid-metal--amorphous-semiconductor transition in germanium. *Phys. Rev. B* **49**, 14251–14269 (1994).
5. Dovesi, R. *et al.* Quantum-mechanical condensed matter simulations with CRYSTAL. *WIREs Comput. Mol. Sci.* **8**, e1360 (2018).
6. Dovesi, R. *et al.* CRYSTAL17. User's Manual. *CRYSTAL17 User's Man.* **211** (2018). http://tutorials.crystalsolutions.eu/.
7. Wood, N. D. *et al.* An atomistic modelling investigation of the defect chemistry of $SrTiO_3$ and its Ruddlesden-Popper phases, $Sr_{n+1}Ti_nO_{3n+1}$ (n = 1–3). *J. Solid State Chem.* **303**, 122523 (2021).
8. Gale, J. D. & Rohl, A. L. The general utility lattice program (GULP). *Mol. Simul.* **29**, 291–341 (2003).
9. Krivanek, O. L. *et al.* Atom-by-atom structural and chemical analysis by annular dark-field electron microscopy. *Nature* **464**, 571–574 (2010).
10. Herger, R. *et al.* Surface of strontium titanate. *Phys. Rev. Lett.* **98**, 076102 (2007).
11. Kienzle, D. M., Becerra-Toledo, A. E. & Marks, L. D. Vacant-site octahedral tilings on SrTiO(3) (001), the (sqrt[13]×sqrt[13])R33.7° surface, and related structures. *Phys. Rev. Lett.* **106**, 176102 (2011).

12. Kubo, T., Orita, H. & Nozoye, H. Atomic structures of the defective SrTiO3 (001) surface. *Phys. Chem. Chem. Phys.* **13**, 16516–16519 (2011).

13. Carrasco, J., Lopez, N., Illas, F. & Freund, H.-J. Bulk and surface oxygen vacancy formation and diffusion in single crystals, ultrathin films, and metal grown oxide structures. *J. Chem. Phys.* **125**, 074711 (2006).

14. Hamada, I., Uozumi, A., Morikawa, Y., Yanase, A. & Katayama-Yoshida, H. A density functional theory study of self-regenerating catalysts $LaFe_{1-x}M_xO_{3-y}$ (M = Pd, Rh, Pt). *J. Am. Chem. Soc.* **133**, 18506–18509 (2011).

15. Tian, Z. *et al.* First-principles investigation on the segregation of Pd at $LaFe_{1-x}Pd_xO_{3-y}$ surfaces. *Nanoscale Res. Lett.* **8**, 203 (2013).

16. Zhu, J. *et al.* Probing vacancy behavior across complex oxide heterointerfaces. *Sci. Adv.* **5**, eaau8467 (2019).

17. Yao, L., Inkinen, S. & van Dijken, S. Direct observation of oxygen vacancy-driven structural and resistive phase transitions in $La_{2/3}Sr_{1/3}MnO_3$. *Nat. Commun.* **8**, 14544 (2017).

18. Perez-Casero, R. *et al.* Thin films of oxygen-deficient perovskite phases by pulsed-laser ablation of strontium titanate. *Phys. Rev. B* **75**, 165317 (2007).

19. Hertkorn, D. *et al.* Morphology and oxygen vacancy investigation of strontium titanate-based photo electrochemical cells. *J. Mater. Sci.* **50**, (2014).

20. Shimizu, R. *et al.* Effect of oxygen deficiency on $SrTiO_3$(001) surface reconstructions. *Appl. Phys. Lett.* **100**, 263106 (2012).

21. Kousi, K., Tang, C., Metcalfe, I. S. & Neagu, D. Emergence and future of exsolved materials. *Small* **17**, 2006479.
