# Supplementary material for: Real-time insight into the multistage mechanism of nanoparticle exsolution from a perovskite host surface
Source: Nat Commun. 2023 Mar 29;14:1754. doi: 10.1038/s41467-023-37212-6 (PMC10060596; doi:10.1038/s41467-023-37212-6)
Supplement: Supplementary file 2 — Description of Additional Supplementary Information [file 41467_2023_37212_MOESM2_ESM.pdf]

Description of Additional Supplementary Information

Title: Supplementary Movie 1

Description: Shows the process described in Figure 3, where the movement of Ir atomic clusters on the host surface further coalescing to form a bigger cluster, and of a nanoparticle traveling along the surface until reaching a stepped surface are observed in situ at 825 °C – 875 °C. The video is shown at 50 times the actual speed.

Title: Supplementary Movie 2:

Description: Supplementary Movie 2 shows the socket evolution presented in Figure 6 acquired in situ from 975 °C to 1100 °C. The video is shown at 50 times the actual speed.”
