# Supplementary figures and images for: Real-time insight into the multistage mechanism of nanoparticle exsolution from a perovskite host surface
Source: Nat Commun. 2023 Mar 29;14:1754. doi: 10.1038/s41467-023-37212-6 (PMC10060596; doi:10.1038/s41467-023-37212-6)

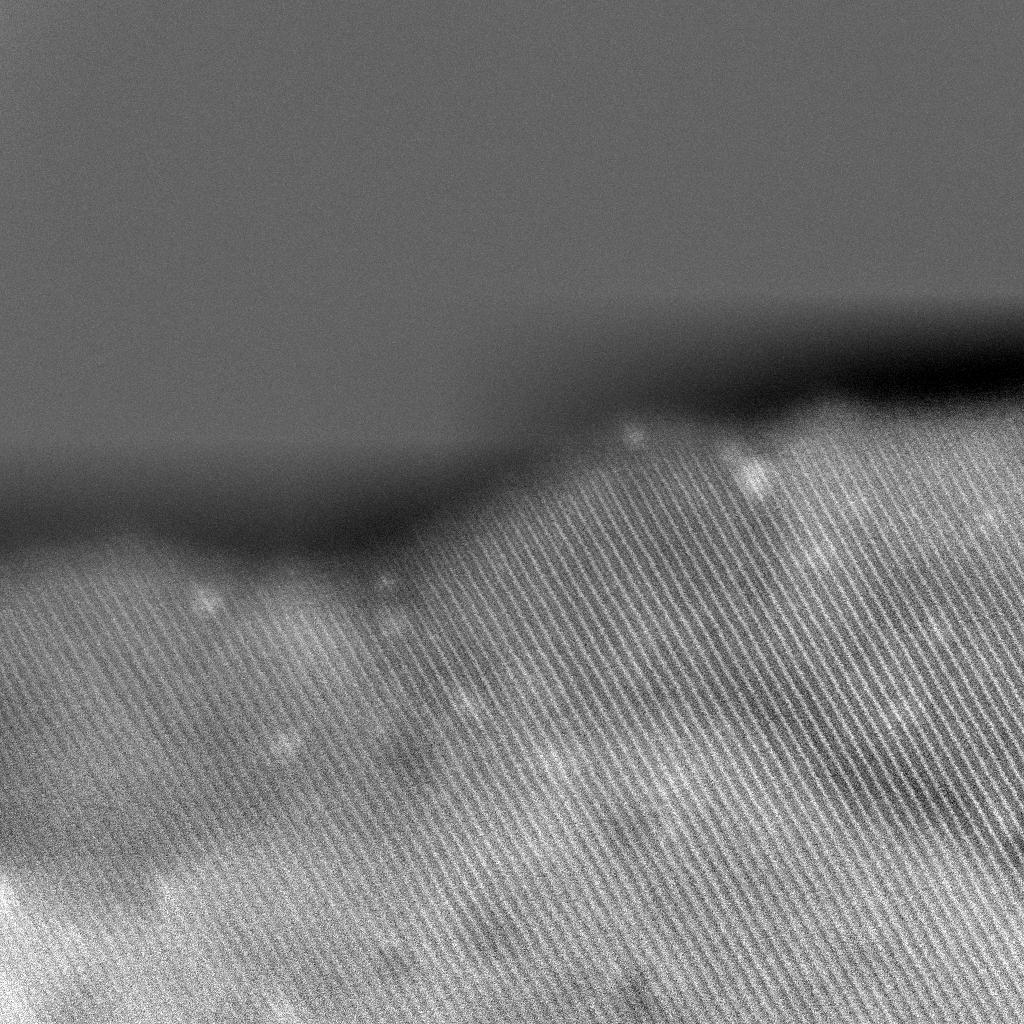

Supplement: Supplementary file 6 — Source Data [file 41467_2023_37212_MOESM6_ESM.zip › Raw_data/Figure5/825C_SuperScan-HAADF-35_2019-06-16T002525.717729_1024x1024_6 (low freq. bkgnd. removed).jpg]

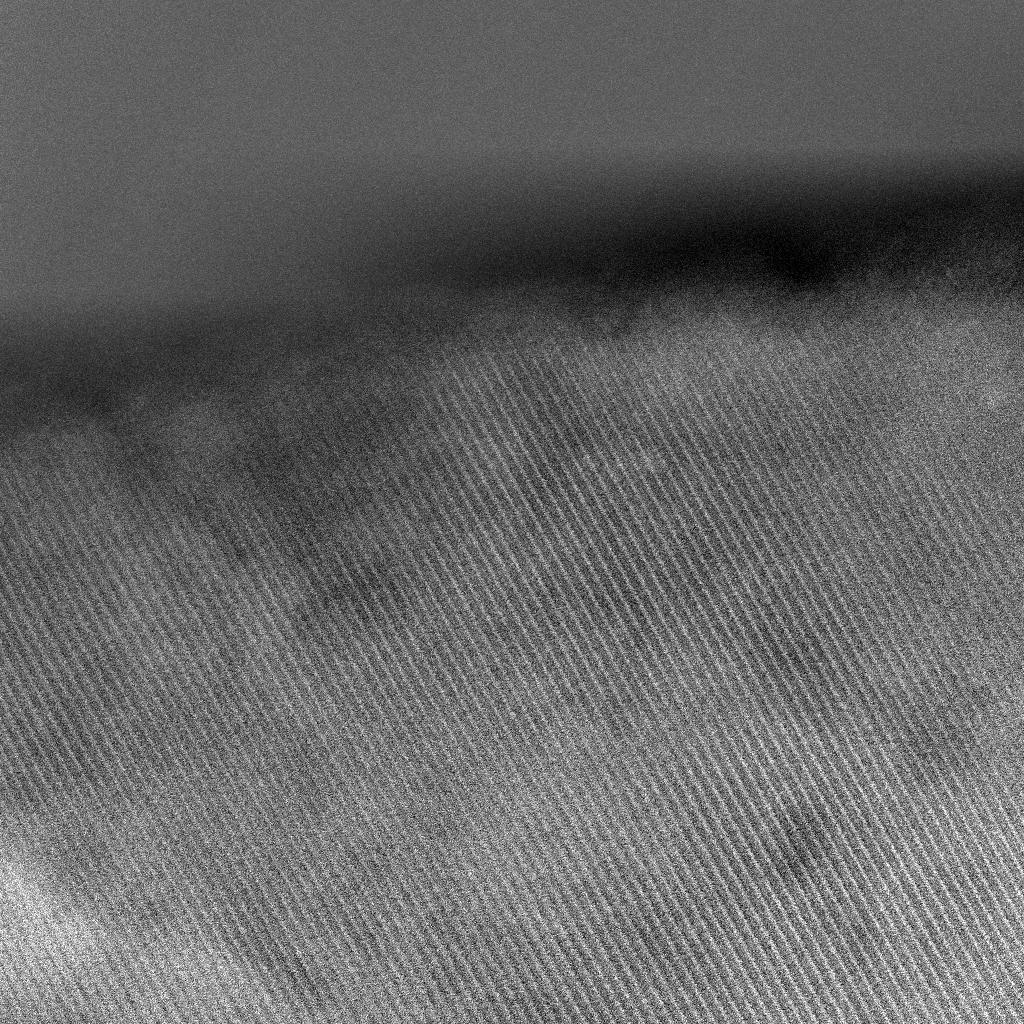

Supplement: Supplementary file 6 — Source Data [file 41467_2023_37212_MOESM6_ESM.zip › Raw_data/Figure5/400C_SuperScan-HAADF-11_2019-06-15T160248.822481_1024x1024_22 (low freq. bkgnd. removed).jpg]

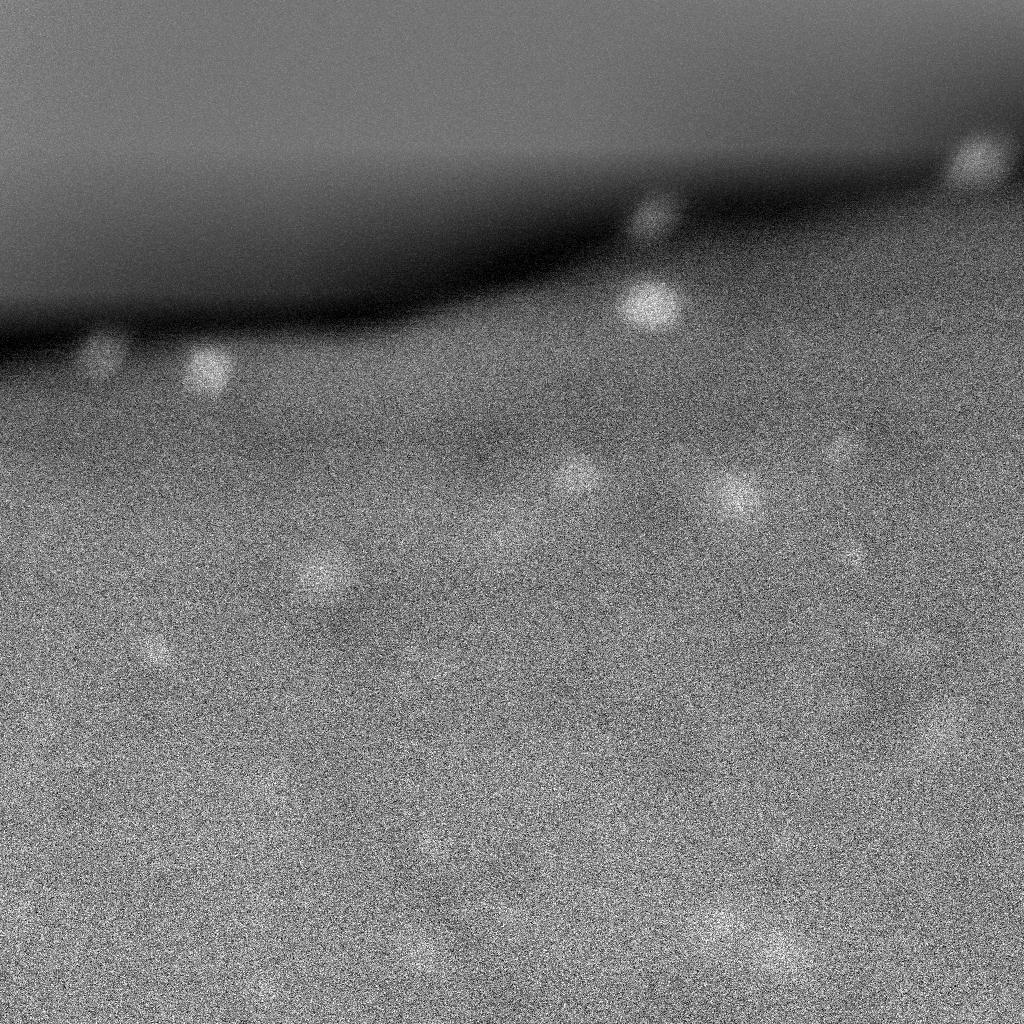

Supplement: Supplementary file 6 — Source Data [file 41467_2023_37212_MOESM6_ESM.zip › Raw_data/Figure5/1000C_SuperScan-HAADF-58_2019-06-16T204308.529478_1024x1024_48 (low freq. bkgnd. removed).jpg]

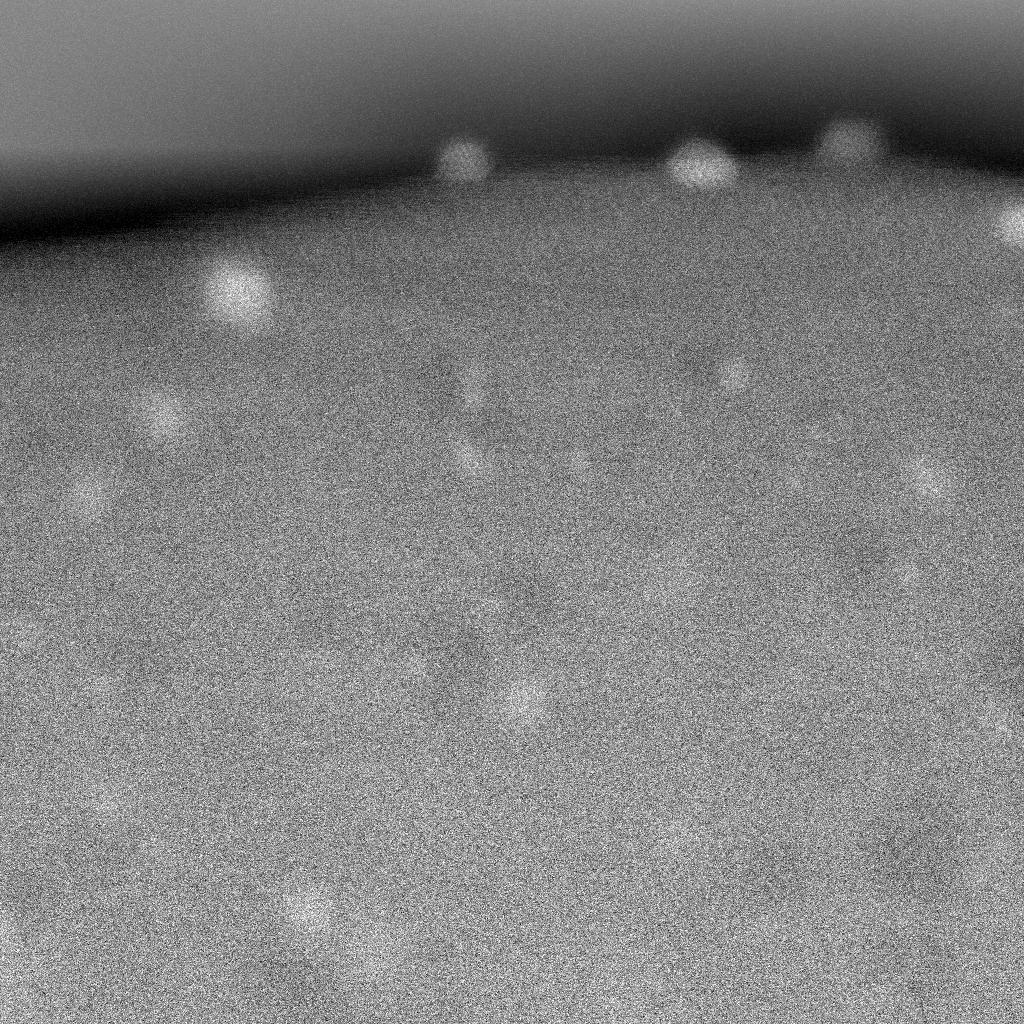

Supplement: Supplementary file 6 — Source Data [file 41467_2023_37212_MOESM6_ESM.zip › Raw_data/Figure5/1050C_SuperScan-HAADF-85_2019-06-16T212840.303726_1024x1024_21 (low freq. bkgnd. removed).jpg]

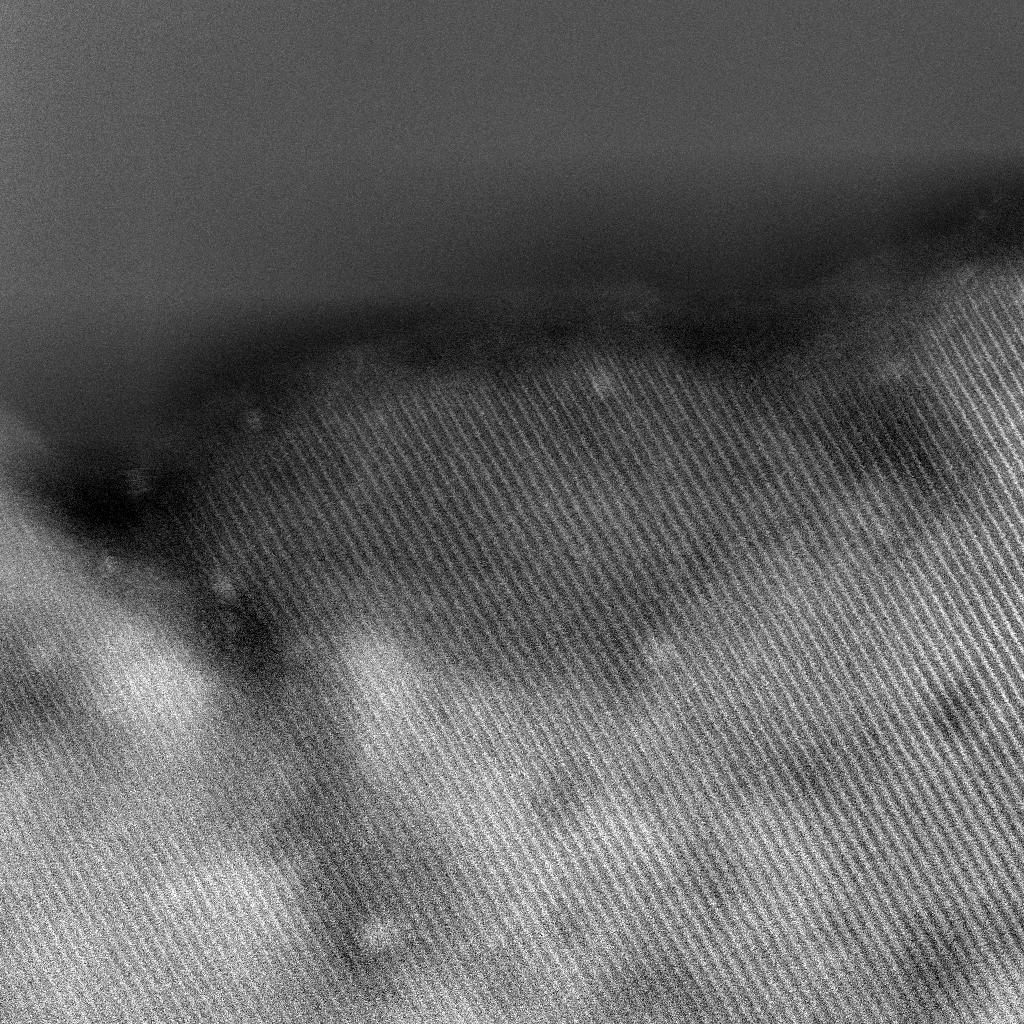

Supplement: Supplementary file 6 — Source Data [file 41467_2023_37212_MOESM6_ESM.zip › Raw_data/Figure5/2h_700C_SuperScan-HAADF-18_2019-06-15T201905.332339_1024x1024_5 (low freq. bkgnd. removed).jpg]

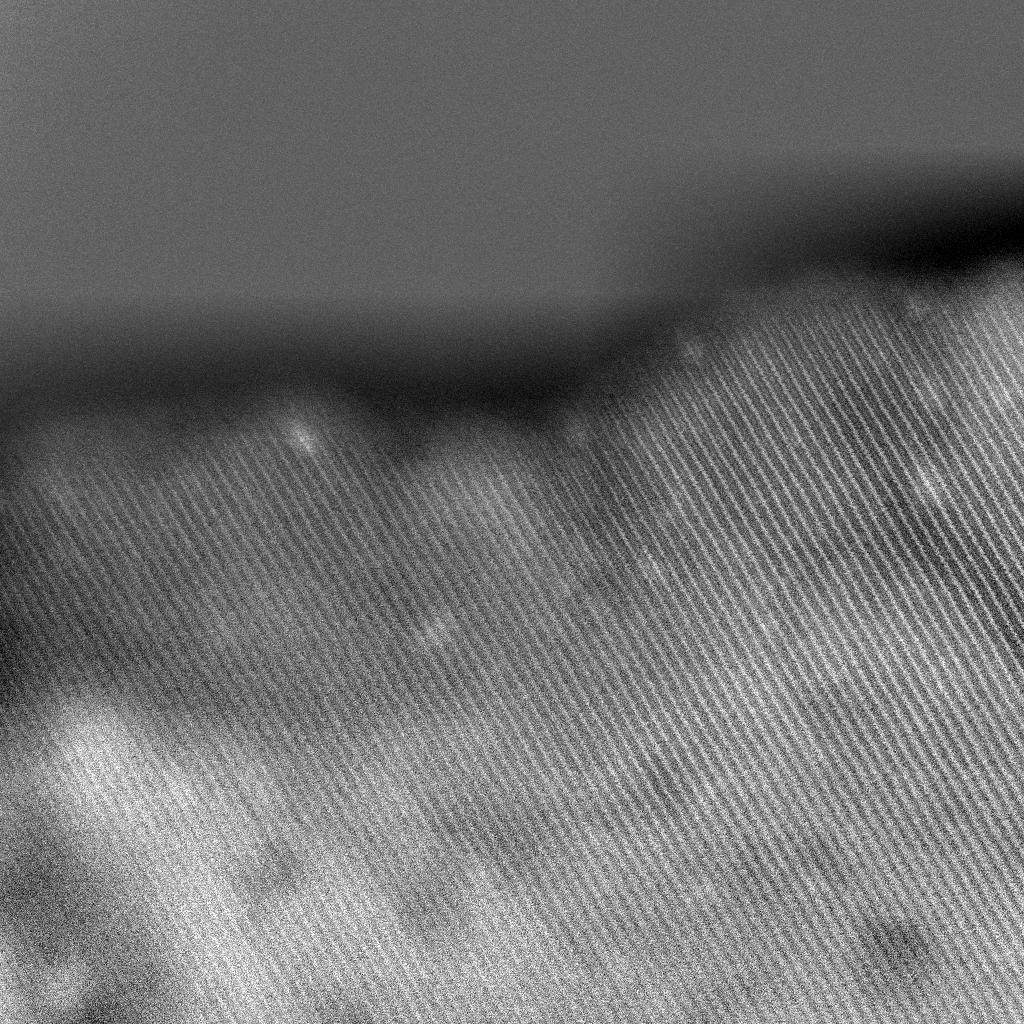

Supplement: Supplementary file 6 — Source Data [file 41467_2023_37212_MOESM6_ESM.zip › Raw_data/Figure5/800C_SuperScan-HAADF-26_2019-06-15T232948.693862_1024x1024_6 (low freq. bkgnd. removed).jpg]

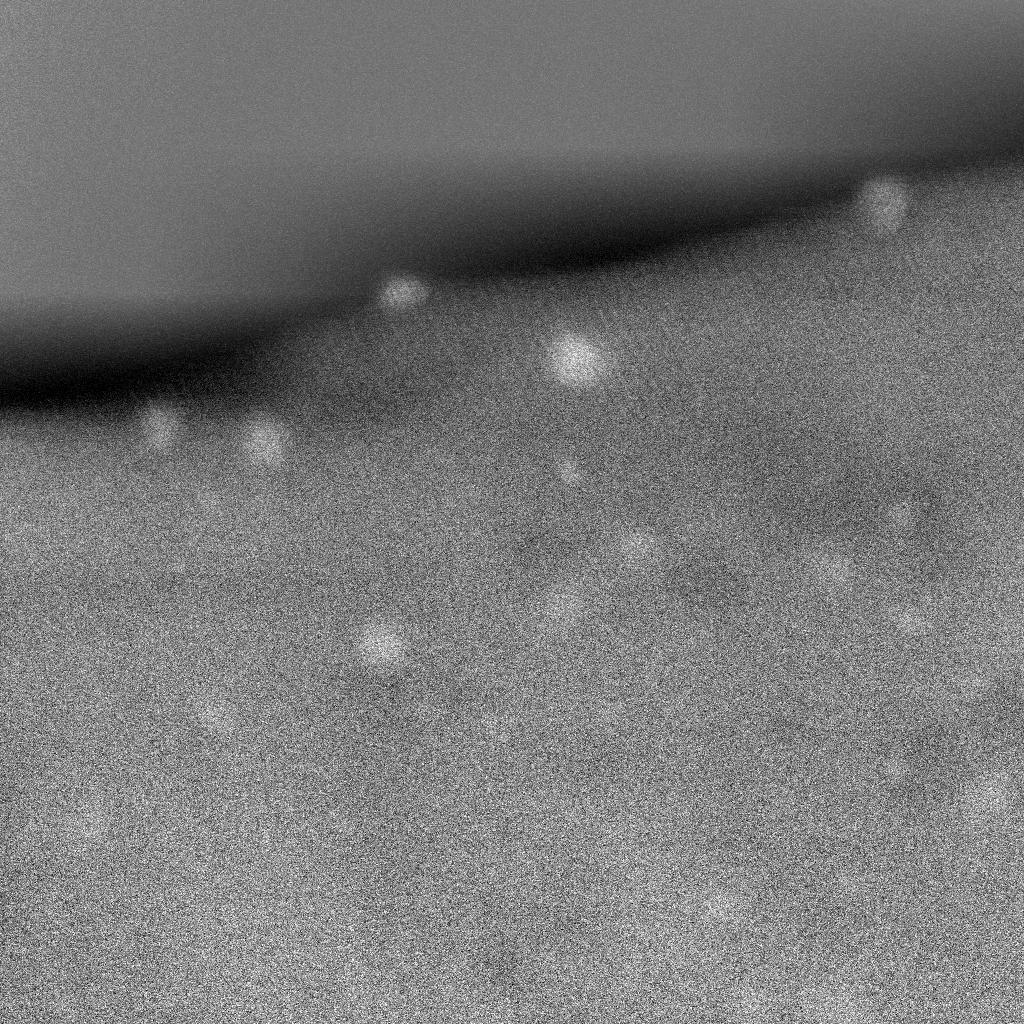

Supplement: Supplementary file 6 — Source Data [file 41467_2023_37212_MOESM6_ESM.zip › Raw_data/Figure5/975C_SuperScan-HAADF-29_2019-06-16T200111.368504_1024x1024_82 (low freq. bkgnd. removed).jpg]

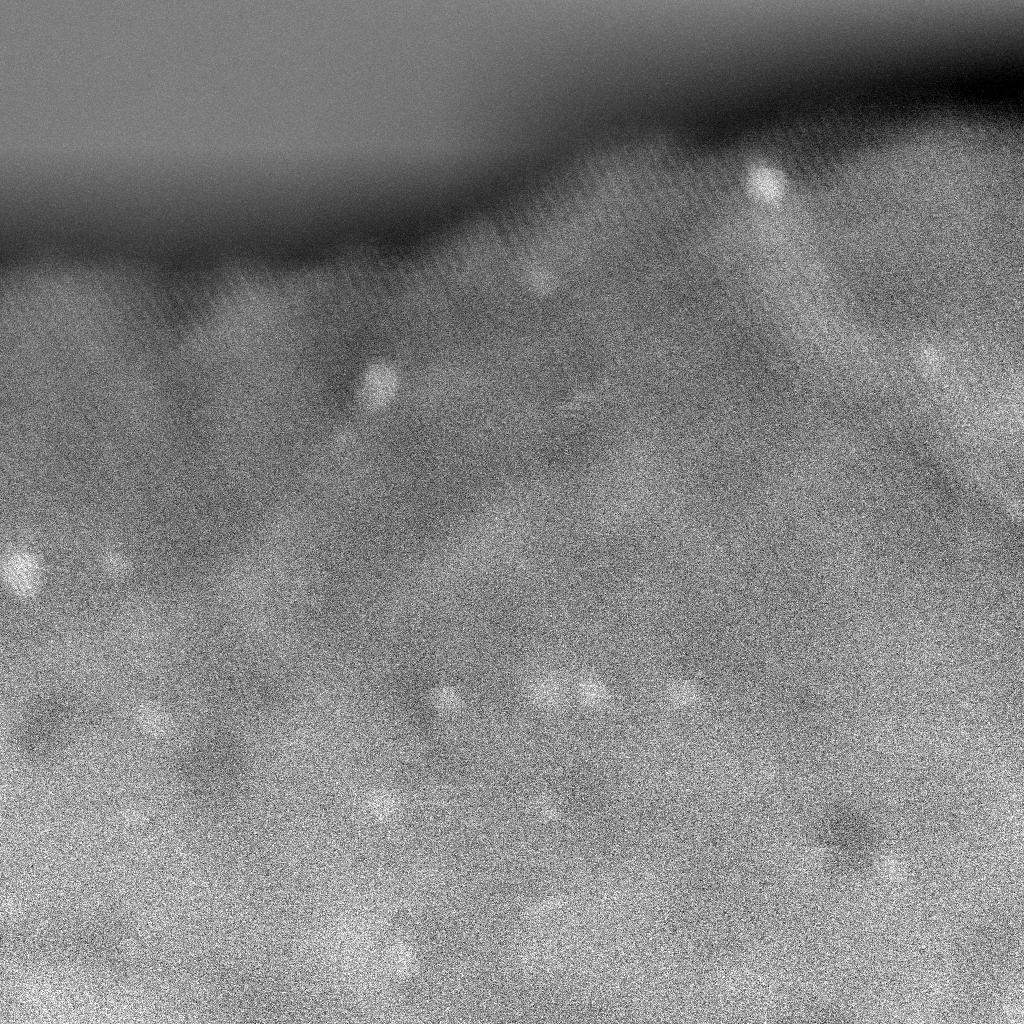

Supplement: Supplementary file 6 — Source Data [file 41467_2023_37212_MOESM6_ESM.zip › Raw_data/Figure5/925C_SuperScan-HAADF-54_2019-06-16T175802.371878_1024x1024_133 (low freq. bkgnd. removed).jpg]

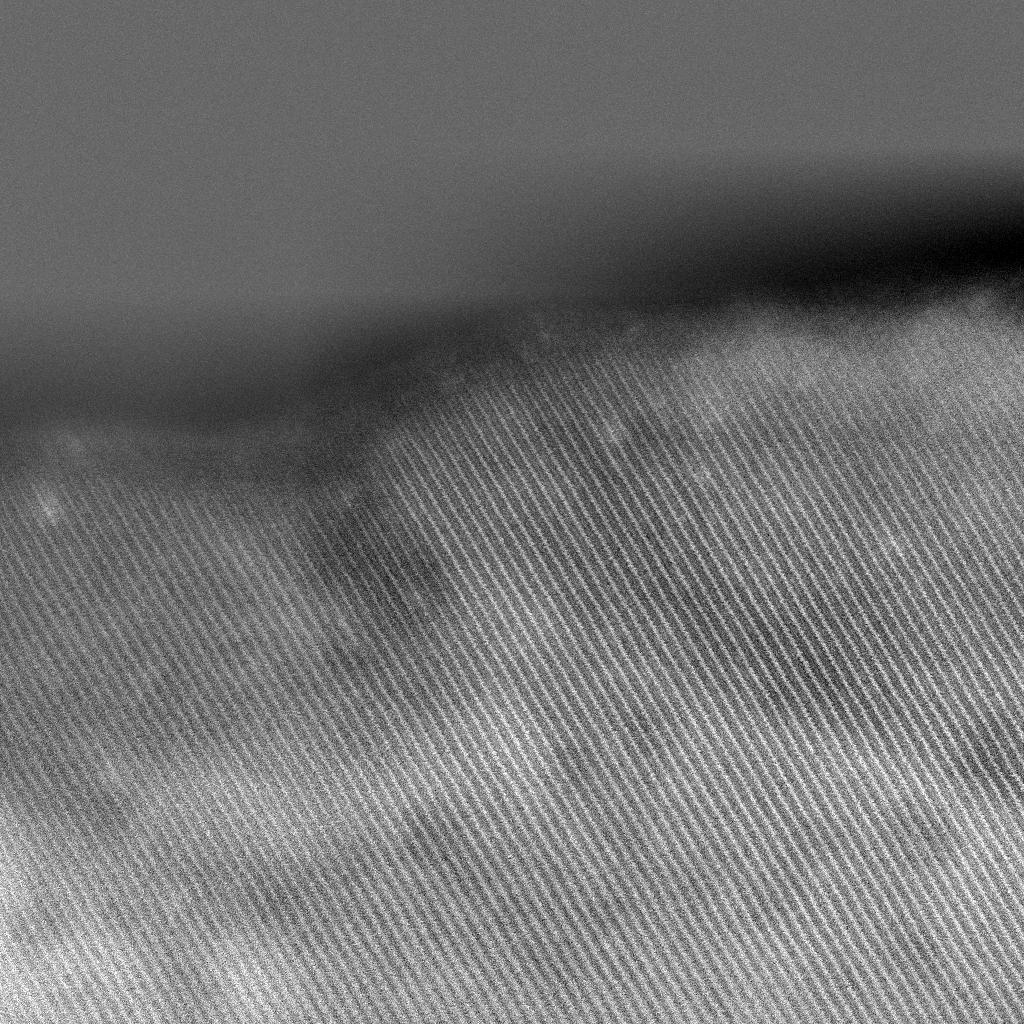

Supplement: Supplementary file 6 — Source Data [file 41467_2023_37212_MOESM6_ESM.zip › Raw_data/Figure5/725C_SuperScan-HAADF-24_2019-06-15T205655.766201_1024x1024_13 (low freq. bkgnd. removed).jpg]

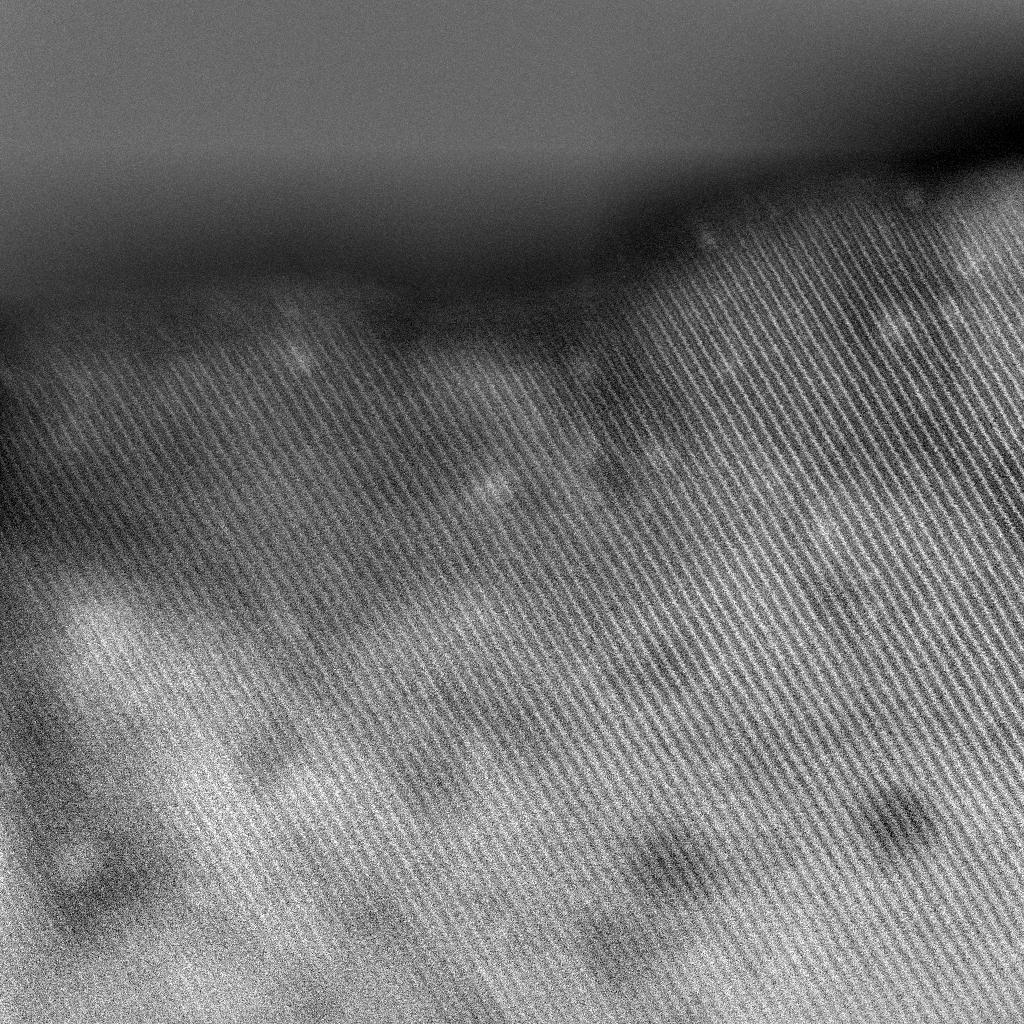

Supplement: Supplementary file 6 — Source Data [file 41467_2023_37212_MOESM6_ESM.zip › Raw_data/Figure5/775C_SuperScan-HAADF-15_2019-06-15T223843.546546_1024x1024_8 (low freq. bkgnd. removed).jpg]

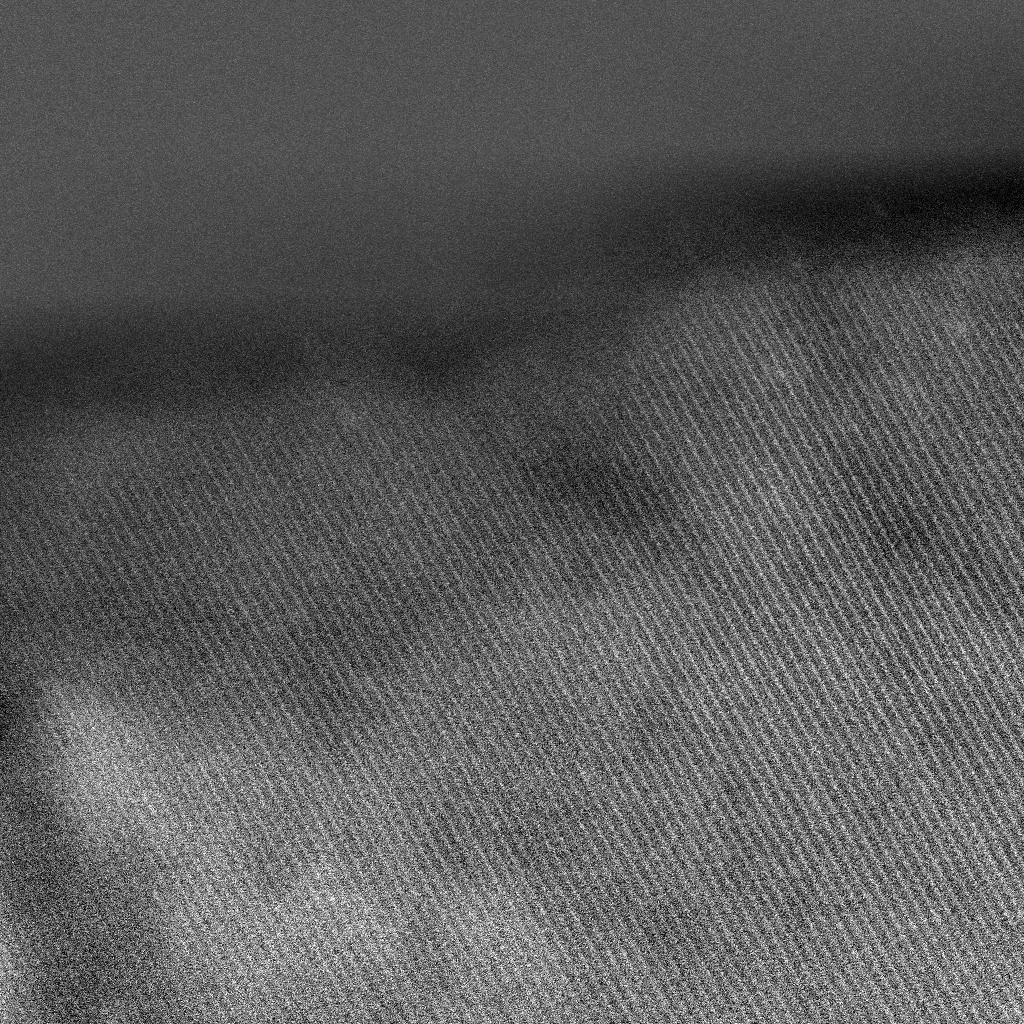

Supplement: Supplementary file 6 — Source Data [file 41467_2023_37212_MOESM6_ESM.zip › Raw_data/Figure5/700C_Super700C_Scan-HAADF-13_2019-06-15T181636.148990_1024x1024_10_NOsb (low freq. bkgnd. removed).jpg]

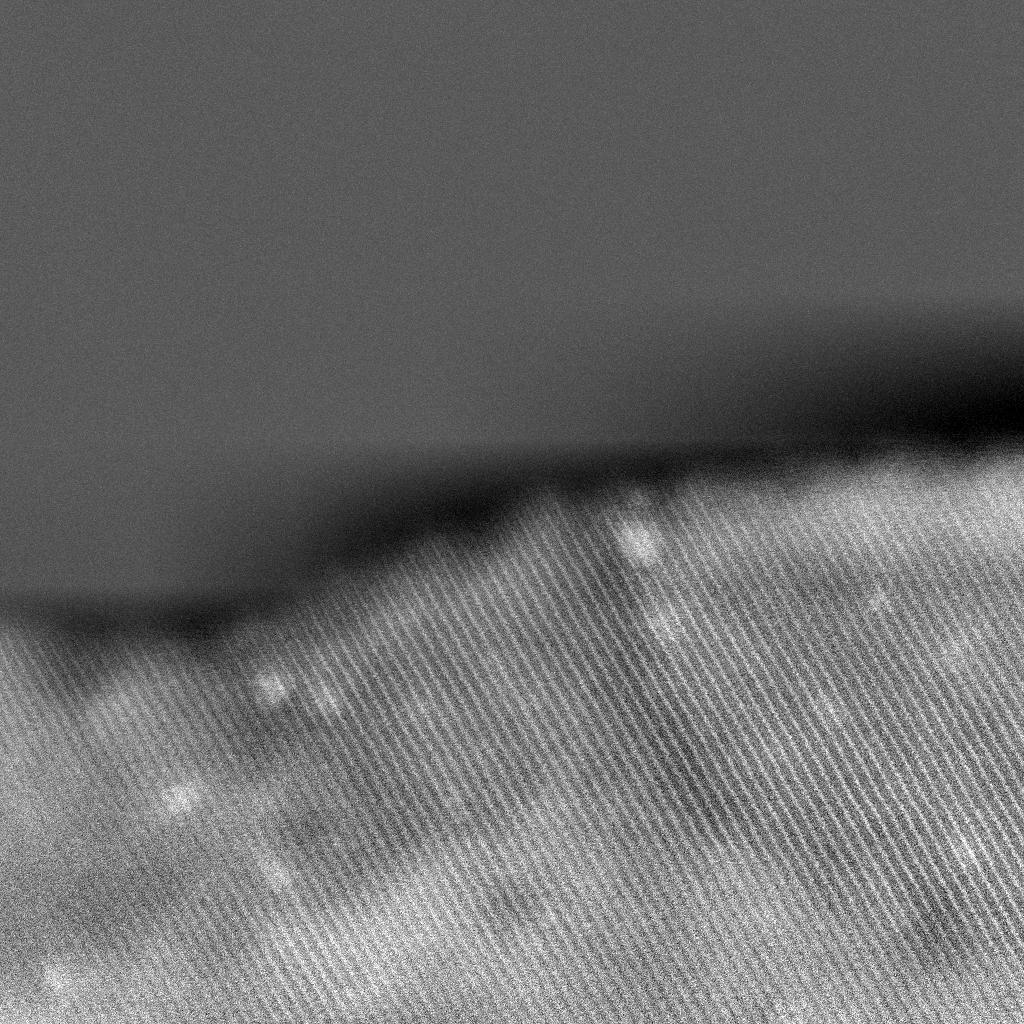

Supplement: Supplementary file 6 — Source Data [file 41467_2023_37212_MOESM6_ESM.zip › Raw_data/Figure5/875C_SuperScan-HAADF-48_2019-06-16T013757.464635_1024x1024_13 (low freq. bkgnd. removed).jpg]

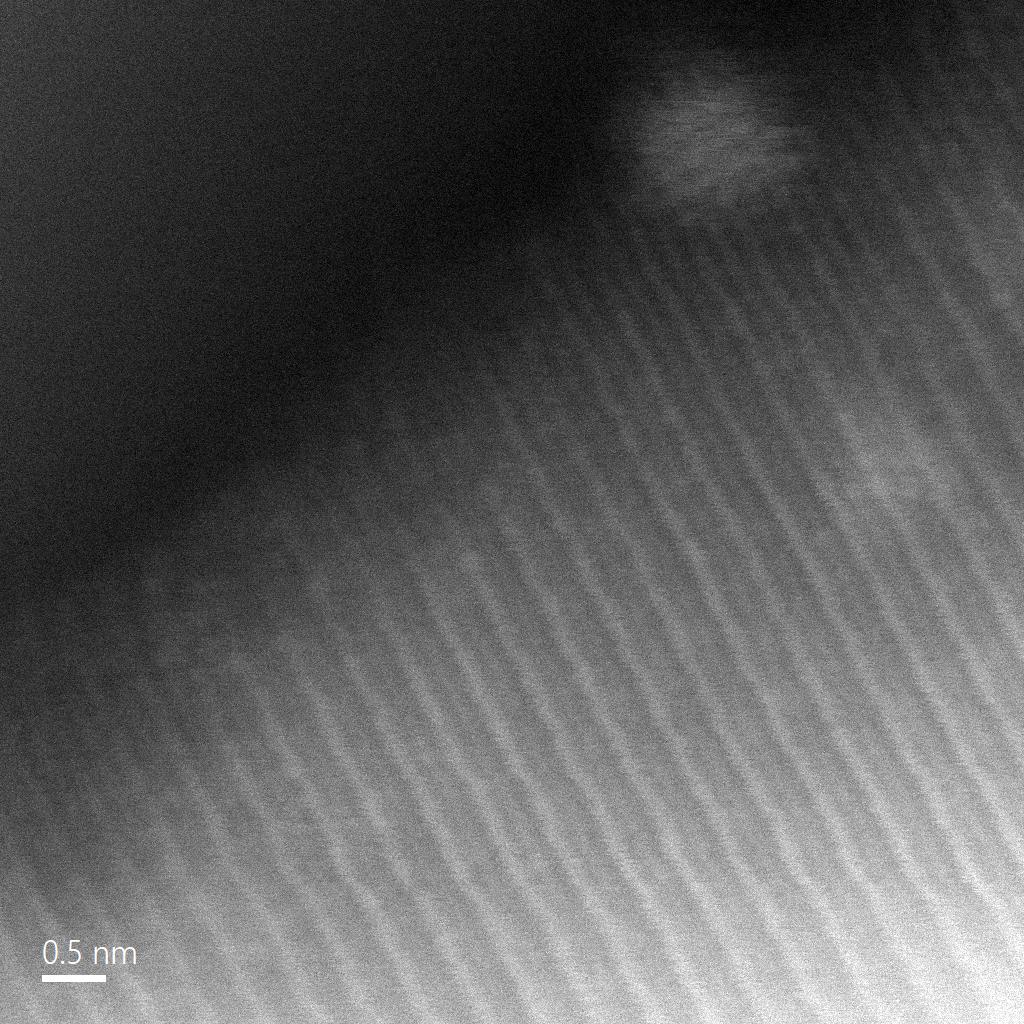

Supplement: Supplementary file 6 — Source Data [file 41467_2023_37212_MOESM6_ESM.zip › Raw_data/Figure2/Fig2i_SuperScan-HAADF-7_2019-06-15T215122.075023_1024x1024_1_scalebar.jpg]

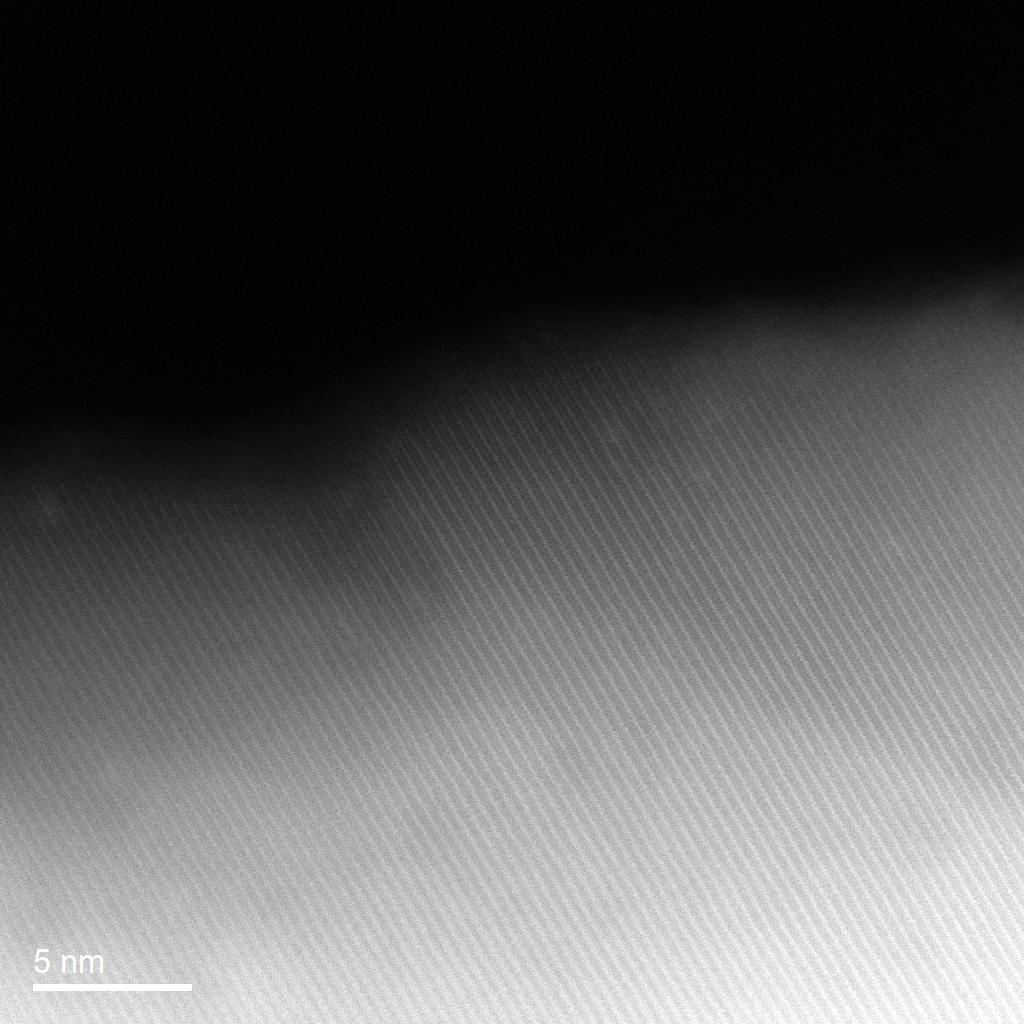

Supplement: Supplementary file 6 — Source Data [file 41467_2023_37212_MOESM6_ESM.zip › Raw_data/Figure2/Fig2e_725_SuperScan-HAADF-24_2019-06-15T205655.766201_1024x1024_13_scalebar.jpg]

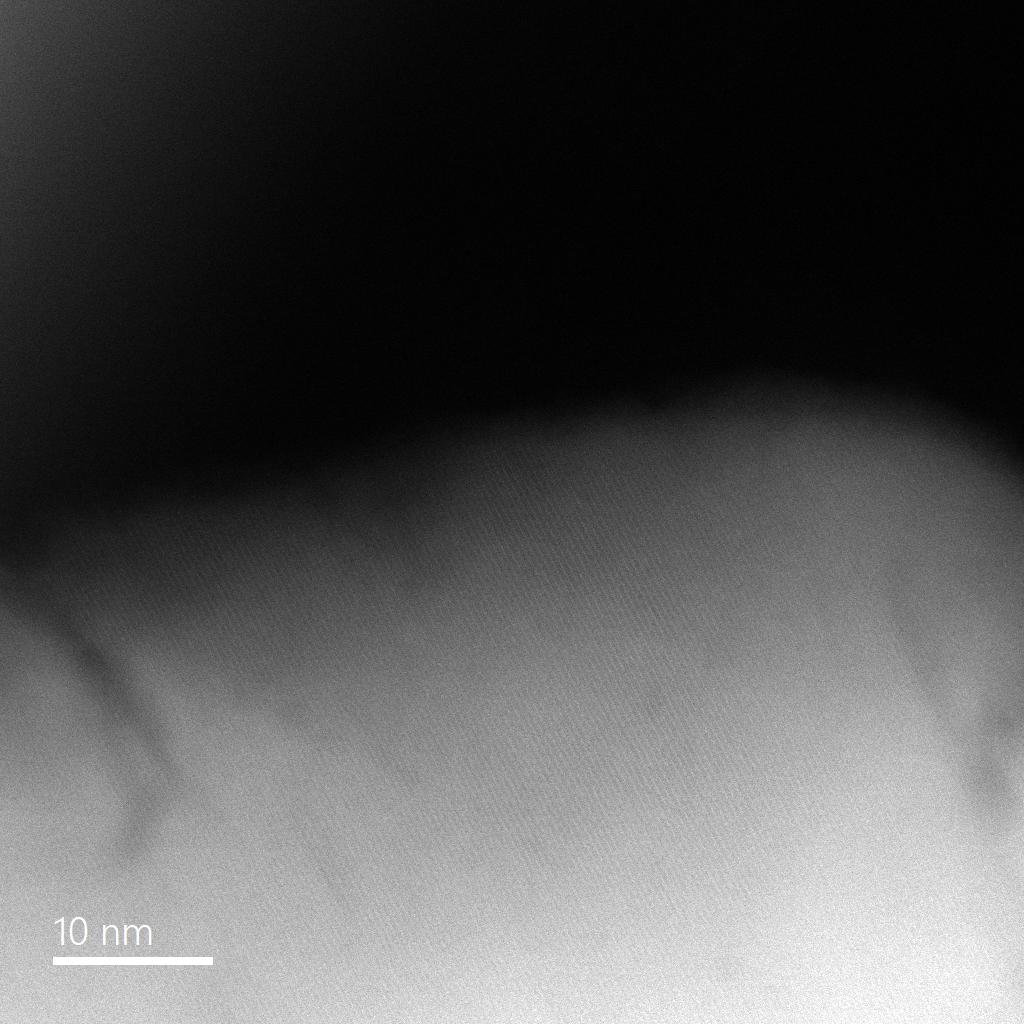

Supplement: Supplementary file 6 — Source Data [file 41467_2023_37212_MOESM6_ESM.zip › Raw_data/Figure2/Fig2a_SuperScan-HAADF-12_2019-06-15T160319.410230_1024x1024_21_Scalebar.jpg]

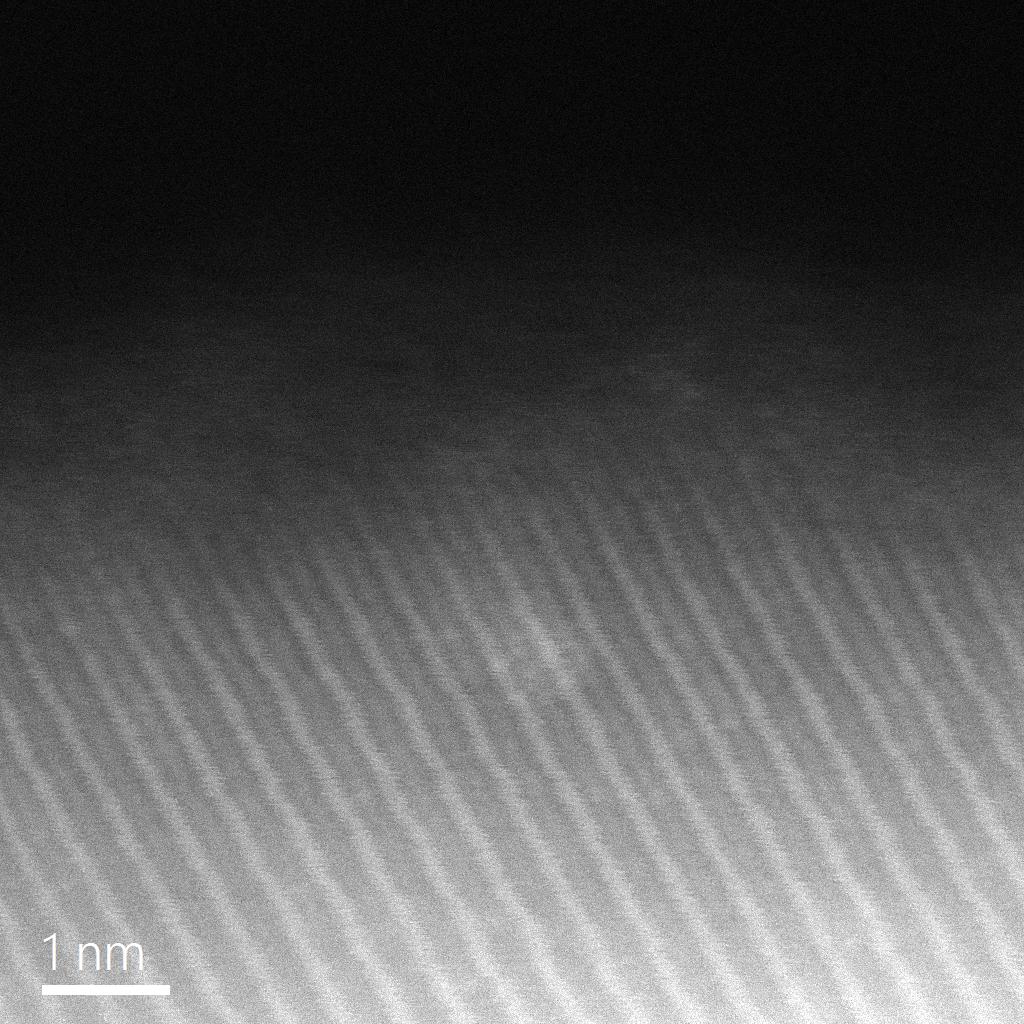

Supplement: Supplementary file 6 — Source Data [file 41467_2023_37212_MOESM6_ESM.zip › Raw_data/Figure2/Fig2g_SuperScan-HAADF-20_2019-06-15T202105.606219_1024x1024_3_scalebar.jpg]

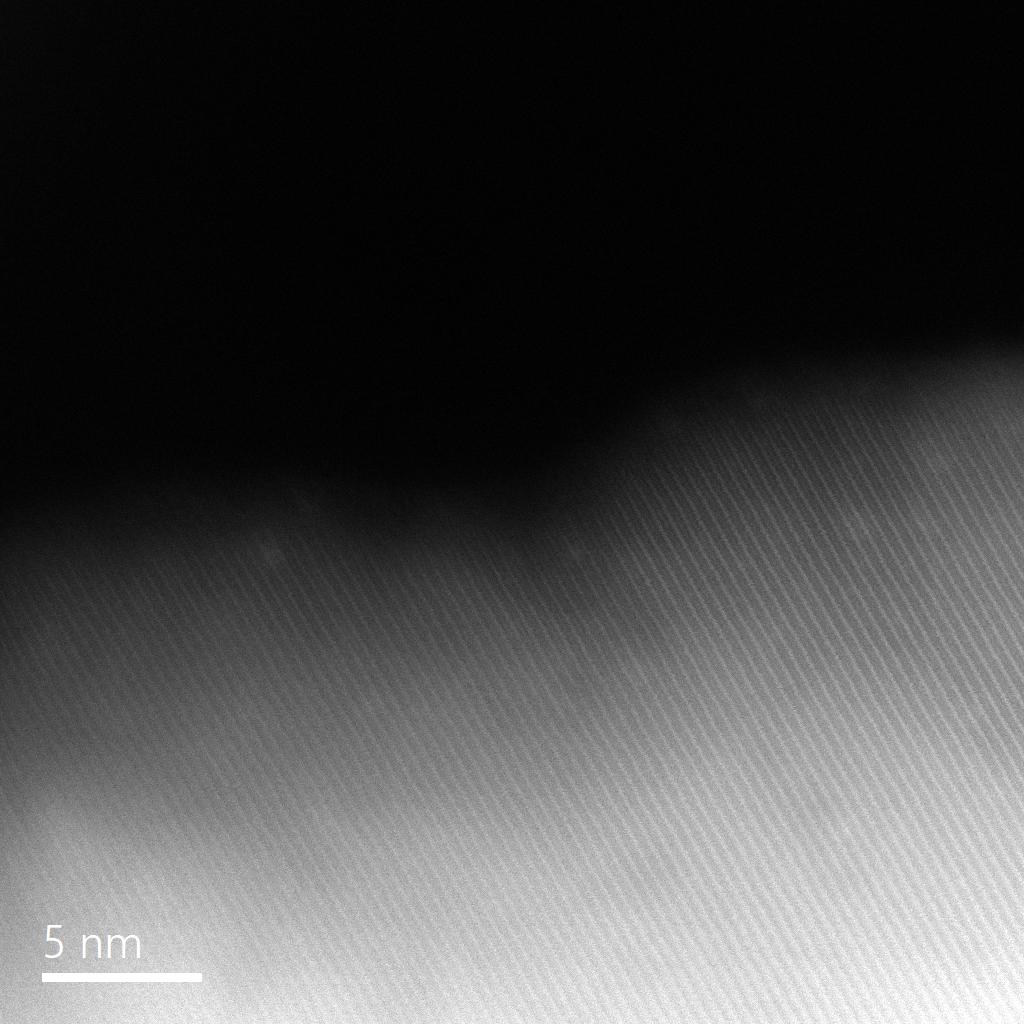

Supplement: Supplementary file 6 — Source Data [file 41467_2023_37212_MOESM6_ESM.zip › Raw_data/Figure2/Fig2f_750_SuperScan-HAADF-3_2019-06-15T214415.302613_1024x1024_5_scalebar.jpg]

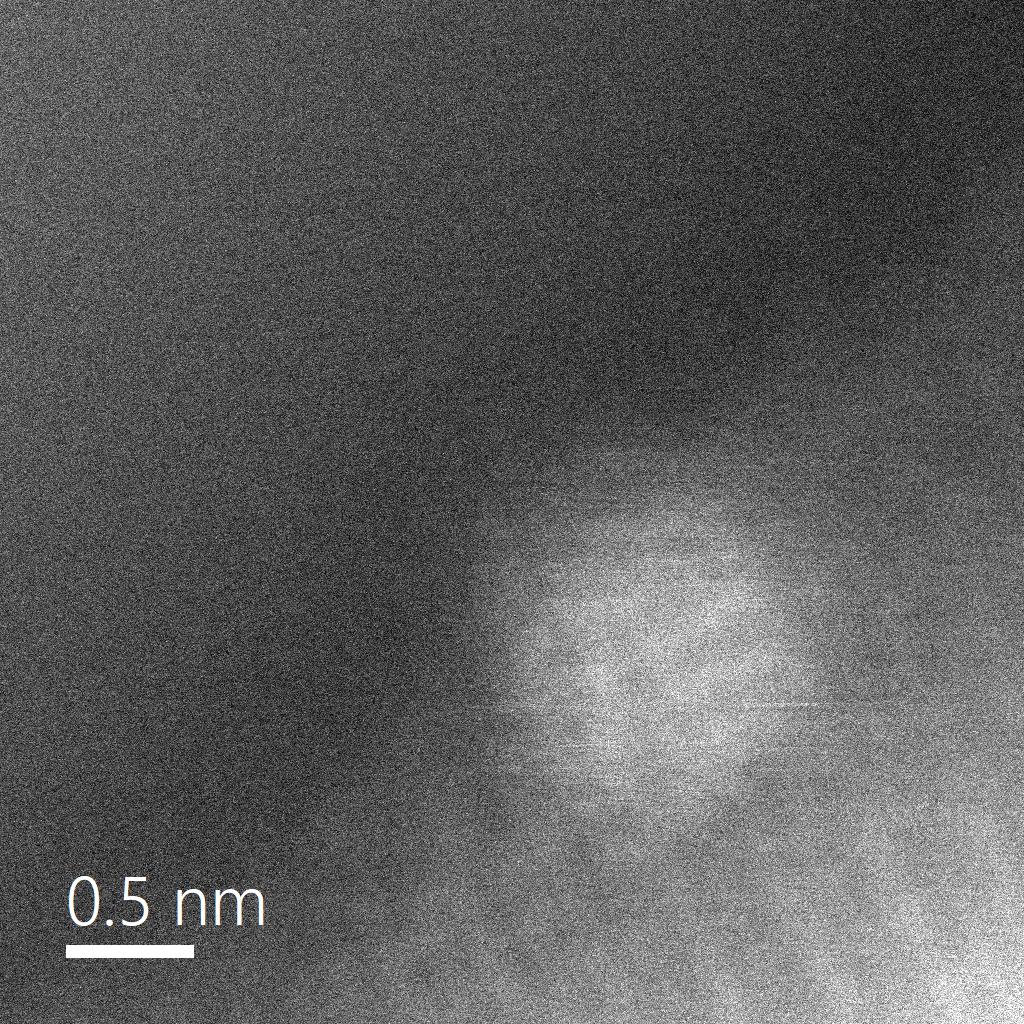

Supplement: Supplementary file 6 — Source Data [file 41467_2023_37212_MOESM6_ESM.zip › Raw_data/Figure2/Fig2h_SuperScan-HAADF-33_2019-06-15T210819.321298_1024x1024_4_scalebar.jpg]

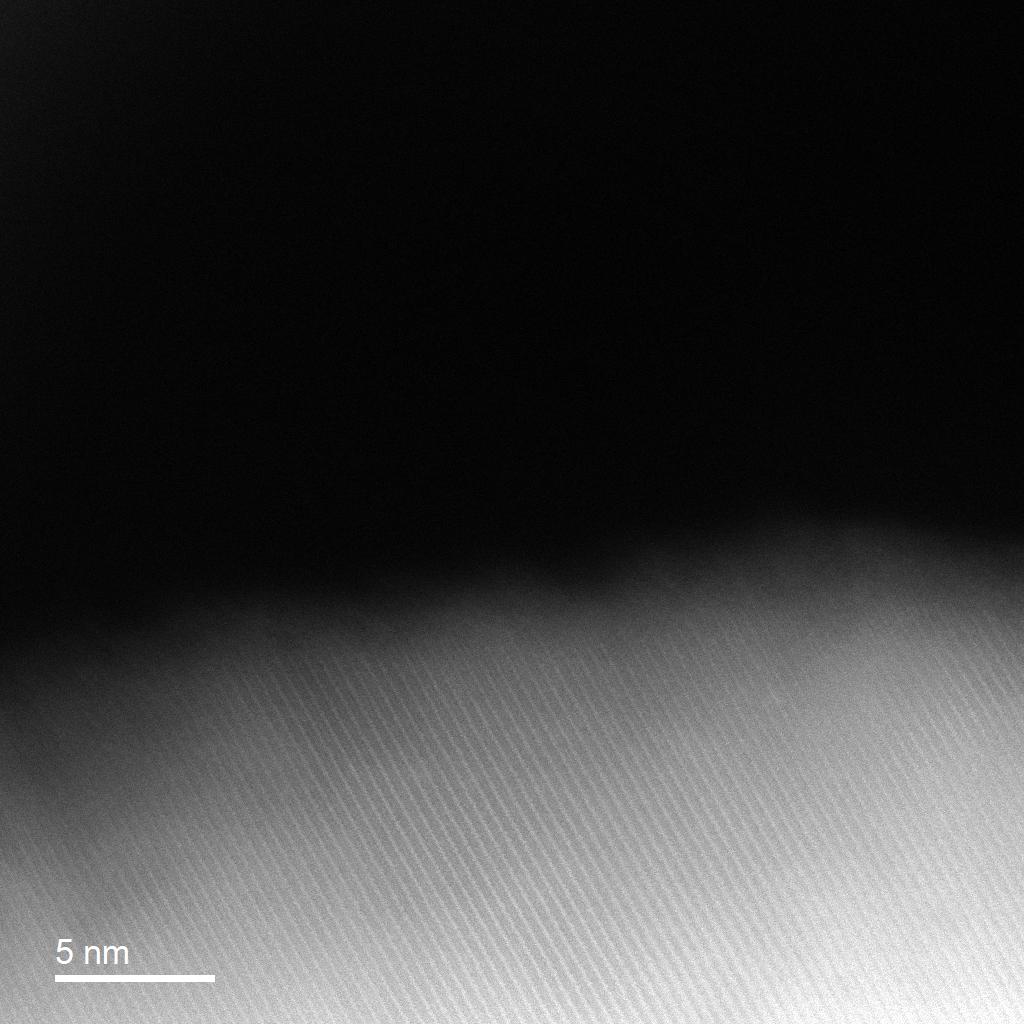

Supplement: Supplementary file 6 — Source Data [file 41467_2023_37212_MOESM6_ESM.zip › Raw_data/Figure2/Fig2b_SuperScan-HAADF-33_2019-06-15T173628.872929_1024x1024_0_scalebar.jpg]

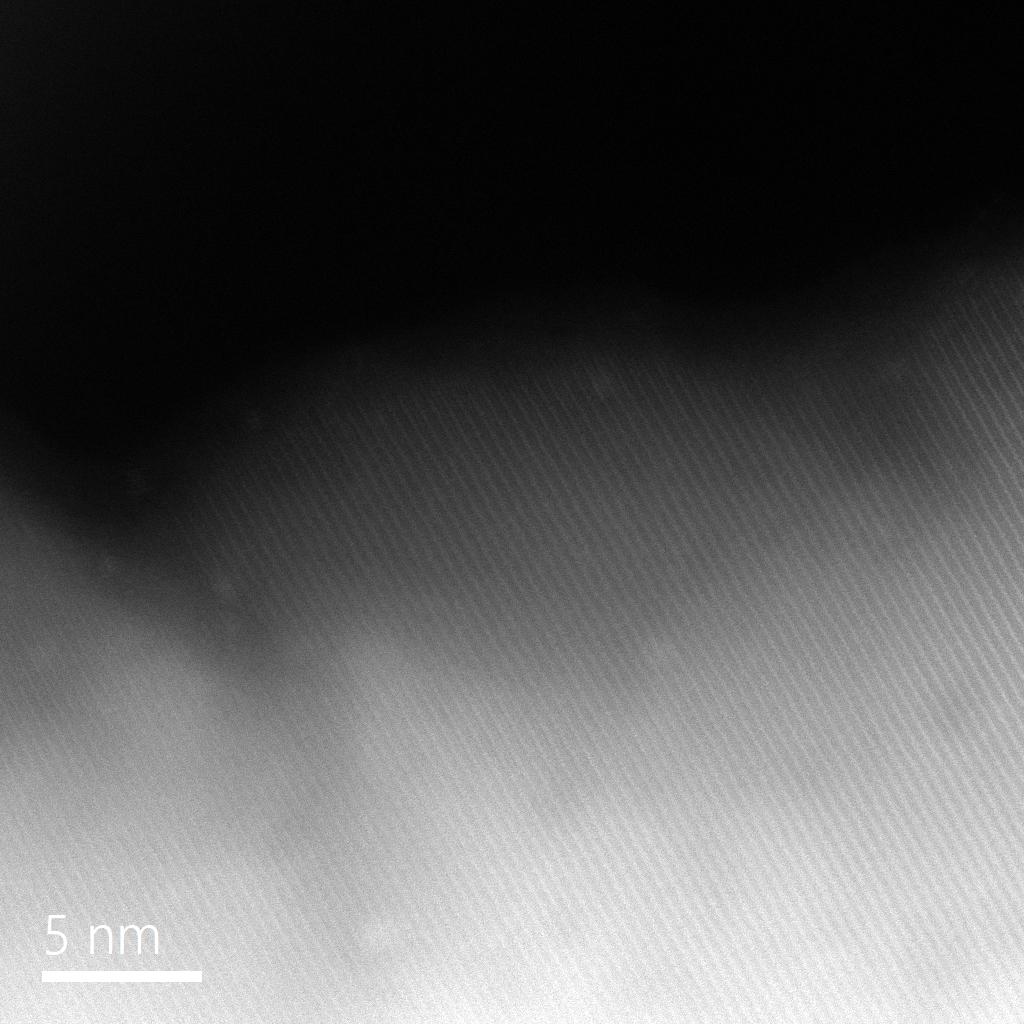

Supplement: Supplementary file 6 — Source Data [file 41467_2023_37212_MOESM6_ESM.zip › Raw_data/Figure2/Fig2d_700_2hSuperScan-HAADF-18_2019-06-15T201905.332339_1024x1024_5_scalebar.jpg]

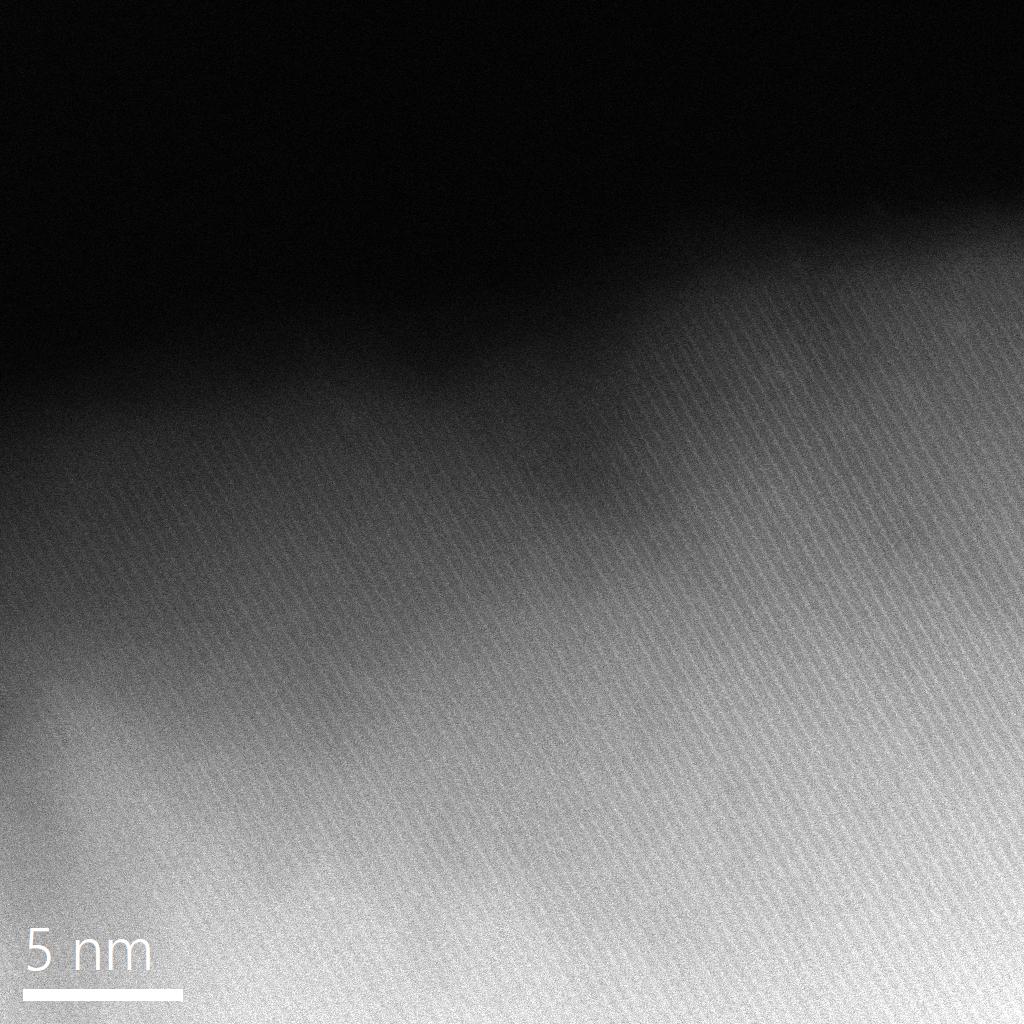

Supplement: Supplementary file 6 — Source Data [file 41467_2023_37212_MOESM6_ESM.zip › Raw_data/Figure2/Fig2c_asreached700_opt2_SuperScan-HAADF-13_scalebar.jpg]

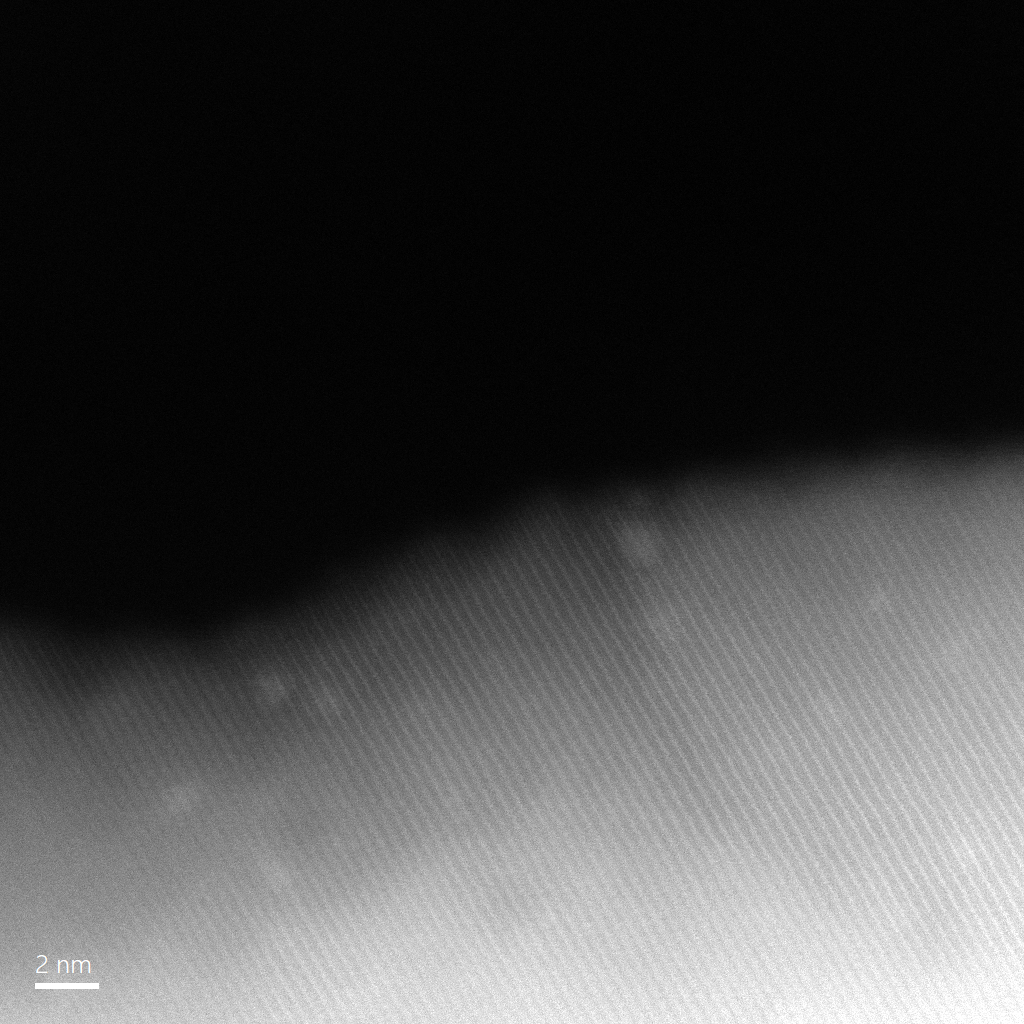

Supplement: Supplementary file 6 — Source Data [file 41467_2023_37212_MOESM6_ESM.zip › Raw_data/Figure3/Fig3b_875C_SuperScan-HAADF-48_2019-06-16T013757.464635_1024x1024_13.tif]

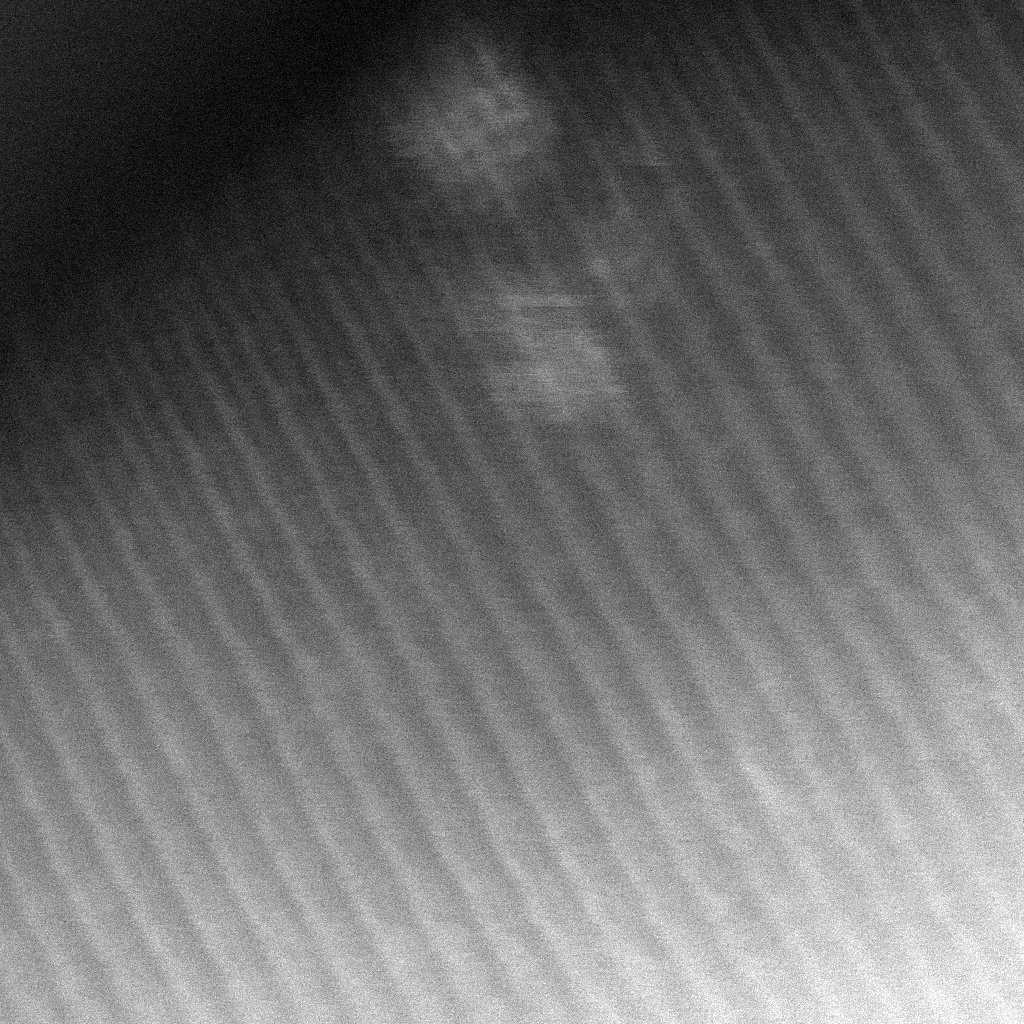

Supplement: Supplementary file 6 — Source Data [file 41467_2023_37212_MOESM6_ESM.zip › Raw_data/Figure3/Fig3a_775C_2_SuperScan-HAADF-19_2019-06-15T224622.556800_1024x1024_4_NOsb.tif]

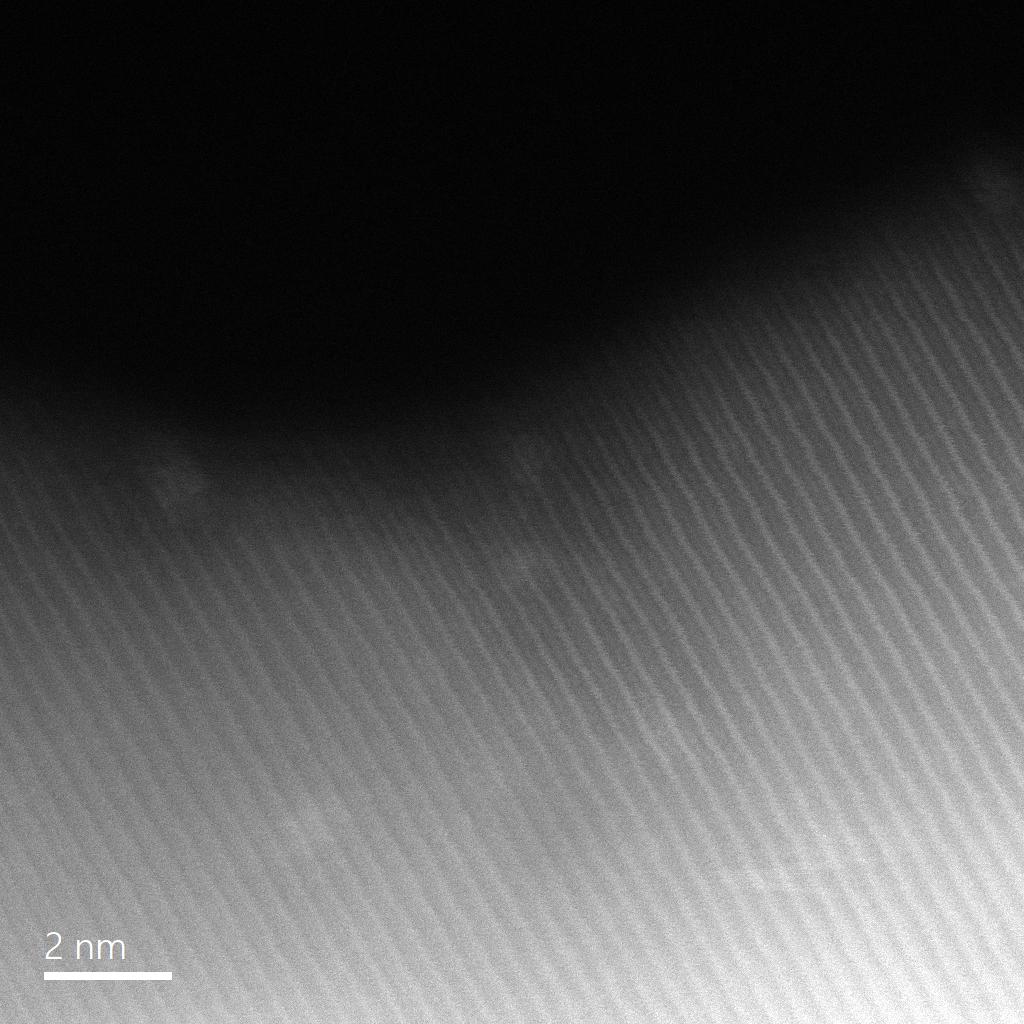

Supplement: Supplementary file 6 — Source Data [file 41467_2023_37212_MOESM6_ESM.zip › Raw_data/Figure3/Fig3b_NP1_825_highmag_SuperScan-HAADF-33_2019-06-16T002334.521369_1024x1024_8.jpg]

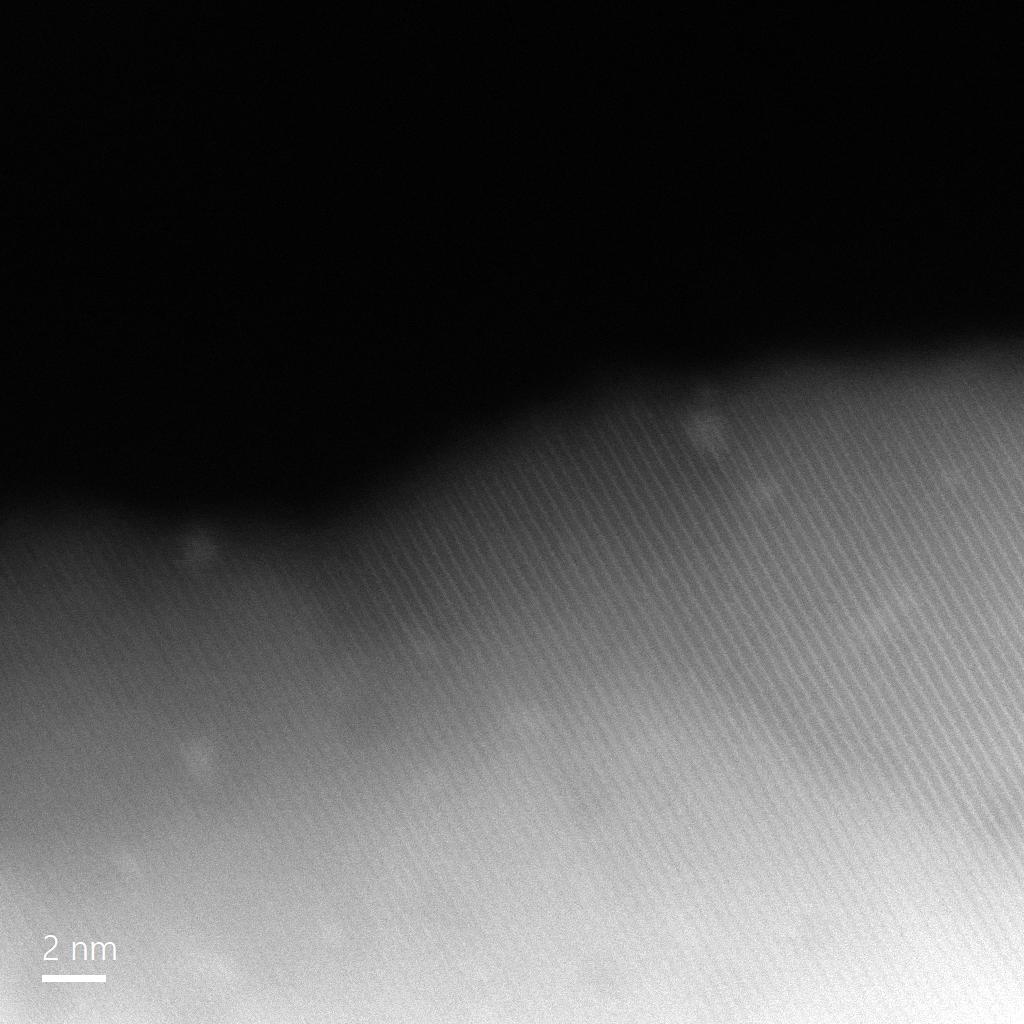

Supplement: Supplementary file 6 — Source Data [file 41467_2023_37212_MOESM6_ESM.zip › Raw_data/Figure3/Fig3B_NP1_850_SuperScan-HAADF-44_2019-06-16T010628.187574_1024x1024_17.jpg]

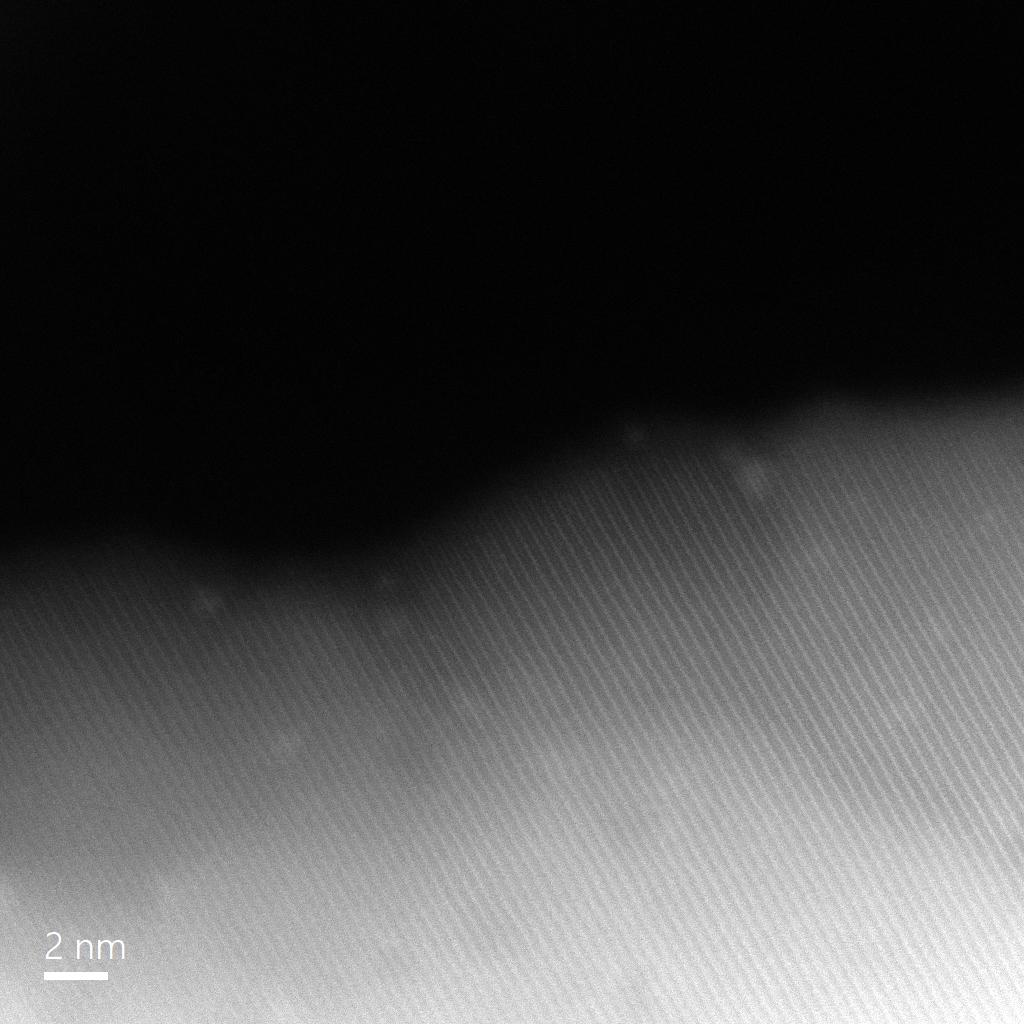

Supplement: Supplementary file 6 — Source Data [file 41467_2023_37212_MOESM6_ESM.zip › Raw_data/Figure3/Fig3b_NP1_825_lowmag_SuperScan-HAADF-35_2019-06-16T002525.717729_1024x1024_6.jpg]

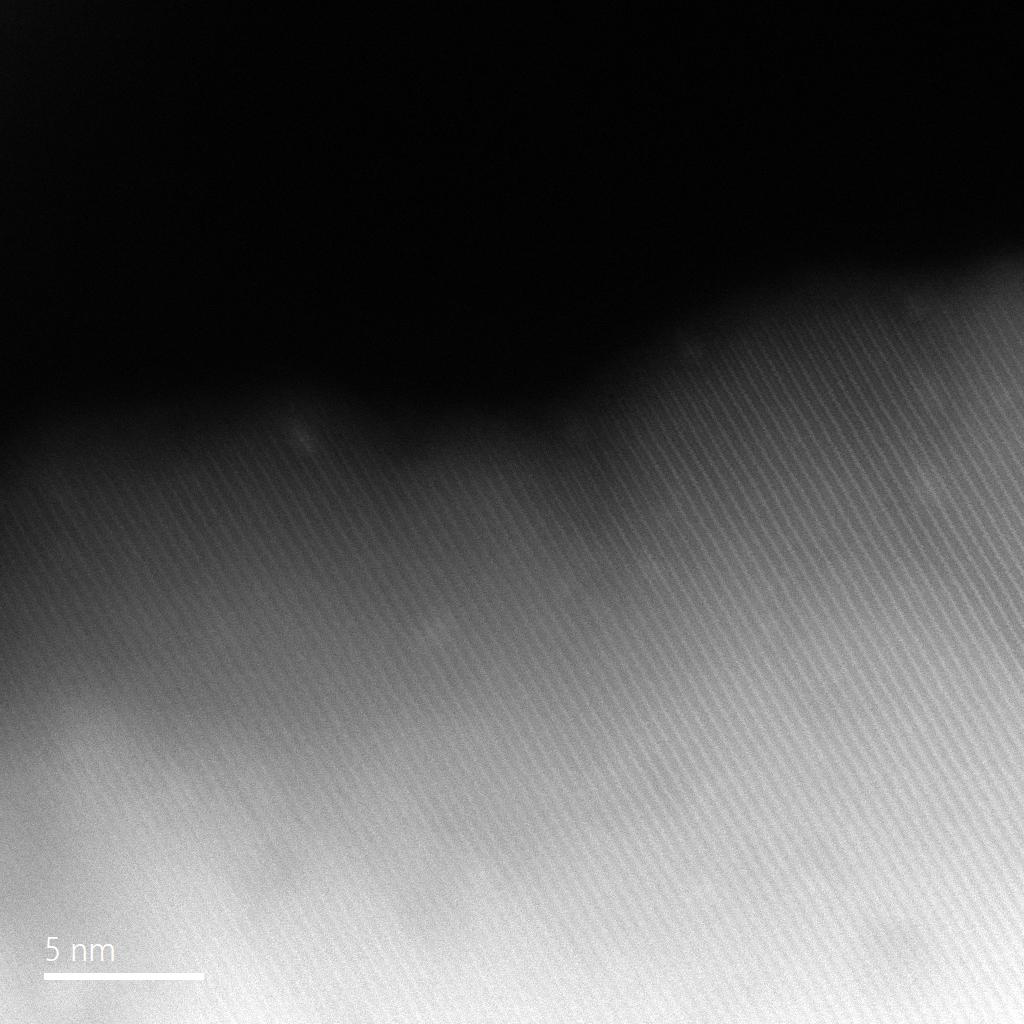

Supplement: Supplementary file 6 — Source Data [file 41467_2023_37212_MOESM6_ESM.zip › Raw_data/Figure4/800C_SuperScan-HAADF-26_2019-06-15T232948.693862_1024x1024_6_sb.jpg]

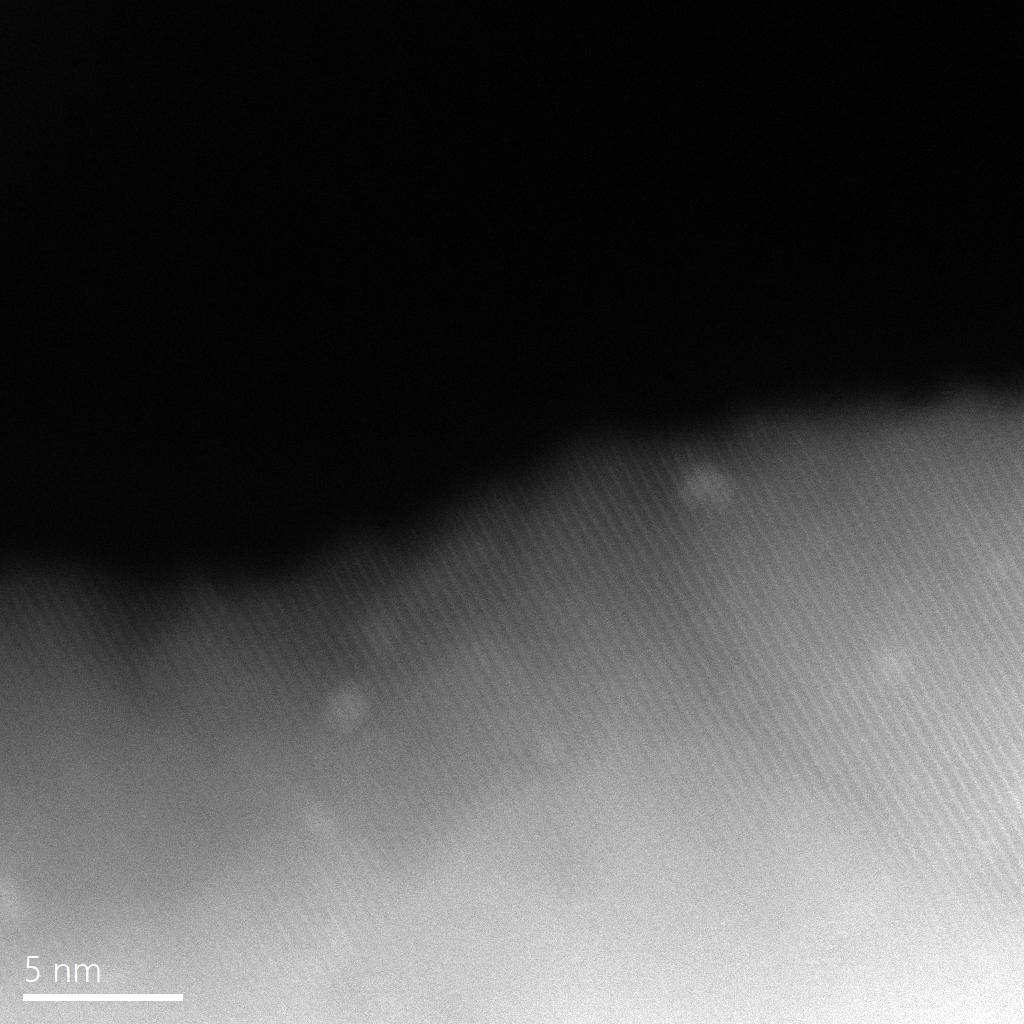

Supplement: Supplementary file 6 — Source Data [file 41467_2023_37212_MOESM6_ESM.zip › Raw_data/Figure4/900C_SuperScan-HAADF-52_2019-06-16T021005.080888_1024x1024_9_sb.jpg]

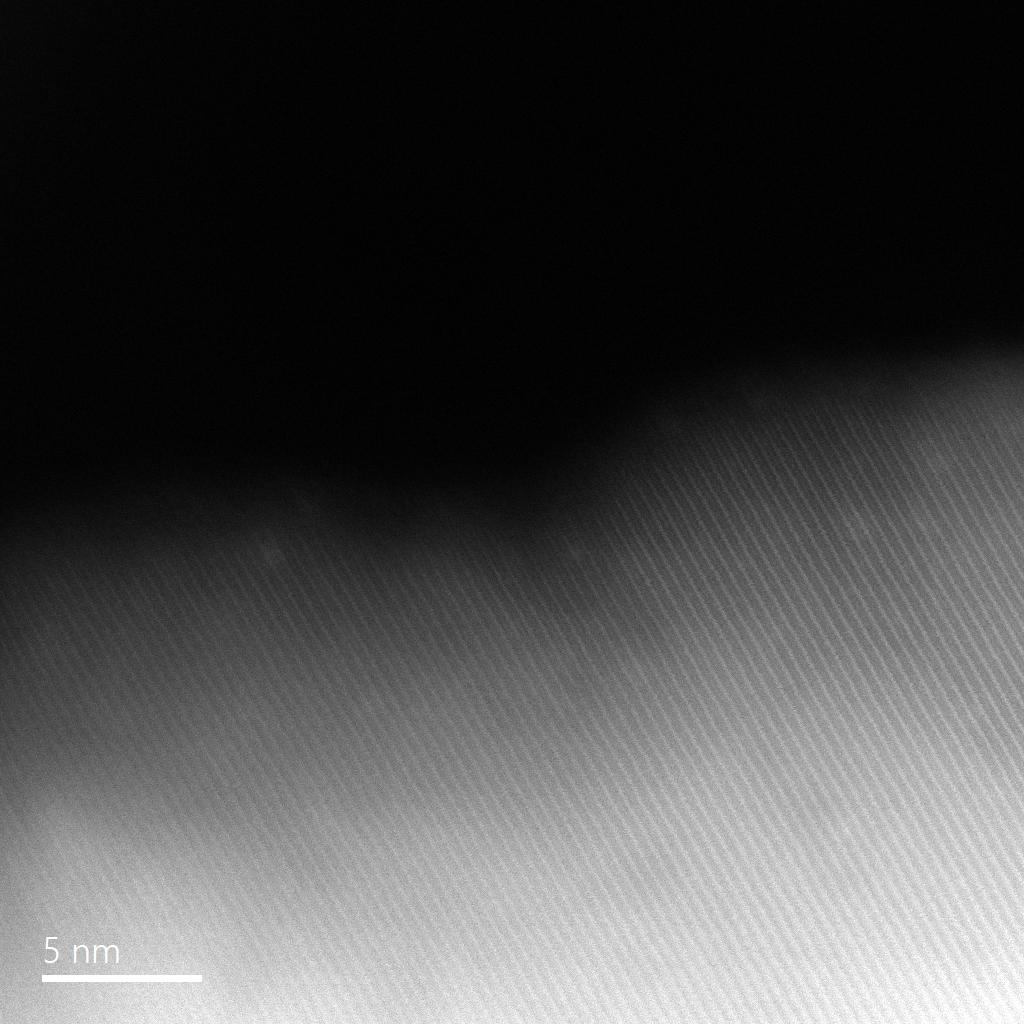

Supplement: Supplementary file 6 — Source Data [file 41467_2023_37212_MOESM6_ESM.zip › Raw_data/Figure4/750C_SuperScan-HAADF-3_2019-06-15T214415.302613_1024x1024_5_sb.jpg]

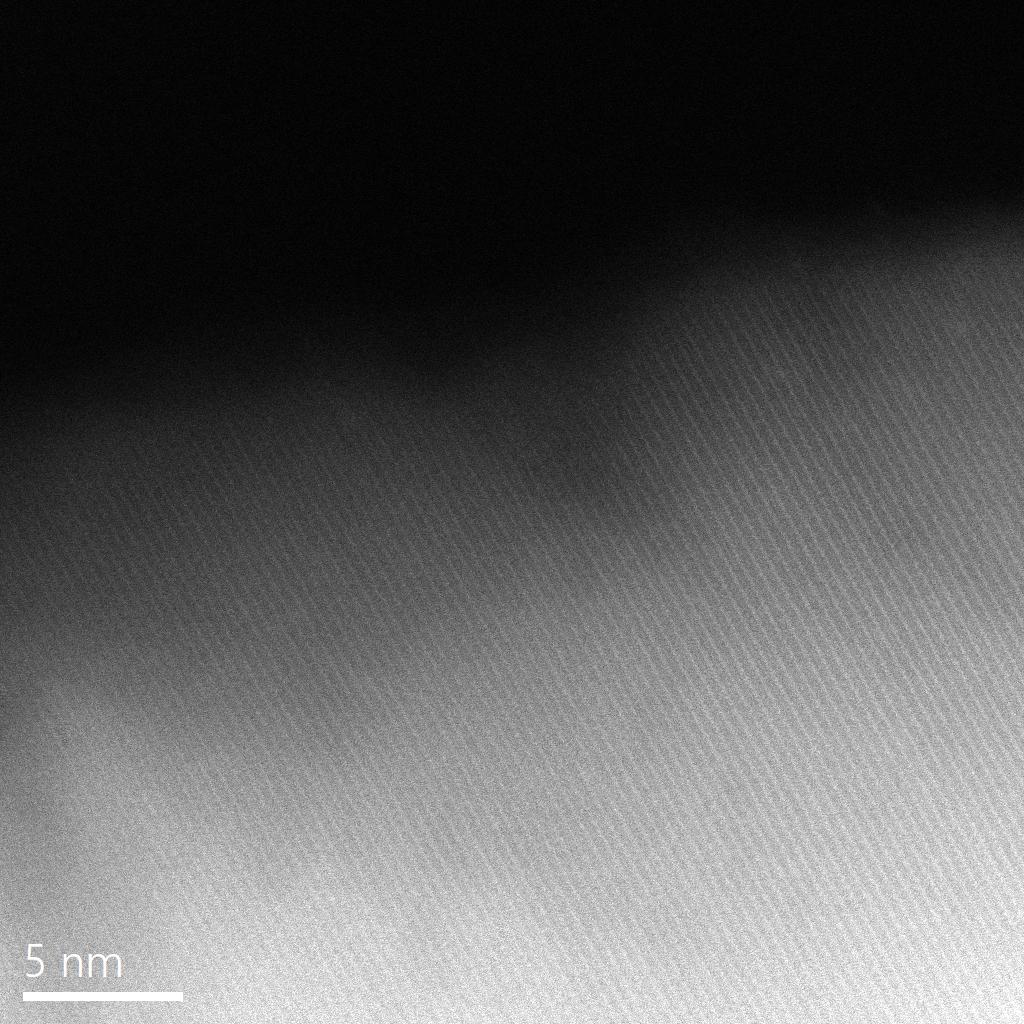

Supplement: Supplementary file 6 — Source Data [file 41467_2023_37212_MOESM6_ESM.zip › Raw_data/Figure4/700C_SuperScan-HAADF-13_2019-06-15T181636.148990_1024x1024_10_sb.jpg]

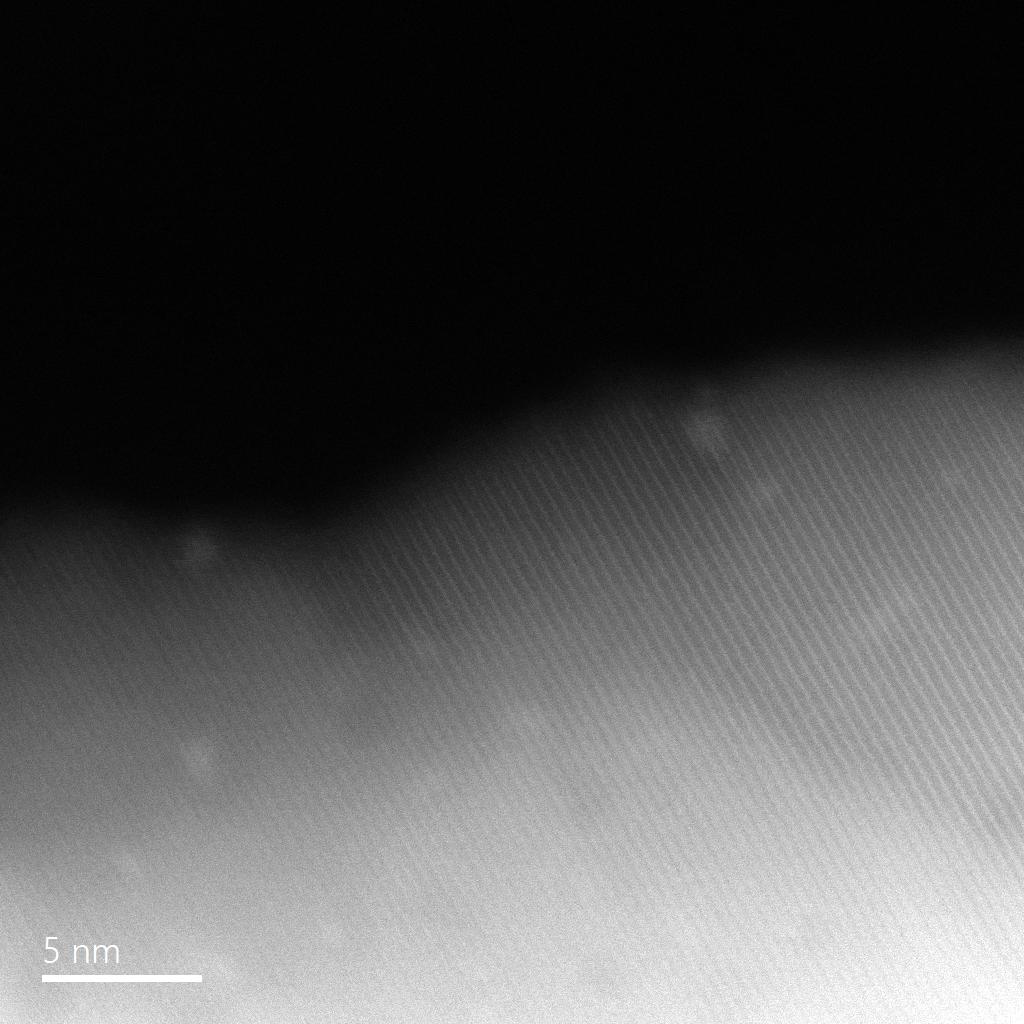

Supplement: Supplementary file 6 — Source Data [file 41467_2023_37212_MOESM6_ESM.zip › Raw_data/Figure4/850C_SuperScan-HAADF-44_2019-06-16T010628.187574_1024x1024_17_sb.jpg]

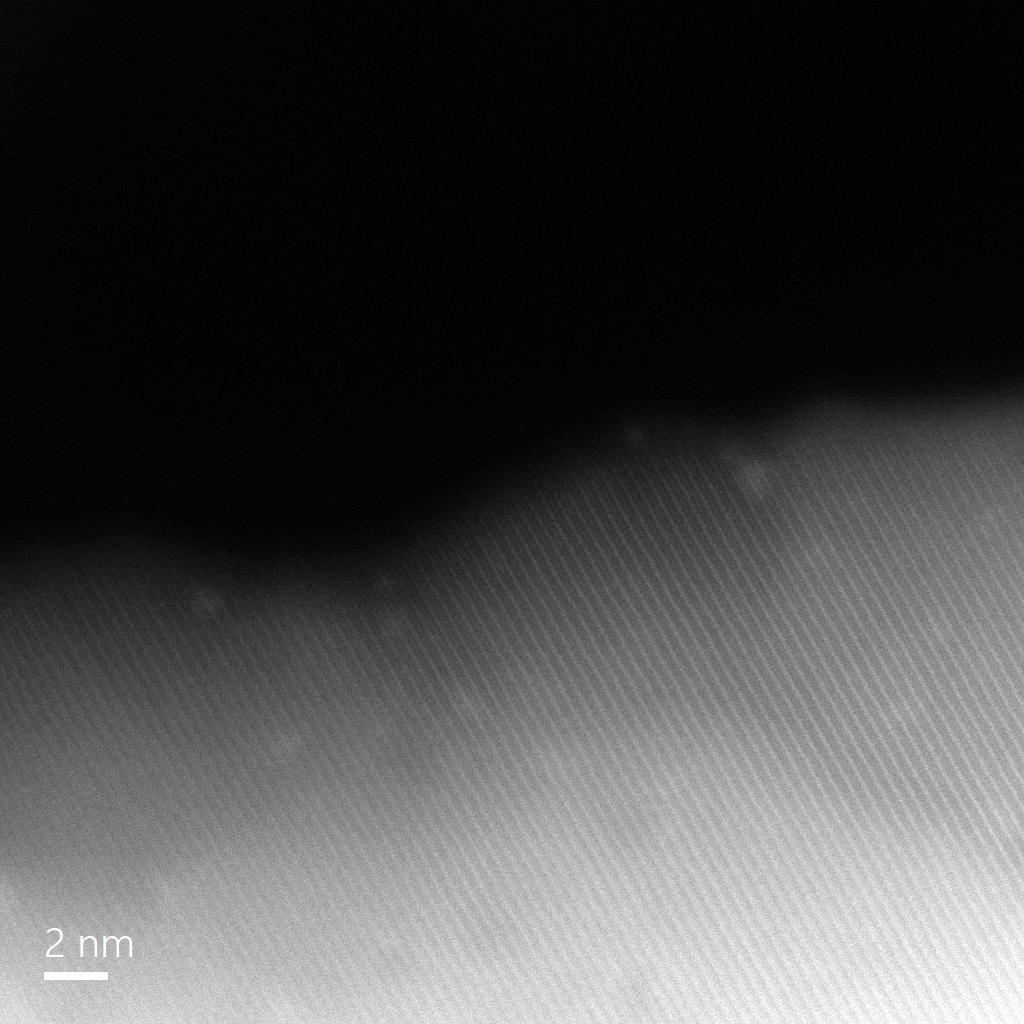

Supplement: Supplementary file 6 — Source Data [file 41467_2023_37212_MOESM6_ESM.zip › Raw_data/SI Figure9/825C_SuperScan-HAADF-35_2019-06-16T002525.717729_1024x1024_6.jpg]

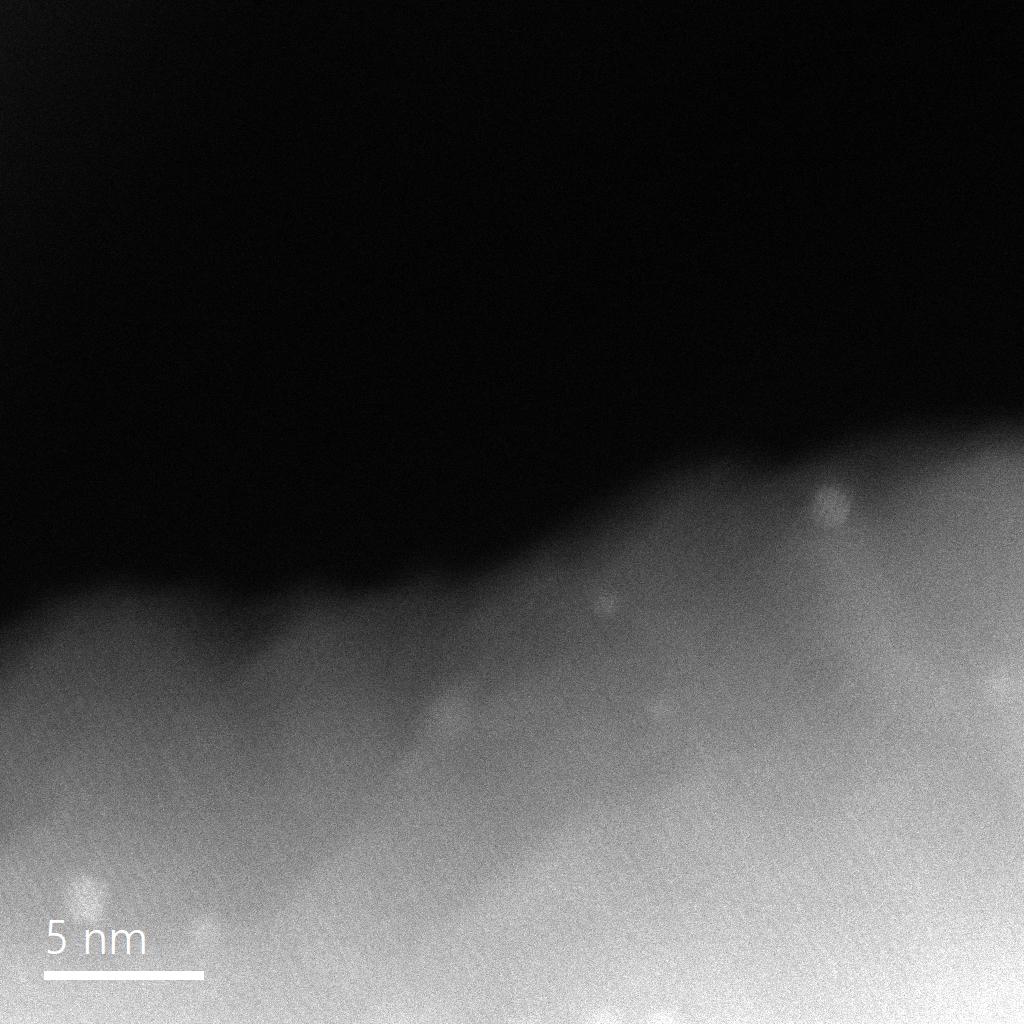

Supplement: Supplementary file 6 — Source Data [file 41467_2023_37212_MOESM6_ESM.zip › Raw_data/Figure6/6c_925C_SuperScan-HAADF-52_2019-06-16T175648.087629_1024x1024_135.jpg]

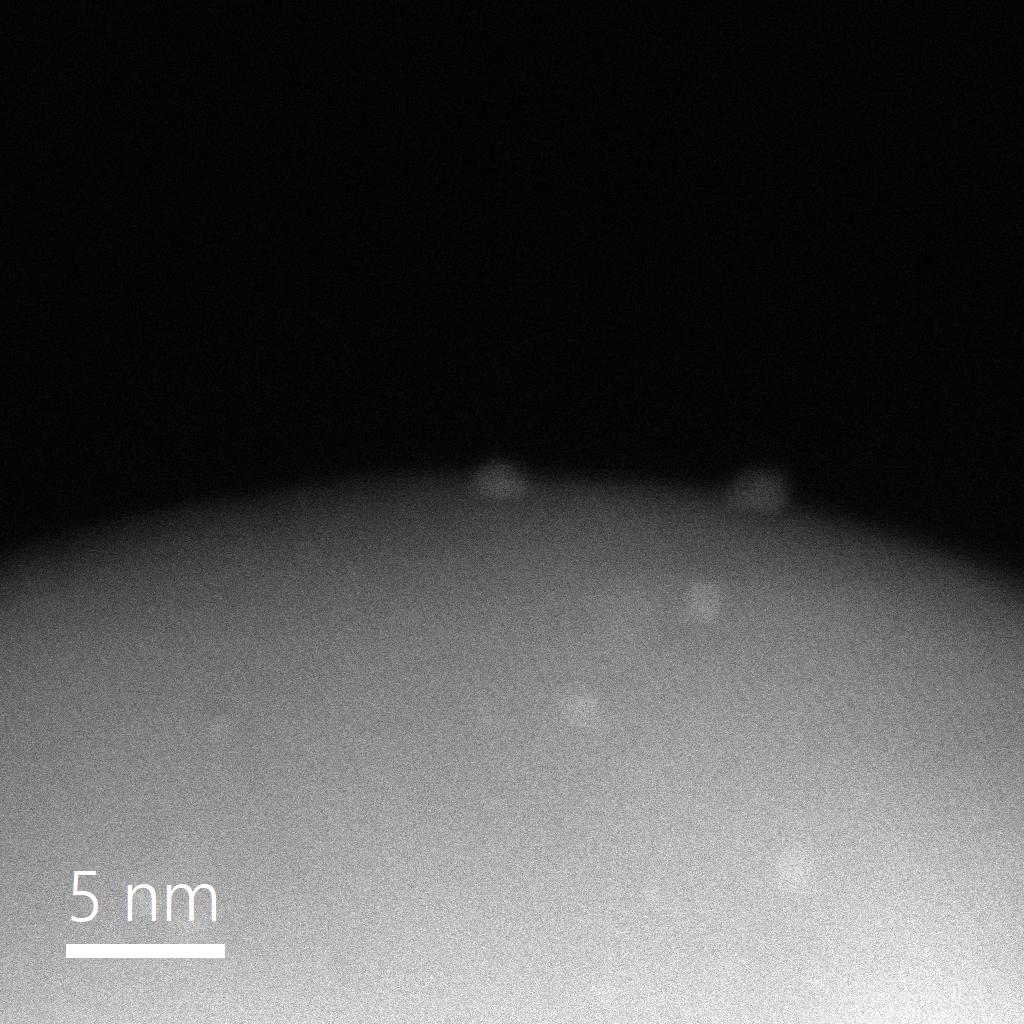

Supplement: Supplementary file 6 — Source Data [file 41467_2023_37212_MOESM6_ESM.zip › Raw_data/Figure6/6g_975C_SuperScan-HAADF-21_2019-06-16T194632.182218_1024x1024_90.jpg]

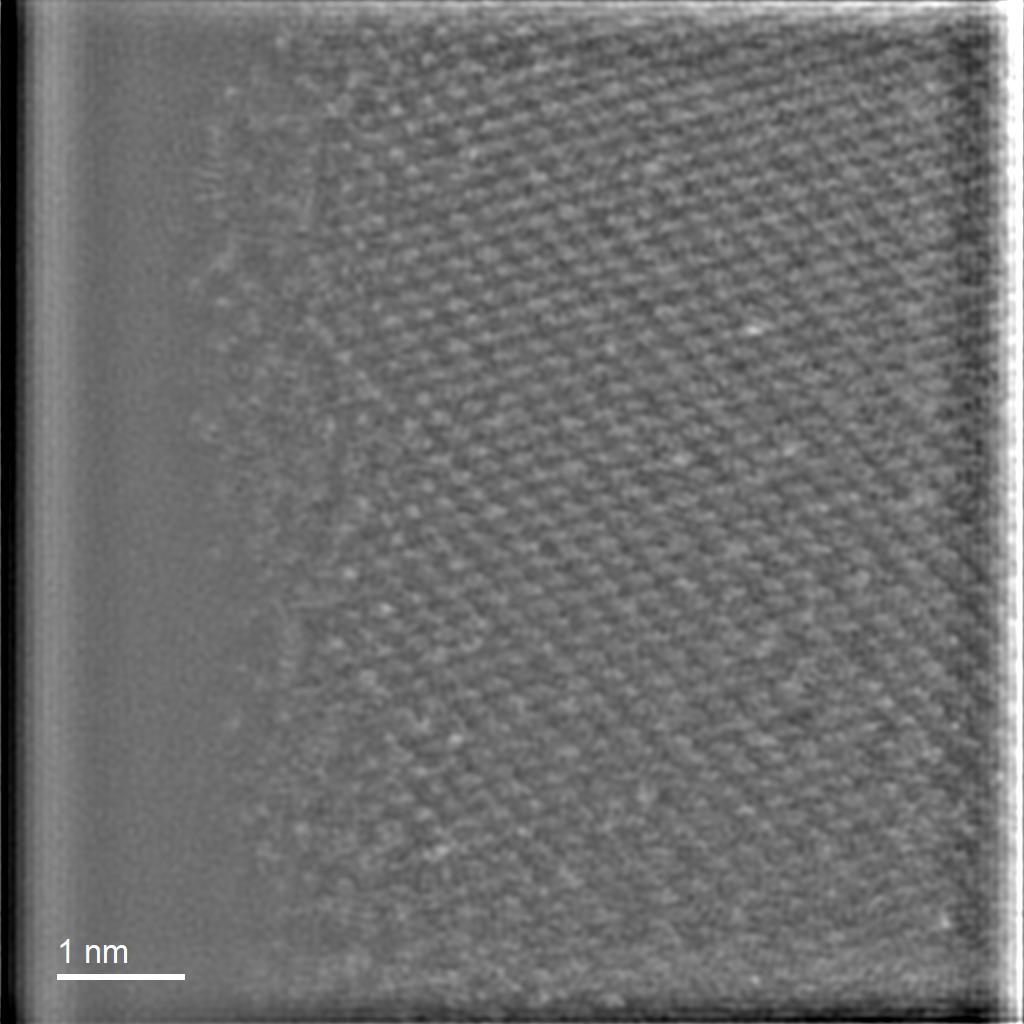

Supplement: Supplementary file 6 — Source Data [file 41467_2023_37212_MOESM6_ESM.zip › Raw_data/SI Figure1/IFFT of Untitled HAADF94 RT 29Feb2020.jpg]

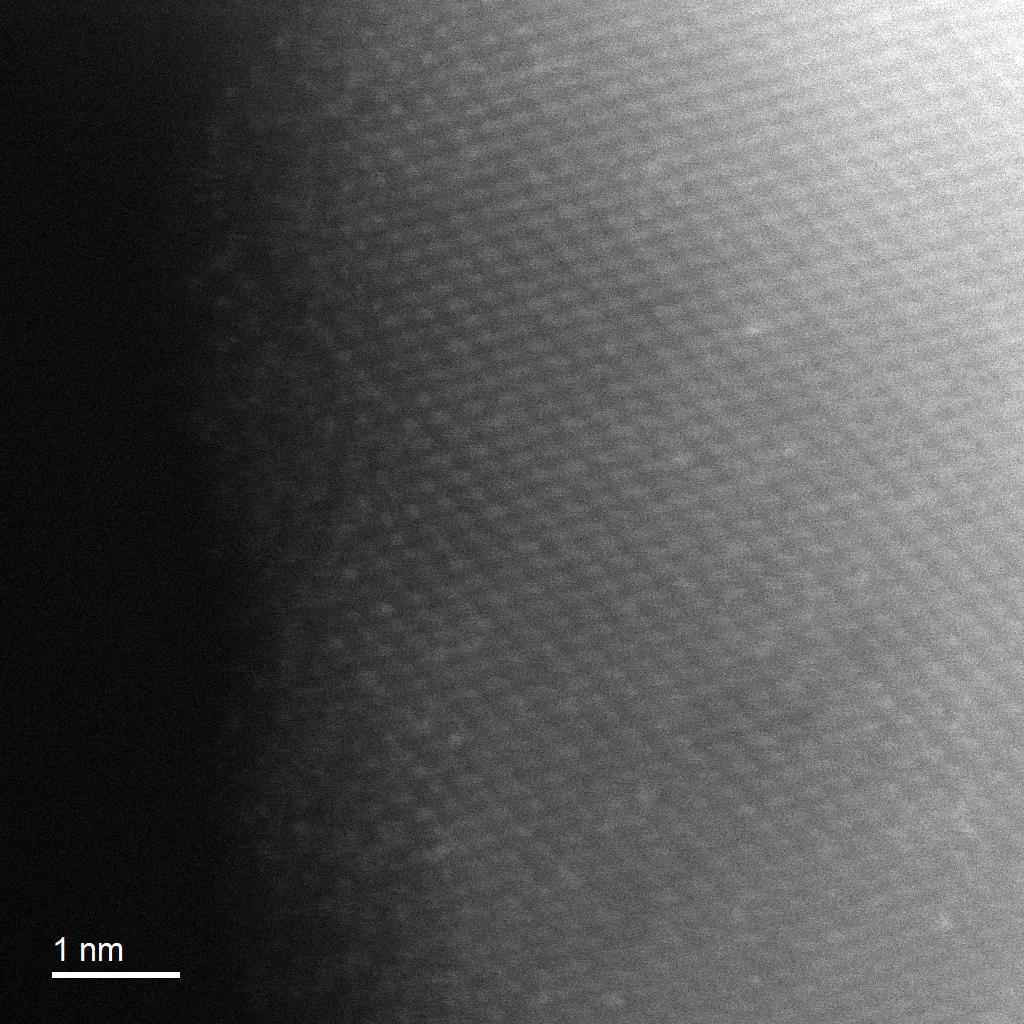

Supplement: Supplementary file 6 — Source Data [file 41467_2023_37212_MOESM6_ESM.zip › Raw_data/SI Figure1/SuperScan-HAADF-94_2020-02-29T182323.315727_1024x1024_26.jpg]
